# Evaluation of 99 Pesticide Residues in Major Agricultural Products from the Western Highlands Zone of Cameroon Using QuEChERS Method Extraction and LC-MS/MS and GC-ECD Analyses

**DOI:** 10.3390/foods7110184

**Published:** 2018-11-07

**Authors:** Joseph H. Y. Galani, Michael Houbraken, Abukari Wumbei, Joseph F. Djeugap, Daniel Fotio, Pieter Spanoghe

**Affiliations:** 1 Department of Agriculture and Veterinary Medicine, Université des Montagnes, P.O. Box 208, Bangangté, Cameroon; 2 Department of Plants and Crops, Faculty of Bioscience Engineering, Ghent University, Coupure Links 653, 9000 Ghent, Belgium; michael.houbraken@ugent.be (M.H.); abukari.wumbei@ugent.be (A.W.); pieter.spanoghe@ugent.be (P.S.); 3 Department of Plant Protection, Faculty of Agronomy and Agricultural Sciences, University of Dschang, P.O. Box 222, Dschang, Cameroon; jdjeugapfovo@yahoo.fr; 4 Inter-States Pesticides Committee of Central Africa, P.O. Box 16344, Yaounde, Cameroon; danfotio@yahoo.co.uk

**Keywords:** food safety, pesticide residues, QuEChERS method, staple food, Cameroon

## Abstract

There is no information available on pesticide residue levels in major food commodities harvested in Cameroon, especially from the western highlands region, the food basket of the country. Hence, this study evaluated the residues of 99 pesticides in 72 samples of 12 agricultural products collected in the region, using QuEChERS (Quick, Easy, Cheap, Effective, Rugged, and Safe) method extraction, and analyzed by liquid chromatography tandem mass spectrometry (LC-MS/MS) and gas chromatography with electron capture detection (GC-ECD). This method was suitable for detecting the targeted compounds: For 81 pesticides by LC-MS/MS, the limit of quantification (LOQ) was between 0.0004 and 0.0537 mg/kg; and for 18 halogenated pesticides by GC-ECD, it ranged from 0.0012 to 0.2180 mg/kg. The residues of 62 pesticides, including 12 banned compounds, were found in the samples. Insecticides (39.7%) were the most prevalent group, with all the samples containing at least one pesticide. Twenty-one pesticides (34.4%) exceeded their European Union maximum residue limits (MRLs) and 22 pesticides (34.4%) were found in all 6 sampling locations. Malathion and *p*,*p*′-DDT were the most distributed pesticides, found in almost all the samples and sampling sites. Food items with the highest rates of positive results were chili pepper (23.2%), white pepper (20.2%), kidney beans (17.3%), and soybeans (17.2%). Samples with residues above their MRLs represented 38% of all the positive analyses; chili pepper (6.4%) and kidney beans (5.5%) were found to have the most residues above their MRLs. The most critical food commodities were kidney beans, soybeans, chili pepper, and maize. This data presents scientific evidence that investigation into continuous monitoring and good regulation of pesticide usage in Cameroon is needed, and paves the way for health risks analysis.

## 1. Introduction

To protect crops against pests and pathogens, over a thousand crop protection products from a broad range of classes are widely used worldwide in various combinations, at different stages of cultivation, and during postharvest storage. However, besides the unwanted side effects on the environment, direct toxicity to users, and development of resistance by pathogens and pests associated to the use of certain pesticides, pesticide residues that remain in the food supply could pose a risk for human health because of their potential sub-acute and chronic toxicity [1]. Pesticide residues can be found in different food items, such as dairy products, cereals, fruits, vegetables, and cash crops, and obsolete pesticides have been documented as one of the major problems in Africa [2].

In Cameroon, pesticides are largely used by farmers and traders to protect their growing plants and products in the field and during storage [3,4,5,6,7]. Despite the numerous advantages of pesticide use in agriculture, there is a need for scientific evaluation and control of these products. It is known that pesticide residues can be found in all environmental compartments, but the highest risk for consumers is through consumption of residues in food [8]. In developing countries like Cameroon, there is an increased concern in the dietary risk linked to increased use of crop protection products. However, only minimal emphasis have been put on assessing how the growing use of pesticides can impact food safety. Ten years ago, Gimou et al. [9] revealed low dietary exposure to pesticide residues in the capital city, Yaoundé. But more recently it was found that 75% of maize, cowpea, and millet samples from northern Cameroon contained pesticide residues above the maximum residue limits (MRLs) [10], and high amounts of organophosphorous pesticide residues were found in stored cowpea and two by-products [5], revealing a potential human dietary risk related to consumption of these grains. 

Other information demonstrates a high possible exposure of consumers due to intensive utilization and limited knowledge about pesticide use in the country. Recent studies [4] suggested that the male farmers of Djutitsa in West Cameroon are exposed to agro-pesticides due to improper personal protective equipment (PPE), and this exposure may impair their reproductive function. Inappropriate use of pesticides by Cameroonian farmers has been documented in many studies from different parts of the country [4,7,10,11,12,13]. This was because farmers did not receive sufficient training on pesticide application and proper assistance from agricultural extension agents [7], which could result in high levels of pesticide residues in local foods. In December 2016, the Cameroon Government prohibited the importation, commercialization, and use of metalaxyl-based pesticides, which were intensively used in cocoa to control black pod disease. This measure was justified by the rejection of Cameroonian cocoa on the international market, due to the presence of metalaxyl residues beyond the 0.1 mg/kg MRL [14] in cocoa beans originating from Cameroon [15]. Additionally, a high number and various types of obsolete pesticides accumulated over the years were found in the country [16], and because of limited control of pesticide usage, they could be a source of severe acute or chronic pollution [9]. Due to their cheap price on the black market and limited control measures, they can be illegally used on crops and produce [17]. 

The most common food items produced in Cameroon include cocoa, coffee, palm oil, maize, beans, cassava, groundnuts, plantains, and bananas. Other items like soybeans, chili pepper, Egusi seeds, white pepper, and Bambara nuts largely produced and consumed in Cameroon are also found in markets of neighboring countries, which mostly depend on Cameroon for their food supply. The western highlands of Cameroon, from where the vast majority of these agricultural products originate, is considered as the food basket of the country, and by extension, of the whole Central African region [18]. There is no study investigating pesticide contamination levels of agricultural products from this high production region of Cameroon, hence this work was planned.

Among the various methods of pesticide residue determination in food items, the QuEChERS method (Quick, Easy, Cheap, Effective, Rugged, and Safe) with a dispersive solid-phase extraction (d-SPE) clean-up has been documented to give a better recovery compared to classical techniques. The QuEChERS method has the advantages of high recovery, high sample throughput, low solvent and glassware usage, less labor and bench space, lower reagent costs, ruggedness, and low worker exposure [19,20]. Thus, analytical chemists now prefer to use the QuEChERS method with a d-SPE clean-up when required; it is streamlined and effective for analysis of diverse residues in food matrices [2,21,22]. The present study analyzes and validates the residues of 99 pesticides in 72 samples of 12 agricultural products collected in the western highlands of Cameroon, using the QuEChERS method as the extraction and clean-up technique, and liquid chromatography tandem mass spectrometry (LC-MS/MS) and gas chromatography with electron capture detection (GC-ECD) for detection.

## 2. Materials and Methods

### 2.1. Reagents

Analytical grade reagents of above 99% purity were used in the experiments. UPLC-grade acetonitrile and hexane were procured from VWR Chemicals (Leuven, Belgium). Anhydrous magnesium sulphate, disodium hydrogen sesquihydrate, trisodium citrate dehydrate, sodium chloride, and the pesticide active ingredient standards were purchased from Sigma–Aldrich (Bornem, Belgium). Fifteen-milliliter d-SPE tubes were obtained from Waters (Zellik, Belgium). Water was produced locally though a Milli-Q purification system.

### 2.2. Samples Collection

**Figure 1:**
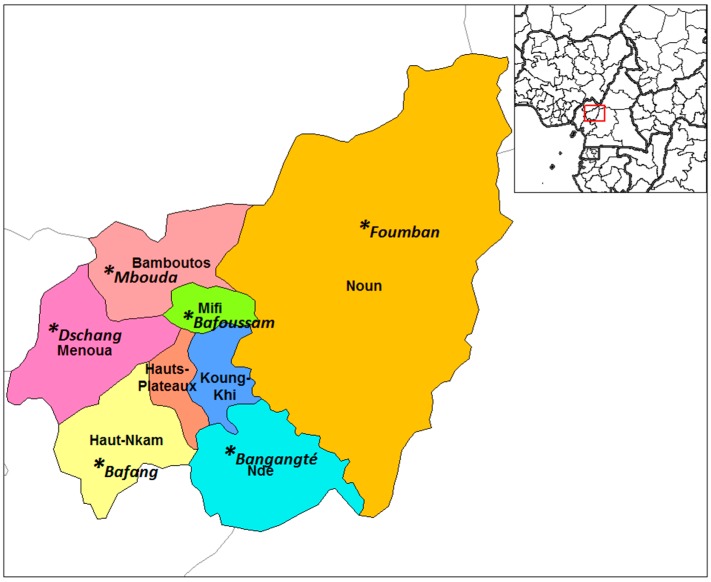
Map of the West Region of Cameroon showing the administrative divisions and major towns where samples were collected.

A total of 72 dried samples were collected in March 2017 from local markets of 6 major towns in the West Region of Cameroon: Bafang, Bangangté, Bafoussam, Dschang, Foumban, and Mbouda (Figure 1). They belonged to 12 agricultural products; namely, 8 groundnut samples, 6 soybean, 10 kidney bean, 6 black bean, 7 cowpea, 6 chili pepper, 7 Egusi seeds, 4 coffee beans, 2 cocoa beans, 11 maize, 2 white pepper, and 3 Bambara nuts. Interviews of the wholesalers during sample collection revealed that each food batch was made of a pool of small lots originating from different farmers of the neighboring villages. Therefore, they were considered as representative samples. Approximately 200 g of each sample was collected in a hard paper envelope within polyethylene plastic bag, sealed, labelled, transported to the Laboratory of Crop Protection Chemistry at Ghent University, Belgium, and kept at 20 °C until extraction and analysis. 

### 2.3. Pesticides Extraction

Extraction and clean-up were performed using the QuEChERS method commonly used in the multi-residue analysis of food matrices. Each sample (approx. 50 g) was ground to powder using a household mill equipped with a stainless steel knife (Krups, Fleurus, Belgium). Each time the grinder was thoroughly washed to avoid cross-contamination between samples. Precisely 5 g of powder was weighed into a 50 mL Teflon capped centrifuge tube, 5 mL of Milli-Q water followed by 15 mL of acetonitrile was added, and the mixture was vigorously shaken for 1 min. A mixture of disodium hydrogen citrate sesquihydrate (0.75 g), trisodium citrate dihydrate (1.5 g), sodium chloride (1.5 g), and anhydrous magnesium sulphate (6 g) was added to the extract in the tube, which was agitated for 3 min at 300 rpm on a shaker (Edmund Bühler, Hechingen, Germany). The sample was centrifuged for 5 min at 10,000 rpm (Eppendorf, Leipzig, Germany) and the supernatant was collected. Samples of groundnuts, chili pepper, coffee, cocoa, and white pepper required clean-up to remove any organic acids, polar pigments, and other compounds that could interfere with the analysis. For clean-up, 8 mL of the supernatant was pipetted into a 15 mL d-SPE tube packed with 300 mg primary secondary amines (PSA), 900 mg MgSO_4_, and 150 mg octadecyl (C18). The content of the tube was then shaken for 1 min, centrifuged for 5 min at 3000 rpm, and the supernatant collected. For LC-MS/MS analysis, 1 mL of the supernatant was transferred into a 10 mL flask and the volume was made up to 10 mL with Milli-Q water. After mixing, 2 mL of the diluted solution was sampled into a screw cap autosampler vial for chromatography analysis. For GC-ECD analysis, 5 mL of supernatant was taken into a 10 mL flask and acetonitrile was evaporated (Heidolph Instruments, Schwabach, Germany) at 40 °C until dryness. Acetonitrile was replaced by 5 mL of hexane, and 2 mL of the extract was sampled into a crimp top autosampler vial for analysis. 

### 2.4. Sample Analysis

The selection of potential active ingredients to be screened was based on the list of registered agricultural pesticides authorized in Cameroon by the Ministry of Agriculture and Rural Development, commonly used on the sample crops [23]. For different chemical classes of pesticides, LC-MS/MS and GC-ECD were separately used. 

#### 2.4.1. Liquid Chromatography Tandem Mass Spectrometry

Eighty-one compounds were analyzed by LC-MS/MS (Table 1) following a protocol adapted from Houbraken et al. [24]. The equipment consisted of a Waters Acquity UPLC module coupled to a Waters Xevo TQD Tandem triple quadrupole mass spectrometer equipped with an electrospray ionization (ESI) interface (Waters, Zellik, Belgium). Separation was carried out through a HSS T3 column (100 mm × 2.1 mm, 1.8 µm) maintained at 40 °C. The injection volume was 10 μL, and mobile phase A consisted of 10 mM ammonium acetate solution in water, while mobile phase B was acetonitrile with 0.1% formic acid. The flow rate was set at 0.4 mL/min with a run time of 10 min. The separation started with an initial gradient of 98% mobile phase A for 0.25 min, followed by a linear gradient to 98% mobile phase B from 0.25 to 7 min, which was maintained for 1 min. Then, a linear gradient was used to 98% mobile phase A and the column was reconditioned for 1 min. Except for fludioxonil and 2,4-dichlorophenoxyacetic acid (2,4-D), which were analyzed in negative ion mode, analyses of all the other pesticides were performed in positive ion mode. The ESI capillary needle was maintained at +2 kV, the source temperature at 150 °C, the desolvation temperature at 600 °C, cone gas flow at 50 L/h, and desolvation gas flow at 1000 L/h. The analytes were monitored and quantified using multiple reaction monitoring (MRM). Optimization of the MS/MS conditions, identification of the parent and product ions, as well as the selection of the cone and collision voltages, were performed with direct infusion of their individual standard solutions prepared at 1 mg/mL in acetonitrile/water (10/90). Two different *m*/*z* transitions were selected for each analyte, one for quantification (QIT) and one for confirmation (CIT). The dwell time was calculated automatically. MassLynx 4.1 software (Waters) was used for the LC-MS/MS system control and data acquisition and analysis.

#### 2.4.2. Gas Liquid Chromatography with Electron Capture Detection

Eighteen halogenated compounds (Table 2) were analyzed using an Agilent 6890N Network gas chromatograph with an auto-sampler, coupled to an electron capture detector (Agilent Technologies, Diegem, Belgium). The protocol was adapted from Amulen et al. [25]. Separation was performed on a HP-5MS (5% phenyl methyl siloxane) capillary column (30 m × 0.25 mm, 0.25 μm). The operating conditions were as follows: The column was initially set at a temperature of 80 °C, then increased at a rate of 30 °C/min to 205 °C and held for 4 min. It was further increased at a rate of 20 °C/min to 290 °C and held constant for 8 min, followed by an increase at a rate of 50 °C/min to 325 °C. The temperature of the injector and detector were maintained at 280 °C and 300 °C, respectively. Helium was used as a carrier gas at a flow rate of 1.1 mL/min, and the injections were made in the split mode with a split ratio of 52.7:1. The Agilent GC ChemStation version Rev. A.10.02 software was used for system control and data acquisition and analysis.

### 2.5. Method Validation

Validation of the analysis was performed as recommended in Document No. SANTE/11945/2015 [26]. The validation parameters were linearity, limit of detection (LOD), limit of quantification (LOQ), accuracy, and precision. Eight replicates of samples obtained from organic agriculture markets in Belgium were spiked at 0.01 mg/kg with pesticide standards. The spiked samples were left for 1 h to allow pesticide absorption into samples before being subjected to the extraction, clean-up process, and analysis as described previously. The LOD together with the LOQ were calculated by multiplying the standard deviation of the detected pesticide concentrations from the replicates by 2.99 and 10, respectively. The accuracy (average recovery) was calculated by dividing the recovered concentrations by spiked concentration, and precision (relative standard deviation of within-laboratory reproducibility analyses, %RSD) was obtained by dividing the standard deviation by the average concentration. To determine linearity and calculate pesticide content in samples, five different concentrations of the pesticide standards stock solution (0.1, 0.05, 0.01, 0.005, 0.001 mg/L) were prepared by dilution with acetonitrile/water (10/90) to make a calibration curve. 

### 2.6. Data Analysis

Descriptive statistics depicting the frequencies of occurrence and the distribution of quantified pesticides in the analyzed agricultural products and the sampling locations were generated. The quantified pesticides were compared with the maximum residue limits (MRLs) of the European Union regulations. As the number of samples was not equal for all the food items, the contamination rate (the relative number of positive samples), the pesticide rate (relative number of pesticides quantified), as well as the above MRLs rate (relative number of samples above the MRLs values) for each food item were calculated. 

## 3. Results

### 3.1. Method Validation

Five attributes of the extraction and analysis methods were validated: Accuracy (percentage recovery), precision (%RSD), limit of detection (LOD), limit of quantification (LOQ), and linearity. The obtained recoveries data and validation parameters of the 2 analysis methods are presented in Supplementary Material Table S1. The linearity of analysis of 81 pesticides by LC-MS/MS ranged between 0.9993 and 0.9999 (Table 1). The recovery varied greatly among the pesticides, and for a given pesticide, among the food commodities, with the median between 11.5% and 227% for methsulfuron-methyl and spiroxamine, respectively. In general, high recoveries (>120) were mostly found in groundnuts, soybeans, beans, and cocoa, while low values (<70) were mostly obtained in maize, white pepper, Egusi seeds, and coffee. The %RSD showed consistent precision with only 30 out of 720 (4.1%) of the %RSD values above the 20% acceptable threshold. The LOD ranged between 0.0001 and 0.0161 mg/kg, and the LOQ between 0.0004 and 0.0537 mg/kg.

Analysis of 18 halogenated pesticides by GC-ECD showed linearity between 0.9987 and 0.9999 (Table 2). Large variations were also observed in recoveries, with median values that ranged between 87.8 and 170.8% for Aldrin and *o*,*p*′-DDT, respectively. In general, beans and cocoa showed high recovery values, while low values were found in groundnuts and chili pepper. The %RSD showed that the analyses were precise; only 3 out of 153 (1.9%) of the %RSD values were above the 20% limit. The LOD varied from 0.0004 to 0.0652 mg/kg, and the LOQ from 0.0012 to 0.2180 mg/kg.

### 3.2. Pesticide Residues in Food Samples

After validation of the QuEChERS method, 99 pesticide residues were screened in 72 samples of 12 agricultural products. The results of the quantified pesticides are available in Supplementary Material Table S2, and the pesticide distributions are summarized in Table 3. All of the 18 halogenated compounds analyzed by GC-ECD could be quantified in the samples, against 44 of the 81 compounds analyzed by LC-MS/MS. Residues of 62 pesticides were found in the samples: 39.7% were insecticides, 30.9% were fungicides, herbicides represented 16.2%, acaricides 8.8%, and nematicides 4.4%. Twelve banned compounds were found in the samples, among which there was 1 herbicide (alachlor), 3 insecticide/acaricides (malathion, α-Endosulfan, and β-Endosulfan), and 8 insecticides (β-HCH, aldrin, carbaryl, carbofuran, diazinon, dieldrin, heptachlor, and propoxur). 

The distribution of the pesticides by sampling location showed that 22 pesticides (34.4%) were found in all 6 locations, while 15 pesticides (24.2%) could be found at only one sampling site. The repartition based on food commodities showed that 12 compounds, representing 19.3% of the pesticides quantified in the samples, were found in all 12 food items; except for malathion, 11 of them are halogenated compounds. However, 17 compounds (27.7%) could be quantified in only 1 food commodity. Considering individual pesticides, malathion was the most distributed pesticide as it was found in 70 samples, followed by *p*,*p*′-DDT, which was quantified in 69 samples. Thirteen individual halogenated pesticides were found in above 40 samples. Fifteen pesticides were quantified in only 1 sample. Concentration-wise, the single pesticides with the highest quantified concentrations were malathion in kidney beans (5.5 mg/kg), hexachlorobenzene in kidney beans (3.1 mg/kg), chlorotoluron in soybeans (1.5 mg/kg), cypermethrin in kidney beans (0.9 mg/kg), captan in chili pepper (0.8 mg/kg), and methoxychlor in maize (0.8 mg/kg). In general, malathion was found at all 12 sampling locations, in almost all the 72 samples, in all 12 food commodities, and with the highest concentrations. Whereas isoproturon was found at only 1 sampling location, in a single black bean sample, and with the lowest concentration (0.0004 mg/kg). 

The distribution of quantified pesticides in the 12 food items (Table 4) demonstrates that all samples contained one or more of the 62 quantified pesticides. Chili pepper showed the highest relative number of positive samples, with a contamination rate of 23.2%, followed by white pepper (20.2%), kidney beans (17.3%), and soybeans (17.2%). White pepper, with a pesticide rate of 14.6%, contained the highest relative number of pesticides per sample, followed by cocoa (8.1%) and Bambara nuts (8.1%). 

In total, 21 pesticides (34.4%) were found above their existing European Union MRL values (Supplementary Material Table S2, Table 3). Malathion was found above its MRL in 49 samples, followed by aldrin (30 samples), hexachlorobenzene (25 samples), alachlor (22 samples), and β-HCH (21 samples). Except for Bambara nuts and Egusi seeds, which do not have established MRL values yet, all the food items had residues above the threshold limit fixed by the European Union. Samples with residues above MRLs represented 38% of all the positive analyses, and were found in all 6 sampling locations. Chili pepper showed the highest above MRL rate (6.4%), followed by kidney beans (5.5%), while the lowest rate (2.1%) was found in groundnuts. 

## 4. Discussion

According to the European Commission, if the recoveries of pesticides are consistently high or low in replicate tests, this outcome is acceptable [26]. The method appeared to be suitable for detecting almost all the targeted compounds in all the food items, and adjustments were performed for recoveries lower than 70% or higher than 120%. Consistently low recoveries were found with apolar compounds, in cleaned-up samples, and in samples with high fat content like groundnuts, Egusi seeds, and cocoa beans. This can be attributed to losses during the extraction and clean-up steps with d-SPE tubes in the modified QuEChERS method [27]. These observations agree with the findings of Mekonen et al. [2] on similar food items. Conversely, very high or very low recovery values were also consistently obtained for polar pesticides, and in some samples which did not require clean-up. In LC-MS/MS analysis, this may be due to the presence of matrix components that coelute with the compounds of interest and can interfere with the ionization process in the mass spectrometer, causing ionization suppression or enhancement; this phenomenon is called the matrix effect [28]. To further understand these recovery differences and ensure a thorough quantitative analysis, the matrix effect of these food matrices on the screened pesticides must be investigated.

In this study, pesticides were detected in all the samples. In a similar preliminary study of pesticide residues in fruits at the market level in Accra, Ghana, pesticide residues were also found in all the 320 samples of pawpaw, tomato, and apple [29]. All 42 samples of staple food items from the Jimma zone in Ethiopia also contained 1 or more pesticide [2]. Additionally, our results of the 62 quantified pesticides in the food items agree with the findings of Winter [30], who reported a total of 77 individual pesticides detected from market basket samples analyzed by the U.S. Food and Drug Administration in 2004 and 2005. The report of Manfo et al. [4], who showed that 56 pesticides containing 25 active substances were used by farmers of Djutitsa, located in our sampling region of Cameroon, can justify our results. Moreover, Mahob et al. [6] found that 35 different chemicals were marketed in Cameroon for use in cocoa, among which there were 4 herbicides, 11 fungicides, and 20 insecticides. The great majority of farmers (96.8%) apply pesticides on their farms, while fungicides were used most often (61.8%), followed by insecticides (38.2%). 

In a similar study on staple foods from Ethiopia, the main pesticides detected were DDT, endosulfan, cypermethrin, and permethrin at concentrations varying from 0.011 to 1.115 mg/kg [2]. In maize, cowpea, and millet samples from northern Cameroon, organochlorine and organophosphorus pesticides including β-endosulfan, lindane, pirimiphos-methyl, and malathion were detected more frequently [10]. Moreover, Sonchieu et al. [5] found that in cowpea samples from the markets of Ngaoundéré in northern Cameroon, residues of the organophosphorus pesticides dichlorvos, methyl-parathion, malathion, profenofos, diazinon, and chlorpyrifos were found in concentrations ranging from 0.02 to 5.4 mg/kg. The above results corroborate our findings of halogenated compounds as the most distributed pesticides in samples from the western highlands of Cameroon, and the pesticide concentrations we obtained are on par with all these studies. 

Our analyses revealed that 34.4% of the pesticides were found above their existing MRL values and in 38% of the positive analyses. Our results are comparable to the findings of Bempah and Donkor [29], who reported 32.8% of fruit samples with residues above MRLs, and Mekonen et al. [2], in which one-third of samples were above the MRLs. However, Sonchieu et al. [10] obtained higher values, with 75% of samples containing pesticide residues above MRLs. These disparities could be due to the single food item (cowpea) used in their study. 

We found 12 banned compounds in the samples, including DTT and its metabolites, as well as β-Hexachlorocyclohexane, a by-product of lindane. Similar results were reported in Ghana [29], in Ethiopia [2], and in samples from northern Cameroon [5,10,17]. This raises the concern of the presence of banned pesticides in foods in Africa, and particularly in Cameroon. This can result from environmental persistence of these pesticides, which could have returned into the food chain. However, in Cameroon, the availability of a huge stock of obsolete pesticides, coupled with their illegal use in agriculture, can justify the high distribution and concentration of banned pesticides in analyzed food samples. In 353 stores visited in the whole country, Tarla et al. [16] inventoried 210,047 kg and 309,521 L of obsolete pesticides, among which there was 4146 kg of persistent organic pollutants. Eight officially banned active ingredients were still being used in Cameroonian cocoa farms, and over 77% of farmers did not respect the official spray recommendations for chemicals [6]. Similarly, in Foumbot, one of our sampling locations, it was found that because of the absence of any formal training on pesticide application, and the lack of assistance from agricultural extension agents, farmers did not respect treatment frequencies [7]. Moreover, Sonchieu et al. [17] reported that the presence of high residues in northern Cameroon samples indicates the continuous use of banned pesticides in Cameroon acquired from black markets, because of their effectiveness and low price, to the detriment of consumer health. 

## 5. Conclusions

It is critical to understand the occurrence of agrochemical contaminants in foods for assessing the health risk and preserving consumer health. This study validated a multi-residue method, and used it to screen 99 pesticides in 12 agricultural products from the food basket of Cameroon using LC-MS/MS and GC-ECD. We found that samples from all 6 locations and of all 12 food items were contaminated with one or more of the 63 pesticides quantified, among which 12 banned compounds were found. Halogenated pesticides, especially malathion, were highly distributed among the samples. Chili pepper and white pepper were the most contaminated food items. Twenty one pesticides were found above their European Union MRL values and represented 38% of the positive samples. These results pave the way for estimating the potential health risks associated with exposure to these pesticides in Cameroon. They also represent scientific evidence to create awareness on the necessity of good pesticide monitoring in Cameroon. There is an urgent need to develop strategies for lowering pesticide residues in food, and actions to be taken by regulatory authorities to manage the countries obsolete pesticide stock and regulate agrochemical usage in the country.

## Figures and Tables

**Table 1:** Parameters of acquisition method and linearity results of LC-MS/MS analysis of 81 pesticides.

Sr. No.	Analyte	Retention Time (min)	Parent Ion (*m*/*z*)	Cone Voltage (eV)	Ionization Mode	Dwell Time (s)	Fragment Ion 1 (*m*/*z*)	Collision Energy 1 (eV)	Fragment Ion 2 (*m*/*z*)	Collision Energy 2 (eV)	Linearity
1	2,4-dichlorophenoxyacetic acid	3.51	160.7	50	-	0.071	88.9	20	124.9 *	18	0.9998
2	Acetamiprid	2.72	223	34	+	0.015	56.1	15	126 *	20	0.9998
3	Ametryn	3.11	228.1	32	+	0.013	68.1	36	186.1 *	18	0.9997
4	Atrazine	2.46	174	30	+	0.038	96 *	20	103.9	20	0.9995
5	Azoxystrobin	4.23	404	22	+	0.015	329	30	372 *	15	0.9998
6	Benalaxyl	4.96	326.1	20	+	0.064	91	34	148 *	20	0.9998
7	Bentazone	3.32	241.4	21	+	0.015	107.2	26	199.1 *	12	0.9994
8	Bitertanol	4.74	338.1	15	+	0.015	70.1 *	8	99.1	16	0.9998
9	Boscalid	4.40	342.9	35	+	0.013	139.9 *	20	307	20	0.9997
10	Butachlor	6.14	312.2	20	+	0.067	57.3	22	238.2 *	12	0.9998
11	Cadusafos	5.20	271.1	22	+	0.015	131	22	159 *	16	0.9997
12	Carbaryl	3.38	202	22	+	0.08	117	28	145 *	22	0.9998
13	Carbendazim	2.28	192.1	27	+	0.08	132.1	28	16.1 *	18	0.9998
14	Carbofuran	3.22	222.1	28	+	0.012	123 *	16	165.1	16	0.9989
15	Chlorpyrifos	6.31	349.9	30	+	0.037	97 *	32	198	20	0.9994
16	Chlorotoluron	3.36	213	20	+	0.03	72 *	20	140	30	0.9999
17	Cyanazine	3.09	241.1	35	+	0.03	96	25	214	17	0.9998
18	Cyflufenamid	5.73	413.2	30	+	0.052	203	35	295.1 *	15	0.9999
19	Cymoxanil	2.79	199	17	+	0.015	111	18	128 *	8	0.9999
20	Diazion	5.20	305	31	+	0.017	96	35	169 *	22	0.9998
21	Difenconazole	5.21	406	40	+	0.015	111.1	60	251.1 *	25	0.9993
22	Dimethoate	2.67	230.1	18	+	0.012	125	20	199 *	10	0.9987
23	Dimethomorph	4.02	388.1	35	+	0.013	165	30	300.9 *	20	0.9993
24	Diuron	3.56	233	28	+	0.012	46.3	14	72.1 *	18	0.9987
25	Epoxiconazole	4.33	330	28	+	0.03	101	50	121 *	22	0.9997
26	Ethoprophos	4.34	243.2	26	+	0.012	97	31	131 *	20	0.9989
27	Fenamiphos	4.30	304.1	30	+	0.012	202.1	36	217.1 *	24	0.9999
28	Fenbuconazole	4.68	337	32	+	0.012	70.1 *	20	125	36	0.9983
29	Fenoxycarb	4.74	302.1	22	+	0.03	88	20	116.1 *	11	0.9999
30	Fenpropimorph	3.44	304.2	50	+	0.015	57.2	30	147.2 *	28	0.9995
31	Fludioxonil	4.18	246.8	50	-	0.013	126 *	30	180	28	0.9997
32	Hexaconazole	4.69	314	16	+	0.013	70.1 *	34	159	22	0.9998
33	Hexythiazox	6.31	353	24	+	0.136	168.1	26	228.1 *	14	0.9996
34	Imazalil	3.02	297	34	+	0.02	69 *	22	159	22	0.9984
35	Imidacloprid	2.64	256.1	34	+	0.038	175.1 *	20	209.1	15	0.9997
36	Iprodione	4.68	330	15	+	0.015	244.7 *	16	288	15	0.9992
37	Isoproturon	3.50	207.3	34	+	0.03	46	16	72 *	16	0.9997
38	Kresoxim-methyl	5.01	314.1	18	+	0.017	116	12	206 *	7	0.9997
39	Linuron	4.10	249.1	31	+	0.015	159.9 *	18	181.8	16	0.9997
40	Malathion	4.55	331	20	+	0.013	99	24	127 *	12	0.9997
41	Metalaxyl	3.44	280.1	20	+	0.012	192.1	17	220.1 *	13	0.9986
42	Methiocarb	4.01	226	22	+	0.015	121	22	169 *	10	0.9996
43	Methomyl	2.40	163	20	+	0.017	88 *	10	106	10	0.9998
44	Metribuzin	3.10	215	35	+	0.012	89	20	131 *	18	0.9988
45	Metsulfuron methyl	3.15	382	22	+	0.02	167 *	16	198	22	0.9997
46	Monocrotophos	2.40	224.1	20	+	0.163	98.1	12	127.1 *	16	0.9996
47	Oxamyl	2.37	237	15	+	0.163	72 *	10	90	10	0.9998
48	Penconazole	4.67	284	28	+	0.052	70.1 *	16	159	34	0.9999
49	Pendimethanil	6.29	282.2	20	+	0.028	194	18	212.2 *	10	0.9992
50	Pirimicarb	2.54	239.1	28	+	0.017	72	28	182.1 *	15	0.9998
51	Pirimiphos-methyl	5.13	306.1	30	+	0.052	108.1 *	32	164.1	22	0.9999
52	Prochloraz	4.19	376	16	+	0.015	70.1 *	34	307.1	16	0.9994
53	Profenofos	6.68	372.9	36	+	0.017	127.9	40	302.6 *	20	0.9998
54	Propanil	3.93	217.9	34	+	0.015	127	22	161.9 *	16	0.9999
55	Propazine	3.95	230.2	34	+	0.03	146.1	24	188.1 *	18	0.9994
56	Propiconazole	4.81	342	40	+	0.017	69	22	159 *	34	0.9998
57	Propoxur	3.19	210	15	+	0.013	111 *	16	168	10	0.9997
58	Pyrachlostrobin	5.37	388.1	25	+	0.017	163	25	193.9 *	12	0.9995
59	Pyrazosulfuron-ethyl	4.07	415	22	+	0.012	82.9	45	182 *	20	0.9992
60	Pyrimethanil	3.39	200	45	+	0.015	82	24	107 *	24	0.9996
61	Simazine	3.08	202	34	+	0.03	96	22	124 *	16	0.9993
62	Spinosad A	4.12	732.6	50	+	0.013	98.1	59	142 *	31	0.9997
63	Spinosad D	4.39	746.5	45	+	0.013	98.1	53	142 *	31	0.9999
64	Spirodiclofen	7.00	411.1	25	+	0.108	71.2 *	13	313	13	0.9999
65	Spiroxamine	3.47	298	32	+	0.013	100	32	144 *	20	0.9990
66	Tebuconazole	4.55	308	40	+	0.015	70.1 *	22	125	40	0.9999
67	Tebufenozide	4.95	353.1	13	+	0.052	133	20	297.1 *	8	0.9997
68	Tebuthiuron	2.90	229	30	+	0.015	116	16	172 *	18	0.9997
69	Temephos	6.30	466.8	32	+	0.052	125 *	38	418.9	22	0.9988
70	Terbufos	6.11	289	12	+	0.017	57.2	22	103 *	8	0.9997
71	Terbutryn	3.51	242.1	34	+	0.015	91	28	186.1 *	20	0.9997
72	Terbutylazine	4.01	230	28	+	0.03	96	28	174 *	16	0.9998
73	Thiabendazole	2.36	202	45	+	0.013	131	30	175 *	25	0.9997
74	Thiacloprid	2.87	253	25	+	0.071	90.1	40	126 *	20	0.9998
75	Thifensulfuron-methyl	3.08	388	30	+	0.015	56	40	167 *	15	0.9997
76	Thimetoxam	2.49	292	22	+	0.038	132	22	211.2 *	12	0.9995
77	Thiodicarb	3.17	355	20	+	0.015	87.9 *	16	107.9	16	0.9998
78	Tiofanate-methyl	3.12	343	22	+	0.015	93	46	151	22	0.9997
79	Triadimenol	4.07	296.1	15	+	0.017	70.2 *	10	99.1	15	0.9999
80	Triazophos	4.65	314.1	25	+	0.012	118.9	35	161.9 *	18	0.9997
81	Trifloxystrobin	5.78	409	28	+	0.073	145	40	186 *	16	0.9994

* Represents the transition used for quantification (QIT). LC-MS/MS: liquid chromatography tandem mass spectrometry.

**Table 2:** Parameters and linearity results of GC-ECD analysis of 18 pesticides.

Sr. No.	Retention Time (min)	Analyte	Linearity
1	10.6	Heptaclor	0.9997
2	10.8	β-HCH	0.9996
3	11.4	Chlorothalonil	0.9992
4	12.05	Alachlor	0.9994
5	12.8	Aldrin	0.9993
6	13.5	Hexachlorobenzene	0.9999
7	13.6	Captan	0.9987
8	14.2	α-Endosulfan	0.9999
9	14.5	*p*,*p*′-DDE	0.9999
10	14.7	Dieldrin	0.9999
11	15.2	Endrin	0.9996
12	15.3	*p*,*p*′-DDD	0.9998
13	15.3	β-Endosulfan	0.9998
14	15.5	*o*,*p*′-DDT	0.9999
15	16.2	*p*,*p*′-DDT	0.9997
16	17.4	Bifenthrin	0.9999
17	17.6	Methoxychlor	0.9988
18	24.2	Cypermethrin	0.9999

HCH = Hexachlorocyclohexane, DEE = Dichlorodiphenyldichloroethylene, DDD = Dichlorodiphenyldichloroethane, DDT = Dichlorodiphenyltrichloroethane.

**Table 3:** Characteristics of the 62 pesticides analyzed by LC-MS/MS and GC-ECD (gas chromatography-electron capture detection) in 12 food items from the western highlands of Cameroon.

Sr. No.	Pesticide	Method	Application	Banned	Number of Positive Locations	Number of Positive Food Items	Number of Positive Samples	Lowest Value (mg/kg)	Highest Value (mg/kg)	Mean Value (mg/kg)	Median (mg/kg)	Number of Samples >MRLs
1	Acetamiprid	LC-MS/MS	Insecticide	No	5	4	6	0.0004	0.0400	0.0086	0.0025	0
2	Alachlor	GC-ECD	Herbicide	Yes	6	12	48	0.0026	0.6275	0.0610	0.0186	22
3	Aldrin	GC-ECD	Insecticide	Yes	6	12	64	0.0012	0.4646	0.0663	0.0224	30
4	Atrazine	LC-MS/MS	Herbicide	No	6	11	29	0.0006	0.0040	0.0018	0.0013	0
5	Azoxystrobin	LC-MS/MS	Fungicide	No	1	1	1	0.0011	0.0011	0.0011	0.0011	0
6	Benalaxyl	LC-MS/MS	Fungicide	No	2	1	2	0.0046	0.0222	0.0134	0.0134	0
7	Bentazon	LC-MS/MS	Herbicide	No	3	4	5	0.0020	0.0128	0.0066	0.0062	0
8	Bifenthrin	GC-ECD	Insecticide	No	2	3	4	0.0056	0.0310	0.0139	0.0096	0
9	Bitertanol	LC-MS/MS	Fungicide	No	4	3	4	0.0008	0.0093	0.0034	0.0017	0
10	Cadusafos	LC-MS/MS	Insecticide/Nematicide	No	4	1	4	0.0089	0.6285	0.1684	0.0182	3
11	Captan	GC-ECD	Fungicide	No	6	12	60	0.0097	0.8557	0.1467	0.0390	19
12	Carbaryl	LC-MS/MS	Insecticide	Yes	2	2	2	0.0297	0.0758	0.0528	0.0528	0
13	Carbendazim	LC-MS/MS	Fungicide	No	1	1	1	0.0014	0.0014	0.0014	0.0014	0
14	Carbofuran	LC-MS/MS	Insecticide	Yes	3	2	3	0.0006	0.0027	0.0015	0.0011	0
15	Chlorothalonil	GC-ECD	Fungicide	No	6	12	48	0.0039	0.0683	0.0123	0.0092	2
16	Chlorpyrifos	LC-MS/MS	Insecticide	No	3	5	6	0.0071	0.3667	0.1155	0.0720	3
17	Chlorotoluron	LC-MS/MS	Herbicide	No	6	7	11	0.0709	1.5508	0.2609	0.1083	10
18	Cypermethrin	GC-ECD	Insecticide	No	6	12	52	0.0014	0.9449	0.0694	0.0213	11
19	Diazinon	LC-MS/MS	Insecticide	Yes	1	1	1	0.0020	0.0020	0.0020	0.0020	0
20	Dieldrin	GC-ECD	Insecticide	Yes	6	10	44	0.0012	0.0604	0.0069	0.0029	5
21	Difenconazole	LC-MS/MS	Fungicide	No	6	3	11	0.0009	0.0021	0.0014	0.0013	0
22	Dimethomorph	LC-MS/MS	Fungicide	No	1	1	1	0.0007	0.0007	0.0007	0.0007	0
23	Endrin	GC-ECD	Insecticide	No	6	10	36	0.0012	0.0337	0.0050	0.0028	3
24	Epoxiconazole	LC-MS/MS	Fungicide	No	6	6	26	0.0004	0.0176	0.0032	0.0016	0
25	Fenamiphos	LC-MS/MS	Nematicide	No	1	1	1	0.0014	0.0014	0.0014	0.0014	0
26	Fenbuconazole	LC-MS/MS	Fungicide	No	3	2	3	0.0009	0.0015	0.0011	0.0009	0
27	Fenoxycarb	LC-MS/MS	Insecticide	No	1	1	1	0.0013	0.0013	0.0013	0.0013	0
28	Fenpropimorf	LC-MS/MS	Fungicide	No	4	3	7	0.0004	0.0013	0.0008	0.0005	0
29	Heptaclor	GC-ECD	Insecticide	Yes	6	7	16	0.0012	0.1236	0.0182	0.0021	2
30	Hexachlorobenzene	GC-ECD	Fungicide	No	6	12	57	0.0014	3.0895	0.1362	0.0219	25
31	Hexaconazole	LC-MS/MS	Fungicide	No	2	2	2	0.0025	0.0121	0.0073	0.0073	0
32	Imazalil	LC-MS/MS	Fungicide	No	1	1	1	0.0032	0.0032	0.0032	0.0032	0
33	Imidacloprid	LC-MS/MS	Insecticide	No	2	5	6	0.0008	0.0120	0.0045	0.0035	0
34	Isoproturon	LC-MS/MS	Herbicide	No	1	1	1	0.0004	0.0004	0.0004	0.0004	0
35	Linuron	LC-MS/MS	Herbicide	No	2	3	3	0.0024	0.1041	0.0367	0.0036	1
36	Malathion	LC-MS/MS	Insecticide/Acaricide	Yes	6	12	70	0.0073	5.5269	0.9546	0.3137	49
37	Metalaxyl	LC-MS/MS	Fungicide	Yes	5	7	9	0.0004	0.1736	0.0205	0.0007	1
38	Methiocarb	LC-MS/MS	Acaricide/Insecticide	No	3	2	4	0.0015	0.0061	0.0043	0.0048	6
39	Methoxychlor	GC-ECD	Insecticide	No	5	5	10	0.0160	0.8165	0.1821	0.0475	0
40	Methribuzin	LC-MS/MS	Herbicide	No	1	1	1	0.0037	0.0037	0.0037	0.0037	0
41	Monocrotophos	LC-MS/MS	Acaricide	No	3	3	3	0.0012	0.0079	0.0034	0.0012	0
42	*o*,*p*′-DDT	GC-ECD	Insecticide	No	6	10	51	0.0013	0.0156	0.0041	0.0027	0
43	*p*,*p*′-DDD	GC-ECD	Insecticide	No	6	12	42	0.0012	0.0241	0.0037	0.0022	0
44	*p*,*p*′-DDE	GC-ECD	Insecticide	No	6	12	44	0.0013	0.0276	0.0051	0.0024	0
45	*p*,*p*′-DDT	GC-ECD	Insecticide	No	6	12	69	0.0033	0.1466	0.0246	0.0146	7
46	Penconazole	LC-MS/MS	Fungicide	No	4	2	7	0.0062	0.0214	0.0116	0.0098	0
47	Pirimiphos-methyl	LC-MS/MS	Insecticide	No	6	11	48	0.0004	0.2735	0.0139	0.0030	6
48	Propazine	LC-MS/MS	Herbicide	No	1	1	1	0.0032	0.0032	0.0032	0.0032	0
49	Propiconazole	LC-MS/MS	Fungicide	No	2	2	3	0.0004	0.0032	0.0017	0.0016	0
50	Propoxur	LC-MS/MS	Insecticide	Yes	2	2	2	0.0006	0.0017	0.0011	0.0011	0
51	Pyrimethanil	LC-MS/MS	Fungicide	No	2	4	4	0.0062	0.0929	0.0372	0.0248	1
52	Simazine	LC-MS/MS	Herbicide	No	1	1	1	0.0005	0.0005	0.0005	0.0005	0
53	Tebuconazole	LC-MS/MS	Fungicide	No	1	1	1	0.0016	0.0016	0.0016	0.0016	0
54	Tebufenozide	LC-MS/MS	Insecticide	No	5	5	9	0.0004	0.0048	0.0014	0.0007	0
55	Terbuthryn	LC-MS/MS	Herbicide	No	1	1	1	0.0041	0.0041	0.0041	0.0041	0
56	Terbuthylazine	LC-MS/MS	Herbicide	No	6	11	41	0.0053	0.1878	0.0454	0.0275	8
57	Thiofanate-methyl	LC-MS/MS	Fungicide	No	3	2	3	0.0013	0.0180	0.0070	0.0017	0
58	Triazophos	LC-MS/MS	Acaricide/Nematicide	No	2	2	2	0.0013	0.0020	0.0016	0.0016	0
59	Trifloxystrobin	LC-MS/MS	Fungicide	No	1	1	1	0.0012	0.0012	0.0012	0.0012	0
60	α-Endosulfan	GC-ECD	Insecticide/Acaricide	Yes	6	12	63	0.0012	0.0415	0.0076	0.0049	0
61	β-Endosulfan	GC-ECD	Insecticide/Acaricide	Yes	1	1	1	0.0017	0.0017	0.0017	0.0017	0
62	β-HCH	GC-ECD	Insecticide	Yes	6	12	47	0.0012	0.1371	0.0153	0.0060	21

**Table 4:** Distribution of quantified pesticides in the 12 food items from the western highlands of Cameroon.

Sr. No.	Food Item	Number of Samples	Number of Analyses	Number of Quantifications	Contamination Rate (%)	Number of Pesticides Quantified	Pesticides Rate (%)	Number of Samples >MRLs	Above MRLs Rate (%)
1	Bambara nuts	3	297	44	14.8	24	8.1	NA	NA
2	Black beans	6	594	56	9.4	22	3.7	18	3.0
3	Chili pepper	6	594	138	23.2	35	5.9	38	6.4
4	Cocoa	2	198	27	13.6	16	8.1	5	2.5
5	Coffee	4	396	57	14.4	23	5.8	12	3.0
6	Cowpea	7	693	107	15.4	28	4.0	32	4.6
7	Egusi seeds	7	693	103	14.9	24	3.5	NA	NA
8	Groundnuts	8	792	84	10.6	20	2.5	17	2.1
9	Kidney beans	10	990	171	17.3	31	3.1	54	5.5
10	Maize	11	1089	176	16.2	31	2.8	31	2.8
11	Soybeans	6	594	102	17.2	32	5.4	19	3.2
12	White pepper	2	198	40	20.2	29	14.6	9	4.5

NA: Not Applicable, because of no existing MRLs.

## Supplementary Materials

The following are available online at http://www.mdpi.com/2304-8158/7/11/184/s1; Table S1: Recovery data and validation parameters of analysis of 99 pesticides in 12 food items from western highlands of Cameroon by LC-MS/MS and GC-ECD; Table S2: Analysis of 99 pesticide residues in 72 samples of 12 agricultural products from western highlands of Cameroon by LC-MS/MS and GC-ECD.
