# Supplementary material for: Evaluation of 99 Pesticide Residues in Major Agricultural Products from the Western Highlands Zone of Cameroon Using QuEChERS Method Extraction and LC-MS/MS and GC-ECD Analyses
Source: Foods. 2018 Nov 7;7(11):184. doi: 10.3390/foods7110184 (PMC6262625; doi:10.3390/foods7110184)
Supplement: Supplementary file 1 [file foods-07-00184-s001.pdf]

Table S1. Recovery data and validation parameters of analysis of 99 pesticides in 12 food items from western highlands of Cameroon by LC-MS/MS and GC-ECD

Compound	Food item	Recovery (%)	Standard Deviation	RSD (%)	LOD (mg/kg)	LOQ (mg/kg)
**LC/MS-MS**						
2,4-D	Groundnuts					
2,4-D	Soybeans	153.7	0.0016	10.6	0.0049	0.016
2,4-D	Beans	140.6	0.0015	10.3	0.0043	0.015
2,4-D	Chili pepper					
2,4-D	Egusi seeds	135.9	0.0013	9.2	0.0037	0.013
2,4-D	Coffee					
2,4-D	Cocoa					
2,4-D	Maize	204.0	0.0034	16.6	0.0101	0.034
2,4-D	White pepper					
Acetamiprid	Groundnuts	147.2	0.0017	11.5	0.0050	0.017
Acetamiprid	Soybeans	82.7	0.0005	6.3	0.0016	0.005
Acetamiprid	Beans	138.3	0.0006	4.1	0.0017	0.006
Acetamiprid	Chili pepper	82.4	0.0003	4.1	0.0010	0.003
Acetamiprid	Egusi seeds	130.5	0.0007	5.0	0.0020	0.007
Acetamiprid	Coffee	109.0	0.0002	1.4	0.0005	0.002
Acetamiprid	Cocoa	82.3	0.0010	12.3	0.0030	0.010
Acetamiprid	Maize	123.9	0.0004	3.2	0.0012	0.004
Acetamiprid	White pepper	57.7	0.0003	5.0	0.0009	0.003
Amethryn	Groundnuts	134.7	0.0004	3.0	0.0012	0.004
Amethryn	Soybeans	122.2	0.0005	4.2	0.0015	0.005
Amethryn	Beans	128.6	0.0011	8.3	0.0032	0.011
Amethryn	Chili pepper	120.9	0.0008	6.5	0.0023	0.008
Amethryn	Egusi seeds	12.6	0.0001	4.3	0.0002	0.001
Amethryn	Coffee	105.2	0.0004	3.9	0.0012	0.004
Amethryn	Cocoa	96.8	0.0005	4.8	0.0014	0.005
Amethryn	Maize	51.5	0.0002	3.9	0.0006	0.002
Amethryn	White pepper	42.5	0.0002	4.5	0.0006	0.002
Atrazine	Groundnuts	118.2	0.0016	13.5	0.0048	0.016
Atrazine	Soybeans	86.7	0.0009	10.8	0.0028	0.009
Atrazine	Beans	120.2	0.0005	3.9	0.0014	0.005
Atrazine	Chili pepper	107.9	0.0010	9.7	0.0031	0.010
Atrazine	Egusi seeds	102.9	0.0009	9.0	0.0028	0.009
Atrazine	Coffee	86.7	0.0008	9.6	0.0025	0.008
Atrazine	Cocoa	92.7	0.0009	9.2	0.0026	0.009
Atrazine	Maize	83.2	0.0006	7.6	0.0019	0.006
Atrazine	White pepper	96.4	0.0006	6.7	0.0019	0.006
Azoxystrobin	Groundnuts	184.8	0.0008	4.5	0.0025	0.008
Azoxystrobin	Soybeans	175.4	0.0009	4.9	0.0026	0.009
Azoxystrobin	Beans	172.8	0.0011	6.1	0.0032	0.011

Compound	Food item	Recovery (%)	Standard Deviation	RSD (%)	LOD (mg/kg)	LOQ (mg/kg)
Azoxystrobin	Chili pepper	80.5	0.0005	6.2	0.0015	0.005
Azoxystrobin	Egusi seeds	73.1	0.0005	6.8	0.0015	0.005
Azoxystrobin	Coffee	150.9	0.0007	4.5	0.0020	0.007
Azoxystrobin	Cocoa	171.1	0.0008	4.4	0.0023	0.008
Azoxystrobin	Maize	158.8	0.0011	7.1	0.0034	0.011
Azoxystrobin	White pepper					
Benalaxyl	Groundnuts	151.8	0.0006	3.6	0.0017	0.006
Benalaxyl	Soybeans	142.3	0.0010	7.0	0.0030	0.010
Benalaxyl	Beans	144.8	0.0009	6.3	0.0027	0.009
Benalaxyl	Chili pepper	133.3	0.0005	3.4	0.0014	0.005
Benalaxyl	Egusi seeds	26.3	0.0003	11.1	0.0009	0.003
Benalaxyl	Coffee	116.9	0.0002	1.4	0.0005	0.002
Benalaxyl	Cocoa	141.8	0.0004	2.7	0.0011	0.004
Benalaxyl	Maize	75.0	0.0002	2.8	0.0006	0.002
Benalaxyl	White pepper	14.0	0.0001	7.3	0.0003	0.001
Bentazon	Groundnuts	68.7	0.0004	5.9	0.0012	0.004
Bentazon	Soybeans	109.7	0.0016	14.7	0.0048	0.016
Bentazon	Beans	169.4	0.0020	11.9	0.0060	0.020
Bentazon	Chili pepper	118.5	0.0002	1.9	0.0007	0.002
Bentazon	Egusi seeds	57.6	0.0007	12.9	0.0022	0.007
Bentazon	Coffee	90.9	0.0014	14.9	0.0040	0.014
Bentazon	Cocoa	48.3	0.0013	26.5	0.0038	0.013
Bentazon	Maize	353.7	0.0041	11.5	0.0121	0.041
Bentazon	White pepper					
Bitertanol	Groundnuts	182.9	0.0026	14.1	0.0077	0.026
Bitertanol	Soybeans	196.1	0.0041	21.1	0.0124	0.041
Bitertanol	Beans	199.9	0.0043	21.7	0.0130	0.043
Bitertanol	Chili pepper	90.5	0.0015	17.0	0.0046	0.015
Bitertanol	Egusi seeds					
Bitertanol	Coffee	127.2	0.0021	16.8	0.0064	0.021
Bitertanol	Cocoa	148.7	0.0019	12.6	0.0056	0.019
Bitertanol	Maize	65.1	0.0016	24.4	0.0048	0.016
Bitertanol	White pepper					
Boscalid	Groundnuts	82.7	0.0009	10.6	0.0026	0.009
Boscalid	Soybeans	99.1	0.0012	12.6	0.0037	0.012
Boscalid	Beans	94.1	0.0010	11.0	0.0031	0.010
Boscalid	Chili pepper	11.2	0.0002	15.1	0.0005	0.002
Boscalid	Egusi seeds	17.8	0.0003	15.6	0.0008	0.003
Boscalid	Coffee	94.3	0.0004	4.6	0.0013	0.004
Boscalid	Cocoa	81.1	0.0006	7.3	0.0018	0.006
Boscalid	Maize	62.5	0.0004	6.2	0.0012	0.004
Boscalid	White pepper	11.0	0.0002	18.0	0.0006	0.002

Compound	Food item	Recovery (%)	Standard Deviation	RSD (%)	LOD (mg/kg)	LOQ (mg/kg)
Butachlor	Groundnuts	69.5	0.0004	5.9	0.0012	0.004
Butachlor	Soybeans	48.5	0.0011	22.1	0.0032	0.011
Butachlor	Beans	43.5	0.0003	8.0	0.0010	0.003
Butachlor	Chili pepper	47.4	0.0011	23.1	0.0033	0.011
Butachlor	Egusi seeds	3.1	0.0001	16.7	0.0002	0.001
Butachlor	Coffee	29.9	0.0002	7.2	0.0006	0.002
Butachlor	Cocoa	56.4	0.0001	2.2	0.0004	0.001
Butachlor	Maize	8.9	0.0002	21.3	0.0006	0.002
Butachlor	White pepper	3.3	0.0000	12.9	0.0001	0.000
Cadusafos	Groundnuts	116.6	0.0005	4.6	0.0016	0.005
Cadusafos	Soybeans	115.7	0.0008	7.3	0.0025	0.008
Cadusafos	Beans	118.7	0.0006	5.0	0.0018	0.006
Cadusafos	Chili pepper	53.7	0.0004	8.3	0.0013	0.004
Cadusafos	Egusi seeds	15.5	0.0001	6.8	0.0003	0.001
Cadusafos	Coffee	91.6	0.0004	4.4	0.0012	0.004
Cadusafos	Cocoa	104.8	0.0003	2.6	0.0008	0.003
Cadusafos	Maize	52.5	0.0004	7.8	0.0012	0.004
Cadusafos	White pepper	15.8	0.0002	10.4	0.0005	0.002
Carbaryl	Groundnuts	75.0	0.0003	4.0	0.0009	0.003
Carbaryl	Soybeans	82.0	0.0004	5.0	0.0012	0.004
Carbaryl	Beans	89.9	0.0006	6.8	0.0018	0.006
Carbaryl	Chili pepper	77.9	0.0008	9.9	0.0023	0.008
Carbaryl	Egusi seeds	52.1	0.0009	16.8	0.0026	0.009
Carbaryl	Coffee	70.4	0.0006	8.0	0.0017	0.006
Carbaryl	Cocoa	77.3	0.0008	10.5	0.0024	0.008
Carbaryl	Maize	96.4	0.0008	8.2	0.0024	0.008
Carbaryl	White pepper	31.7	0.0006	18.4	0.0017	0.006
Carbendazim	Groundnuts	127.0	0.0011	8.5	0.0032	0.011
Carbendazim	Soybeans	96.7	0.0007	7.4	0.0021	0.007
Carbendazim	Beans	108.8	0.0006	5.6	0.0018	0.006
Carbendazim	Chili pepper	85.1	0.0009	10.7	0.0027	0.009
Carbendazim	Egusi seeds	69.4	0.0004	5.2	0.0011	0.004
Carbendazim	Coffee	115.7	0.0002	1.6	0.0005	0.002
Carbendazim	Cocoa	54.3	0.0001	2.4	0.0004	0.001
Carbendazim	Maize	117.8	0.0004	3.7	0.0013	0.004
Carbendazim	White pepper	85.2	0.0007	7.8	0.0020	0.007
Carbofuran	Groundnuts	121.1	0.0007	5.8	0.0021	0.007
Carbofuran	Soybeans	100.2	0.0009	9.4	0.0028	0.009
Carbofuran	Beans	106.5	0.0009	8.4	0.0027	0.009
Carbofuran	Chili pepper	109.3	0.0002	2.2	0.0007	0.002
Carbofuran	Egusi seeds	86.7	0.0002	2.5	0.0007	0.002
Carbofuran	Coffee	97.6	0.0001	1.0	0.0003	0.001

Compound	Food item	Recovery (%)	Standard Deviation	RSD (%)	LOD (mg/kg)	LOQ (mg/kg)
Carbofuran	Cocoa	103.3	0.0003	2.5	0.0008	0.003
Carbofuran	Maize	112.8	0.0001	0.7	0.0002	0.001
Carbofuran	White pepper	87.0	0.0003	3.5	0.0009	0.003
Chlorotoluron	Groundnuts	149.5	0.0008	5.4	0.0024	0.008
Chlorotoluron	Soybeans	115.7	0.0010	8.6	0.0030	0.010
Chlorotoluron	Beans	132.8	0.0006	4.4	0.0017	0.006
Chlorotoluron	Chili pepper	119.7	0.0004	3.0	0.0011	0.004
Chlorotoluron	Egusi seeds	79.3	0.0003	4.3	0.0010	0.003
Chlorotoluron	Coffee	127.6	0.0006	4.9	0.0019	0.006
Chlorotoluron	Cocoa	128.2	0.0007	5.1	0.0020	0.007
Chlorotoluron	Maize	136.5	0.0004	2.7	0.0011	0.004
Chlorotoluron	White pepper	21.5	0.0002	11.1	0.0007	0.002
Chlorpyrifos	Groundnuts	40.1	0.0004	10.3	0.0012	0.004
Chlorpyrifos	Soybeans	66.2	0.0014	20.9	0.0041	0.014
Chlorpyrifos	Beans	24.8	0.0004	17.9	0.0013	0.004
Chlorpyrifos	Chili pepper	126.1	0.0025	19.8	0.0075	0.025
Chlorpyrifos	Egusi seeds					
Chlorpyrifos	Coffee	17.4	0.0003	19.9	0.0010	0.003
Chlorpyrifos	Cocoa	34.7	0.0003	7.9	0.0008	0.003
Chlorpyrifos	Maize	7.2	0.0001	14.0	0.0003	0.001
Chlorpyrifos	White pepper	16.3	0.0001	6.1	0.0003	0.001
Cyanazine	Groundnuts	155.3	0.0007	4.3	0.0020	0.007
Cyanazine	Soybeans	170.5	0.0013	7.7	0.0039	0.013
Cyanazine	Beans	173.4	0.0012	7.1	0.0037	0.012
Cyanazine	Chili pepper	123.0	0.0005	3.9	0.0015	0.005
Cyanazine	Egusi seeds	74.9	0.0005	6.8	0.0015	0.005
Cyanazine	Coffee	153.5	0.0004	2.5	0.0012	0.004
Cyanazine	Cocoa	125.6	0.0005	3.8	0.0014	0.005
Cyanazine	Maize	134.6	0.0006	4.4	0.0018	0.006
Cyanazine	White pepper	40.0	0.0003	7.3	0.0009	0.003
Cyflufenamid	Groundnuts	96.9	0.0002	2.2	0.0006	0.002
Cyflufenamid	Soybeans	64.8	0.0014	22.3	0.0043	0.014
Cyflufenamid	Beans	54.3	0.0007	12.1	0.0020	0.007
Cyflufenamid	Chili pepper	73.4	0.0009	12.3	0.0027	0.009
Cyflufenamid	Egusi seeds	3.2	0.0000	11.7	0.0001	0.000
Cyflufenamid	Coffee	38.9	0.0002	5.2	0.0006	0.002
Cyflufenamid	Cocoa	85.2	0.0009	10.1	0.0026	0.009
Cyflufenamid	Maize	16.0	0.0002	11.0	0.0005	0.002
Cyflufenamid	White pepper					
Cymoxanil	Groundnuts					
Cymoxanil	Soybeans	128.7	0.0007	5.1	0.0020	0.007
Cymoxanil	Beans	140.0	0.0004	3.0	0.0012	0.004

Compound	Food item	Recovery (%)	Standard Deviation	RSD (%)	LOD (mg/kg)	LOQ (mg/kg)
Cymoxanil	Chili pepper	70.1	0.0008	12.0	0.0025	0.008
Cymoxanil	Egusi seeds	117.7	0.0009	7.4	0.0026	0.009
Cymoxanil	Coffee	63.4	0.0010	15.8	0.0030	0.010
Cymoxanil	Cocoa	99.2	0.0006	6.4	0.0019	0.006
Cymoxanil	Maize	116.0	0.0007	6.3	0.0022	0.007
Cymoxanil	White pepper					
Diazianon	Groundnuts	161.0	0.0003	2.0	0.0010	0.003
Diazianon	Soybeans	135.8	0.0027	19.8	0.0080	0.027
Diazianon	Beans	140.7	0.0011	8.1	0.0034	0.011
Diazianon	Chili pepper	105.9	0.0005	4.3	0.0013	0.005
Diazianon	Egusi seeds	15.7	0.0001	6.3	0.0003	0.001
Diazianon	Coffee	116.9	0.0002	1.5	0.0005	0.002
Diazianon	Cocoa	146.6	0.0006	4.3	0.0019	0.006
Diazianon	Maize	54.9	0.0004	7.7	0.0013	0.004
Diazianon	White pepper	15.1	0.0003	16.8	0.0008	0.003
Difenconazole	Groundnuts	128.0	0.0003	2.7	0.0010	0.003
Difenconazole	Soybeans	125.9	0.0020	15.9	0.0060	0.020
Difenconazole	Beans	105.5	0.0007	6.4	0.0020	0.007
Difenconazole	Chili pepper	71.0	0.0004	5.6	0.0012	0.004
Difenconazole	Egusi seeds	18.2	0.0003	14.5	0.0008	0.003
Difenconazole	Coffee	85.1	0.0017	20.0	0.0051	0.017
Difenconazole	Cocoa	83.6	0.0004	4.5	0.0011	0.004
Difenconazole	Maize	25.4	0.0001	4.5	0.0003	0.001
Difenconazole	White pepper	18.0	0.0001	6.5	0.0004	0.001
Dimethoate	Groundnuts	125.6	0.0004	3.0	0.0011	0.004
Dimethoate	Soybeans	67.8	0.0010	14.6	0.0030	0.010
Dimethoate	Beans	107.9	0.0010	9.2	0.0030	0.010
Dimethoate	Chili pepper	61.0	0.0003	4.4	0.0008	0.003
Dimethoate	Egusi seeds	105.9	0.0001	1.2	0.0004	0.001
Dimethoate	Coffee	83.7	0.0003	3.2	0.0008	0.003
Dimethoate	Cocoa	72.7	0.0002	3.4	0.0007	0.002
Dimethoate	Maize	112.2	0.0005	4.0	0.0013	0.005
Dimethoate	White pepper	60.9	0.0007	11.5	0.0021	0.007
Dimethomorph	Groundnuts	161.5	0.0006	3.5	0.0017	0.006
Dimethomorph	Soybeans	205.9	0.0010	4.8	0.0030	0.010
Dimethomorph	Beans	180.9	0.0019	10.5	0.0057	0.019
Dimethomorph	Chili pepper	126.5	0.0008	6.7	0.0025	0.008
Dimethomorph	Egusi seeds	41.3	0.0001	1.3	0.0002	0.001
Dimethomorph	Coffee	130.2	0.0003	2.6	0.0010	0.003
Dimethomorph	Cocoa	149.5	0.0008	5.6	0.0025	0.008
Dimethomorph	Maize	124.5	0.0014	11.3	0.0042	0.014
Dimethomorph	White pepper	15.9	0.0001	8.0	0.0004	0.001

Compound	Food item	Recovery (%)	Standard Deviation	RSD (%)	LOD (mg/kg)	LOQ (mg/kg)
Diuron	Groundnuts	107.3	0.0004	3.7	0.0012	0.004
Diuron	Soybeans	117.8	0.0014	12.0	0.0042	0.014
Diuron	Beans	116.9	0.0015	12.9	0.0045	0.015
Diuron	Chili pepper	57.9	0.0006	10.9	0.0019	0.006
Diuron	Egusi seeds	40.4	0.0005	13.2	0.0016	0.005
Diuron	Coffee	90.7	0.0003	3.0	0.0008	0.003
Diuron	Cocoa	99.9	0.0007	6.9	0.0021	0.007
Diuron	Maize	107.8	0.0016	14.7	0.0047	0.016
Diuron	White pepper					
Epoxiconazole	Groundnuts	160.2	0.0002	1.3	0.0006	0.002
Epoxiconazole	Soybeans	193.8	0.0029	15.0	0.0087	0.029
Epoxiconazole	Beans	147.6	0.0004	2.6	0.0012	0.004
Epoxiconazole	Chili pepper	37.9	0.0003	7.1	0.0008	0.003
Epoxiconazole	Egusi seeds	95.5	0.0012	12.3	0.0035	0.012
Epoxiconazole	Coffee	166.4	0.0005	2.8	0.0014	0.005
Epoxiconazole	Cocoa	130.7	0.0005	3.5	0.0014	0.005
Epoxiconazole	Maize	46.4	0.0003	5.6	0.0008	0.003
Epoxiconazole	White pepper	13.9	0.0003	18.1	0.0008	0.003
Ethoprophos	Groundnuts	103.1	0.0004	4.3	0.0013	0.004
Ethoprophos	Soybeans	113.0	0.0011	10.1	0.0034	0.011
Ethoprophos	Beans	108.1	0.0008	7.0	0.0022	0.008
Ethoprophos	Chili pepper					
Ethoprophos	Egusi seeds					
Ethoprophos	Coffee	84.4	0.0002	2.9	0.0007	0.002
Ethoprophos	Cocoa	104.7	0.0004	4.1	0.0013	0.004
Ethoprophos	Maize	80.7	0.0006	8.0	0.0019	0.006
Ethoprophos	White pepper					
Fenamiphos	Groundnuts	40.1	0.0006	13.9	0.0017	0.006
Fenamiphos	Soybeans	101.9	0.0020	19.8	0.0060	0.020
Fenamiphos	Beans	88.2	0.0017	19.4	0.0051	0.017
Fenamiphos	Chili pepper					
Fenamiphos	Egusi seeds					
Fenamiphos	Coffee	44.3	0.0007	15.0	0.0020	0.007
Fenamiphos	Cocoa	68.6	0.0008	11.0	0.0022	0.008
Fenamiphos	Maize					
Fenamiphos	White pepper					
Fenbuconazole	Groundnuts	122.3	0.0013	10.8	0.0040	0.013
Fenbuconazole	Soybeans	115.1	0.0041	35.5	0.0122	0.041
Fenbuconazole	Beans	102.8	0.0009	9.1	0.0028	0.009
Fenbuconazole	Chili pepper	31.6	0.0004	13.5	0.0013	0.004
Fenbuconazole	Egusi seeds					
Fenbuconazole	Coffee	68.5	0.0002	2.2	0.0005	0.002

Compound	Food item	Recovery (%)	Standard Deviation	RSD (%)	LOD (mg/kg)	LOQ (mg/kg)
Fenbuconazole	Cocoa	97.4	0.0003	3.6	0.0010	0.003
Fenbuconazole	Maize	12.8	0.0001	9.9	0.0004	0.001
Fenbuconazole	White pepper					
Fenoxycarb	Groundnuts	116.3	0.0005	4.5	0.0016	0.005
Fenoxycarb	Soybeans	98.5	0.0012	11.8	0.0035	0.012
Fenoxycarb	Beans	95.2	0.0004	4.0	0.0011	0.004
Fenoxycarb	Chili pepper	14.0	0.0001	6.7	0.0003	0.001
Fenoxycarb	Egusi seeds	8.0	0.0001	14.0	0.0003	0.001
Fenoxycarb	Coffee	60.0	0.0004	6.8	0.0012	0.004
Fenoxycarb	Cocoa	107.0	0.0002	1.5	0.0005	0.002
Fenoxycarb	Maize	30.5	0.0001	2.3	0.0002	0.001
Fenoxycarb	White pepper					
Fenpropimorf	Groundnuts	176.6	0.0006	3.5	0.0019	0.006
Fenpropimorf	Soybeans	220.6	0.0002	1.0	0.0007	0.002
Fenpropimorf	Beans	228.8	0.0015	6.7	0.0046	0.015
Fenpropimorf	Chili pepper	156.8	0.0016	10.3	0.0049	0.016
Fenpropimorf	Egusi seeds	97.9	0.0002	2.1	0.0006	0.002
Fenpropimorf	Coffee	130.2	0.0012	8.9	0.0035	0.012
Fenpropimorf	Cocoa	159.7	0.0006	3.7	0.0017	0.006
Fenpropimorf	Maize	118.8	0.0003	2.6	0.0009	0.003
Fenpropimorf	White pepper	101.7	0.0001	1.3	0.0004	0.001
Hexaconazole	Groundnuts	131.6	0.0010	7.4	0.0029	0.010
Hexaconazole	Soybeans	135.7	0.0015	11.2	0.0045	0.015
Hexaconazole	Beans	133.3	0.0017	13.1	0.0052	0.017
Hexaconazole	Chili pepper	71.6	0.0011	15.2	0.0033	0.011
Hexaconazole	Egusi seeds	13.5	0.0001	7.9	0.0003	0.001
Hexaconazole	Coffee	99.3	0.0007	7.1	0.0021	0.007
Hexaconazole	Cocoa	107.3	0.0005	4.6	0.0015	0.005
Hexaconazole	Maize	49.1	0.0006	11.6	0.0017	0.006
Hexaconazole	White pepper	197.2	0.0008	4.1	0.0024	0.008
Hexythiazox	Groundnuts	42.5	0.0002	5.5	0.0007	0.002
Hexythiazox	Soybeans	45.5	0.0007	15.9	0.0022	0.007
Hexythiazox	Beans	39.9	0.0006	14.6	0.0017	0.006
Hexythiazox	Chili pepper	67.1	0.0006	9.1	0.0018	0.006
Hexythiazox	Egusi seeds	6.7	0.0001	7.7	0.0002	0.001
Hexythiazox	Coffee	16.0	0.0002	15.5	0.0007	0.002
Hexythiazox	Cocoa	34.7	0.0003	7.9	0.0008	0.003
Hexythiazox	Maize	8.4	0.0002	19.1	0.0005	0.002
Hexythiazox	White pepper					
Imazalil	Groundnuts	15.0	0.0002	11.2	0.0005	0.002
Imazalil	Soybeans	17.7	0.0002	9.6	0.0005	0.002
Imazalil	Beans	18.7	0.0002	8.3	0.0005	0.002

Compound	Food item	Recovery (%)	Standard Deviation	RSD (%)	LOD (mg/kg)	LOQ (mg/kg)
Imazalil	Chili pepper	14.1	0.0001	6.3	0.0003	0.001
Imazalil	Egusi seeds					
Imazalil	Coffee	8.9	0.0001	10.5	0.0003	0.001
Imazalil	Cocoa	11.2	0.0001	5.5	0.0002	0.001
Imazalil	Maize	6.7	0.0001	10.0	0.0002	0.001
Imazalil	White pepper	3.2	0.0000	12.3	0.0001	0.000
Imidacloprid	Groundnuts	153.5	0.0015	10.0	0.0046	0.015
Imidacloprid	Soybeans	70.2	0.0011	15.4	0.0032	0.011
Imidacloprid	Beans	151.9	0.0019	12.7	0.0058	0.019
Imidacloprid	Chili pepper	71.4	0.0007	10.1	0.0022	0.007
Imidacloprid	Egusi seeds	136.6	0.0017	12.1	0.0049	0.017
Imidacloprid	Coffee	269.0	0.0013	5.0	0.0040	0.013
Imidacloprid	Cocoa	116.4	0.0018	15.4	0.0053	0.018
Imidacloprid	Maize	113.9	0.0006	5.3	0.0018	0.006
Imidacloprid	White pepper	77.1	0.0008	10.2	0.0023	0.008
Iprodione	Groundnuts	93.7	0.0014	14.5	0.0041	0.014
Iprodione	Soybeans	214.1	0.0039	18.4	0.0118	0.039
Iprodione	Beans	214.1	0.0041	19.4	0.0124	0.041
Iprodione	Chili pepper	157.0	0.0031	20.0	0.0094	0.031
Iprodione	Egusi seeds	123.8	0.0031	25.3	0.0094	0.031
Iprodione	Coffee	157.3	0.0037	23.4	0.0110	0.037
Iprodione	Cocoa	223.3	0.0043	19.4	0.0130	0.043
Iprodione	Maize	172.1	0.0032	18.6	0.0096	0.032
Iprodione	White pepper					
Isoproturon	Groundnuts	130.7	0.0003	2.5	0.0010	0.003
Isoproturon	Soybeans	101.0	0.0009	9.3	0.0028	0.009
Isoproturon	Beans	111.0	0.0005	4.8	0.0016	0.005
Isoproturon	Chili pepper	108.1	0.0008	7.1	0.0023	0.008
Isoproturon	Egusi seeds	54.3	0.0001	2.3	0.0004	0.001
Isoproturon	Coffee	104.9	0.0005	4.9	0.0015	0.005
Isoproturon	Cocoa	108.1	0.0005	4.4	0.0014	0.005
Isoproturon	Maize	115.4	0.0008	7.1	0.0025	0.008
Isoproturon	White pepper	39.0	0.0001	2.6	0.0003	0.001
Kresoxim_methyl	Groundnuts	67.6	0.0005	7.4	0.0015	0.005
Kresoxim_methyl	Soybeans	63.4	0.0013	19.8	0.0037	0.013
Kresoxim_methyl	Beans	30.8	0.0004	12.5	0.0012	0.004
Kresoxim_methyl	Chili pepper	21.9	0.0003	12.1	0.0008	0.003
Kresoxim_methyl	Egusi seeds					
Kresoxim_methyl	Coffee					
Kresoxim_methyl	Cocoa	62.9	0.0004	5.7	0.0011	0.004
Kresoxim_methyl	Maize					
Kresoxim_methyl	White pepper					

Compound	Food item	Recovery (%)	Standard Deviation	RSD (%)	LOD (mg/kg)	LOQ (mg/kg)
Linuron	Groundnuts	124.7	0.0009	7.1	0.0027	0.009
Linuron	Soybeans	124.5	0.0014	11.2	0.0042	0.014
Linuron	Beans	140.0	0.0016	11.3	0.0047	0.016
Linuron	Chili pepper	78.7	0.0003	3.6	0.0008	0.003
Linuron	Egusi seeds	42.5	0.0004	9.7	0.0012	0.004
Linuron	Coffee	104.1	0.0012	11.0	0.0034	0.012
Linuron	Cocoa	111.7	0.0012	10.3	0.0035	0.012
Linuron	Maize	111.8	0.0015	13.2	0.0044	0.015
Linuron	White pepper	7.5	0.0001	19.9	0.0004	0.001
Malathion	Groundnuts	235.8	0.0021	9.1	0.0064	0.021
Malathion	Soybeans	150.7	0.0011	7.3	0.0033	0.011
Malathion	Beans	128.1	0.0008	6.0	0.0023	0.008
Malathion	Chili pepper	81.8	0.0003	4.0	0.0010	0.003
Malathion	Egusi seeds	41.2	0.0001	3.2	0.0004	0.001
Malathion	Coffee	118.2	0.0009	7.4	0.0026	0.009
Malathion	Cocoa	125.7	0.0008	6.7	0.0025	0.008
Malathion	Maize	236.4	0.0031	13.3	0.0094	0.031
Malathion	White pepper	14.9	0.0001	9.0	0.0004	0.001
Metalaxyl	Groundnuts	148.3	0.0009	6.0	0.0027	0.009
Metalaxyl	Soybeans	116.1	0.0011	9.7	0.0034	0.011
Metalaxyl	Beans	141.2	0.0018	12.7	0.0054	0.018
Metalaxyl	Chili pepper	117.1	0.0003	2.5	0.0009	0.003
Metalaxyl	Egusi seeds	110.3	0.0003	2.6	0.0009	0.003
Metalaxyl	Coffee	121.9	0.0004	3.5	0.0013	0.004
Metalaxyl	Cocoa	308.6	0.0004	1.3	0.0012	0.004
Metalaxyl	Maize	153.4	0.0032	20.6	0.0095	0.032
Metalaxyl	White pepper	41.6	0.0006	13.6	0.0017	0.006
Methiocarb	Groundnuts	87.5	0.0010	11.3	0.0029	0.010
Methiocarb	Soybeans	115.6	0.0009	7.7	0.0027	0.009
Methiocarb	Beans	117.5	0.0003	2.2	0.0008	0.003
Methiocarb	Chili pepper	64.4	0.0004	6.1	0.0012	0.004
Methiocarb	Egusi seeds	28.5	0.0002	5.6	0.0005	0.002
Methiocarb	Coffee	77.6	0.0008	9.7	0.0022	0.008
Methiocarb	Cocoa	105.3	0.0004	3.5	0.0011	0.004
Methiocarb	Maize	79.0	0.0005	5.7	0.0014	0.005
Methiocarb	White pepper					
Methomyl	Groundnuts	186.1	0.0003	1.6	0.0009	0.003
Methomyl	Soybeans	132.1	0.0005	4.1	0.0016	0.005
Methomyl	Beans	126.0	0.0003	2.6	0.0010	0.003
Methomyl	Chili pepper	73.2	0.0007	10.1	0.0022	0.007
Methomyl	Egusi seeds	153.1	0.0002	1.4	0.0006	0.002
Methomyl	Coffee	41.1	0.0001	3.0	0.0004	0.001

Compound	Food item	Recovery (%)	Standard Deviation	RSD (%)	LOD (mg/kg)	LOQ (mg/kg)
Methomyl	Cocoa	42.7	0.0003	6.5	0.0008	0.003
Methomyl	Maize	105.8	0.0005	4.4	0.0014	0.005
Methomyl	White pepper	55.1	0.0002	3.1	0.0005	0.002
Methsulfuron_methyl	Groundnuts	5.3	0.0001	9.9	0.0002	0.001
Methsulfuron_methyl	Soybeans	27.4	0.0002	6.5	0.0005	0.002
Methsulfuron_methyl	Beans	24.9	0.0002	9.0	0.0007	0.002
Methsulfuron_methyl	Chili pepper	5.4	0.0001	12.0	0.0002	0.001
Methsulfuron_methyl	Egusi seeds	16.4	0.0000	2.4	0.0001	0.000
Methsulfuron_methyl	Coffee					
Methsulfuron_methyl	Cocoa	6.6	0.0001	10.5	0.0002	0.001
Methsulfuron_methyl	Maize	18.8	0.0001	5.4	0.0003	0.001
Methsulfuron_methyl	White pepper	3.4	0.0000	8.5	0.0001	0.000
Metribuzin	Groundnuts	114.0	0.0017	14.6	0.0050	0.017
Metribuzin	Soybeans	72.7	0.0011	14.6	0.0032	0.011
Metribuzin	Beans	148.0	0.0033	22.5	0.0099	0.033
Metribuzin	Chili pepper	196.8	0.0024	12.3	0.0072	0.024
Metribuzin	Egusi seeds	24.3	0.0003	12.9	0.0009	0.003
Metribuzin	Coffee	74.0	0.0006	7.7	0.0017	0.006
Metribuzin	Cocoa	76.1	0.0010	12.7	0.0029	0.010
Metribuzin	Maize	111.2	0.0010	9.2	0.0031	0.010
Metribuzin	White pepper					
Monocrotophos	Groundnuts	91.6	0.0004	4.7	0.0013	0.004
Monocrotophos	Soybeans	65.8	0.0010	15.9	0.0031	0.010
Monocrotophos	Beans	56.7	0.0008	14.9	0.0025	0.008
Monocrotophos	Chili pepper	49.4	0.0003	6.7	0.0010	0.003
Monocrotophos	Egusi seeds	74.0	0.0004	5.0	0.0011	0.004
Monocrotophos	Coffee	25.7	0.0002	9.4	0.0007	0.002
Monocrotophos	Cocoa	11.3	0.0003	23.7	0.0008	0.003
Monocrotophos	Maize	36.1	0.0004	11.8	0.0013	0.004
Monocrotophos	White pepper	52.1	0.0006	11.3	0.0018	0.006
Oxamyl	Groundnuts	104.8	0.0022	20.8	0.0065	0.022
Oxamyl	Soybeans	131.1	0.0002	1.5	0.0006	0.002
Oxamyl	Beans	47.0	0.0002	4.6	0.0006	0.002
Oxamyl	Chili pepper	80.6	0.0008	10.1	0.0024	0.008
Oxamyl	Egusi seeds	134.5	0.0008	6.2	0.0025	0.008
Oxamyl	Coffee	23.0	0.0002	7.8	0.0005	0.002
Oxamyl	Cocoa	39.6	0.0003	6.7	0.0008	0.003
Oxamyl	Maize	112.3	0.0003	3.0	0.0010	0.003
Oxamyl	White pepper	70.5	0.0004	6.3	0.0013	0.004
Penconazole	Groundnuts	139.0	0.0004	2.8	0.0011	0.004
Penconazole	Soybeans	130.6	0.0013	9.9	0.0039	0.013
Penconazole	Beans	133.1	0.0003	2.2	0.0009	0.003

Compound	Food item	Recovery (%)	Standard Deviation	RSD (%)	LOD (mg/kg)	LOQ (mg/kg)
Penconazole	Chili pepper	60.8	0.0003	4.8	0.0009	0.003
Penconazole	Egusi seeds	10.4	0.0001	8.4	0.0003	0.001
Penconazole	Coffee	109.8	0.0004	4.0	0.0013	0.004
Penconazole	Cocoa	109.8	0.0006	5.7	0.0019	0.006
Penconazole	Maize	40.6	0.0002	5.5	0.0007	0.002
Penconazole	White pepper	10.7	0.0001	10.4	0.0003	0.001
Pendimethanil	Groundnuts	37.8	0.0010	27.5	0.0031	0.010
Pendimethanil	Soybeans	45.6	0.0010	21.6	0.0029	0.010
Pendimethanil	Beans	30.6	0.0007	23.2	0.0021	0.007
Pendimethanil	Chili pepper	48.0	0.0013	27.2	0.0039	0.013
Pendimethanil	Egusi seeds	28.2	0.0006	20.9	0.0018	0.006
Pendimethanil	Coffee	11.0	0.0001	4.7	0.0002	0.001
Pendimethanil	Cocoa	31.0	0.0008	27.1	0.0025	0.008
Pendimethanil	Maize					
Pendimethanil	White pepper					
Pirimicarb	Groundnuts	142.4	0.0006	4.3	0.0018	0.006
Pirimicarb	Soybeans	119.0	0.0004	3.4	0.0012	0.004
Pirimicarb	Beans	129.8	0.0001	1.1	0.0004	0.001
Pirimicarb	Chili pepper	109.2	0.0002	1.8	0.0006	0.002
Pirimicarb	Egusi seeds	102.9	0.0006	5.6	0.0017	0.006
Pirimicarb	Coffee	93.5	0.0004	4.0	0.0011	0.004
Pirimicarb	Cocoa	99.6	0.0003	3.0	0.0009	0.003
Pirimicarb	Maize	139.9	0.0004	3.0	0.0013	0.004
Pirimicarb	White pepper	105.1	0.0005	4.5	0.0014	0.005
Pirimiphos-methyl	Groundnuts	280.4	0.0005	1.9	0.0016	0.005
Pirimiphos-methyl	Soybeans	111.3	0.0035	31.8	0.0106	0.035
Pirimiphos-methyl	Beans	88.7	0.0006	7.1	0.0019	0.006
Pirimiphos-methyl	Chili pepper	92.0	0.0006	6.1	0.0017	0.006
Pirimiphos-methyl	Egusi seeds	7.4	0.0001	11.1	0.0002	0.001
Pirimiphos-methyl	Coffee	55.2	0.0002	3.9	0.0007	0.002
Pirimiphos-methyl	Cocoa	106.4	0.0009	8.0	0.0026	0.009
Pirimiphos-methyl	Maize	257.6	0.0023	9.0	0.0070	0.023
Pirimiphos-methyl	White pepper	2.6	0.0000	15.2	0.0001	0.000
Prochloraz	Groundnuts	119.1	0.0006	4.9	0.0018	0.006
Prochloraz	Soybeans	138.5	0.0009	6.2	0.0026	0.009
Prochloraz	Beans	120.5	0.0004	3.1	0.0011	0.004
Prochloraz	Chili pepper	75.2	0.0009	12.6	0.0028	0.009
Prochloraz	Egusi seeds	18.8	0.0001	3.8	0.0002	0.001
Prochloraz	Coffee	83.7	0.0011	13.1	0.0033	0.011
Prochloraz	Cocoa	81.5	0.0003	4.1	0.0010	0.003
Prochloraz	Maize	24.8	0.0001	2.7	0.0002	0.001
Prochloraz	White pepper					

Compound	Food item	Recovery (%)	Standard Deviation	RSD (%)	LOD (mg/kg)	LOQ (mg/kg)
Profenofos	Groundnuts	35.6	0.0004	12.5	0.0013	0.004
Profenofos	Soybeans	34.3	0.0004	12.2	0.0012	0.004
Profenofos	Beans	32.1	0.0004	13.0	0.0012	0.004
Profenofos	Chili pepper	37.1	0.0003	7.9	0.0009	0.003
Profenofos	Egusi seeds	6.6	0.0000	5.2	0.0001	0.000
Profenofos	Coffee	18.8	0.0002	10.8	0.0006	0.002
Profenofos	Cocoa	32.7	0.0004	13.1	0.0013	0.004
Profenofos	Maize	8.4	0.0001	14.0	0.0004	0.001
Profenofos	White pepper	7.0	0.0001	14.2	0.0003	0.001
Propanil	Groundnuts	143.7	0.0019	13.5	0.0058	0.019
Propanil	Soybeans	51.2	0.0003	6.1	0.0009	0.003
Propanil	Beans	191.2	0.0011	5.6	0.0032	0.011
Propanil	Chili pepper	84.6	0.0006	7.0	0.0018	0.006
Propanil	Egusi seeds	81.5	0.0002	2.2	0.0005	0.002
Propanil	Coffee	81.5	0.0004	5.5	0.0013	0.004
Propanil	Cocoa	127.8	0.0011	8.2	0.0031	0.011
Propanil	Maize	109.9	0.0006	5.3	0.0017	0.006
Propanil	White pepper	42.5	0.0009	21.4	0.0027	0.009
Propazine	Groundnuts	162.2	0.0007	4.1	0.0020	0.007
Propazine	Soybeans	145.7	0.0016	10.8	0.0047	0.016
Propazine	Beans	146.3	0.0009	6.4	0.0028	0.009
Propazine	Chili pepper	133.3	0.0009	6.8	0.0027	0.009
Propazine	Egusi seeds					
Propazine	Coffee	136.9	0.0005	3.6	0.0015	0.005
Propazine	Cocoa	121.1	0.0004	3.2	0.0011	0.004
Propazine	Maize	34.7	0.0003	7.2	0.0008	0.003
Propazine	White pepper	31.6	0.0001	4.1	0.0004	0.001
Propiconazole	Groundnuts	140.1	0.0004	2.9	0.0012	0.004
Propiconazole	Soybeans	123.3	0.0011	8.7	0.0032	0.011
Propiconazole	Beans	137.3	0.0008	5.8	0.0024	0.008
Propiconazole	Chili pepper	96.2	0.0009	9.4	0.0027	0.009
Propiconazole	Egusi seeds	13.5	0.0002	16.2	0.0007	0.002
Propiconazole	Coffee	99.4	0.0004	4.5	0.0013	0.004
Propiconazole	Cocoa	109.1	0.0004	3.2	0.0011	0.004
Propiconazole	Maize	37.8	0.0003	8.2	0.0009	0.003
Propiconazole	White pepper					
Propoxur	Groundnuts	118.8	0.0001	0.5	0.0002	0.001
Propoxur	Soybeans	106.7	0.0006	5.2	0.0016	0.006
Propoxur	Beans	112.0	0.0012	10.8	0.0036	0.012
Propoxur	Chili pepper	104.0	0.0007	7.0	0.0022	0.007
Propoxur	Egusi seeds	87.8	0.0003	3.8	0.0010	0.003
Propoxur	Coffee	94.7	0.0007	7.2	0.0020	0.007

Compound	Food item	Recovery (%)	Standard Deviation	RSD (%)	LOD (mg/kg)	LOQ (mg/kg)
Propoxur	Cocoa	102.6	0.0008	7.7	0.0024	0.008
Propoxur	Maize	116.3	0.0006	5.2	0.0018	0.006
Propoxur	White pepper	54.3	0.0001	2.3	0.0004	0.001
Pyraclostrobine	Groundnuts	78.7	0.0003	3.7	0.0009	0.003
Pyraclostrobine	Soybeans	49.9	0.0007	14.4	0.0022	0.007
Pyraclostrobine	Beans	54.2	0.0007	13.7	0.0022	0.007
Pyraclostrobine	Chili pepper	73.7	0.0004	5.0	0.0011	0.004
Pyraclostrobine	Egusi seeds					
Pyraclostrobine	Coffee	53.1	0.0005	10.0	0.0016	0.005
Pyraclostrobine	Cocoa	73.0	0.0007	9.3	0.0020	0.007
Pyraclostrobine	Maize	13.5	0.0002	11.3	0.0005	0.002
Pyraclostrobine	White pepper					
Pyrazosulfuron_ethyl	Groundnuts	59.9	0.0009	14.7	0.0026	0.009
Pyrazosulfuron_ethyl	Soybeans	248.9	0.0027	10.6	0.0079	0.027
Pyrazosulfuron_ethyl	Beans	171.1	0.0007	3.8	0.0020	0.007
Pyrazosulfuron_ethyl	Chili pepper	49.1	0.0008	16.8	0.0025	0.008
Pyrazosulfuron_ethyl	Egusi seeds	124.7	0.0013	10.8	0.0040	0.013
Pyrazosulfuron_ethyl	Coffee	29.4	0.0003	8.9	0.0008	0.003
Pyrazosulfuron_ethyl	Cocoa	56.1	0.0008	14.0	0.0023	0.008
Pyrazosulfuron_ethyl	Maize	136.7	0.0015	10.8	0.0044	0.015
Pyrazosulfuron_ethyl	White pepper	10.1	0.0002	17.8	0.0005	0.002
Pyrimethanil	Groundnuts	134.6	0.0009	6.5	0.0026	0.009
Pyrimethanil	Soybeans	182.1	0.0035	19.4	0.0106	0.035
Pyrimethanil	Beans	227.0	0.0010	4.4	0.0030	0.010
Pyrimethanil	Chili pepper	83.3	0.0009	11.0	0.0027	0.009
Pyrimethanil	Egusi seeds					
Pyrimethanil	Coffee	100.7	0.0005	5.0	0.0015	0.005
Pyrimethanil	Cocoa	281.3	0.0054	19.1	0.0161	0.054
Pyrimethanil	Maize	22.7	0.0004	18.8	0.0013	0.004
Pyrimethanil	White pepper					
Simazine	Groundnuts	147.2	0.0005	3.7	0.0016	0.005
Simazine	Soybeans	131.3	0.0008	5.9	0.0023	0.008
Simazine	Beans	139.3	0.0008	5.5	0.0023	0.008
Simazine	Chili pepper	119.5	0.0010	8.3	0.0030	0.010
Simazine	Egusi seeds	55.7	0.0002	4.2	0.0007	0.002
Simazine	Coffee	118.9	0.0001	0.9	0.0003	0.001
Simazine	Cocoa	134.6	0.0002	1.2	0.0005	0.002
Simazine	Maize	109.7	0.0007	6.4	0.0021	0.007
Simazine	White pepper	55.1	0.0003	6.3	0.0010	0.003
Spinosad_A	Groundnuts	107.6	0.0009	8.2	0.0026	0.009
Spinosad_A	Soybeans	155.5	0.0007	4.5	0.0021	0.007
Spinosad_A	Beans	126.3	0.0010	8.2	0.0031	0.010

Compound	Food item	Recovery (%)	Standard Deviation	RSD (%)	LOD (mg/kg)	LOQ (mg/kg)
Spinosad_A	Chili pepper	94.5	0.0017	18.4	0.0052	0.017
Spinosad_A	Egusi seeds	59.7	0.0001	1.2	0.0002	0.001
Spinosad_A	Coffee	75.1	0.0001	1.4	0.0003	0.001
Spinosad_A	Cocoa	89.2	0.0006	6.6	0.0017	0.006
Spinosad_A	Maize	76.5	0.0003	4.3	0.0010	0.003
Spinosad_A	White pepper	56.1	0.0000	0.4	0.0001	0.000
Spinosad_D	Groundnuts	106.5	0.0011	10.5	0.0033	0.011
Spinosad_D	Soybeans	222.3	0.0026	11.5	0.0076	0.026
Spinosad_D	Beans	142.3	0.0012	8.5	0.0036	0.012
Spinosad_D	Chili pepper	81.3	0.0019	23.7	0.0058	0.019
Spinosad_D	Egusi seeds	54.6	0.0002	3.8	0.0006	0.002
Spinosad_D	Coffee	69.9	0.0004	6.4	0.0013	0.004
Spinosad_D	Cocoa	86.6	0.0007	8.4	0.0022	0.007
Spinosad_D	Maize	68.5	0.0003	3.9	0.0008	0.003
Spinosad_D	White pepper	50.7	0.0001	1.6	0.0002	0.001
Spirodiclofen	Groundnuts	41.8	0.0006	15.0	0.0019	0.006
Spirodiclofen	Soybeans	58.5	0.0007	11.5	0.0020	0.007
Spirodiclofen	Beans	60.0	0.0009	14.4	0.0026	0.009
Spirodiclofen	Chili pepper	179.9	0.0014	7.7	0.0042	0.014
Spirodiclofen	Egusi seeds					
Spirodiclofen	Coffee	7.8	0.0002	27.9	0.0007	0.002
Spirodiclofen	Cocoa	26.9	0.0004	15.4	0.0012	0.004
Spirodiclofen	Maize					
Spirodiclofen	White pepper					
Spiroxamine	Groundnuts	227.0	0.0006	2.6	0.0018	0.006
Spiroxamine	Soybeans	280.9	0.0006	2.0	0.0017	0.006
Spiroxamine	Beans	298.2	0.0013	4.5	0.0040	0.013
Spiroxamine	Chili pepper	235.8	0.0023	9.8	0.0069	0.023
Spiroxamine	Egusi seeds	173.7	0.0003	1.5	0.0008	0.003
Spiroxamine	Coffee	184.2	0.0016	8.4	0.0046	0.016
Spiroxamine	Cocoa	227.8	0.0009	4.0	0.0027	0.009
Spiroxamine	Maize	207.4	0.0004	2.1	0.0013	0.004
Spiroxamine	White pepper	171.3	0.0002	1.1	0.0006	0.002
Tebuconazole	Groundnuts	171.2	0.0008	4.8	0.0025	0.008
Tebuconazole	Soybeans	165.6	0.0016	9.6	0.0048	0.016
Tebuconazole	Beans	156.7	0.0007	4.7	0.0022	0.007
Tebuconazole	Chili pepper	133.6	0.0002	1.6	0.0006	0.002
Tebuconazole	Egusi seeds	15.6	0.0002	14.5	0.0007	0.002
Tebuconazole	Coffee	147.4	0.0011	7.6	0.0033	0.011
Tebuconazole	Cocoa	139.3	0.0005	3.4	0.0014	0.005
Tebuconazole	Maize	75.1	0.0015	20.6	0.0046	0.015
Tebuconazole	White pepper	37.6	0.0004	10.4	0.0012	0.004

Compound	Food item	Recovery (%)	Standard Deviation	RSD (%)	LOD (mg/kg)	LOQ (mg/kg)
Tebufenozide	Groundnuts	225.9	0.0021	9.1	0.0061	0.021
Tebufenozide	Soybeans	208.7	0.0010	4.8	0.0030	0.010
Tebufenozide	Beans	200.9	0.0006	2.8	0.0017	0.006
Tebufenozide	Chili pepper	149.3	0.0008	5.1	0.0023	0.008
Tebufenozide	Egusi seeds	72.2	0.0007	10.2	0.0022	0.007
Tebufenozide	Coffee	158.7	0.0006	3.6	0.0017	0.006
Tebufenozide	Cocoa	194.3	0.0012	6.1	0.0036	0.012
Tebufenozide	Maize	105.8	0.0006	5.7	0.0018	0.006
Tebufenozide	White pepper	5.5	0.0001	16.4	0.0003	0.001
Tebuthiuron	Groundnuts	143.1	0.0005	3.3	0.0014	0.005
Tebuthiuron	Soybeans	126.2	0.0001	1.0	0.0004	0.001
Tebuthiuron	Beans	118.9	0.0005	4.5	0.0016	0.005
Tebuthiuron	Chili pepper	113.6	0.0005	4.5	0.0015	0.005
Tebuthiuron	Egusi seeds	117.8	0.0004	3.6	0.0013	0.004
Tebuthiuron	Coffee	115.4	0.0006	5.2	0.0018	0.006
Tebuthiuron	Cocoa	110.0	0.0007	6.3	0.0021	0.007
Tebuthiuron	Maize	129.5	0.0005	3.5	0.0014	0.005
Tebuthiuron	White pepper	39.5	0.0003	6.4	0.0008	0.003
Temephos	Groundnuts	30.0	0.0002	7.8	0.0007	0.002
Temephos	Soybeans	42.5	0.0007	16.9	0.0021	0.007
Temephos	Beans					
Temephos	Chili pepper	238.2	0.0023	9.6	0.0068	0.023
Temephos	Egusi seeds					
Temephos	Coffee					
Temephos	Cocoa					
Temephos	Maize					
Temephos	White pepper					
Terbufos	Groundnuts	89.9	0.0007	7.4	0.0020	0.007
Terbufos	Soybeans	51.5	0.0010	19.3	0.0030	0.010
Terbufos	Beans	18.2	0.0002	9.6	0.0005	0.002
Terbufos	Chili pepper	133.0	0.0007	4.9	0.0020	0.007
Terbufos	Egusi seeds					
Terbufos	Coffee					
Terbufos	Cocoa	59.5	0.0012	20.1	0.0036	0.012
Terbufos	Maize					
Terbufos	White pepper					
Terbuthryn	Groundnuts	126.5	0.0006	4.7	0.0018	0.006
Terbuthryn	Soybeans	102.9	0.0017	16.1	0.0049	0.017
Terbuthryn	Beans	119.3	0.0010	8.8	0.0031	0.010
Terbuthryn	Chili pepper	125.0	0.0003	2.7	0.0010	0.003
Terbuthryn	Egusi seeds	3.4	0.0001	19.7	0.0002	0.001
Terbuthryn	Coffee	89.3	0.0004	4.0	0.0011	0.004

Compound	Food item	Recovery (%)	Standard Deviation	RSD (%)	LOD (mg/kg)	LOQ (mg/kg)
Terbuthryn	Cocoa	73.7	0.0003	4.3	0.0010	0.003
Terbuthryn	Maize	16.7	0.0001	8.1	0.0004	0.001
Terbuthryn	White pepper	14.8	0.0002	10.3	0.0005	0.002
Terbuthylazine	Groundnuts	123.1	0.0002	1.8	0.0007	0.002
Terbuthylazine	Soybeans	146.6	0.0029	19.5	0.0085	0.029
Terbuthylazine	Beans	47.5	0.0004	7.7	0.0011	0.004
Terbuthylazine	Chili pepper	290.0	0.0049	16.9	0.0147	0.049
Terbuthylazine	Egusi seeds					
Terbuthylazine	Coffee	88.6	0.0010	11.6	0.0031	0.010
Terbuthylazine	Cocoa	127.6	0.0005	3.6	0.0014	0.005
Terbuthylazine	Maize	37.3	0.0006	16.4	0.0018	0.006
Terbuthylazine	White pepper					
Thiabendazole	Groundnuts	108.9	0.0007	6.5	0.0021	0.007
Thiabendazole	Soybeans	86.6	0.0004	4.7	0.0012	0.004
Thiabendazole	Beans	83.9	0.0007	8.6	0.0022	0.007
Thiabendazole	Chili pepper	60.0	0.0006	9.2	0.0017	0.006
Thiabendazole	Egusi seeds	20.4	0.0001	7.0	0.0004	0.001
Thiabendazole	Coffee	26.3	0.0001	5.1	0.0004	0.001
Thiabendazole	Cocoa	38.2	0.0004	9.5	0.0011	0.004
Thiabendazole	Maize	89.0	0.0005	5.4	0.0014	0.005
Thiabendazole	White pepper	44.1	0.0004	8.6	0.0011	0.004
Thiacloprid	Groundnuts	126.1	0.0004	2.9	0.0011	0.004
Thiacloprid	Soybeans	113.8	0.0026	22.9	0.0078	0.026
Thiacloprid	Beans	124.7	0.0003	2.3	0.0008	0.003
Thiacloprid	Chili pepper	72.2	7.89E-05	1.1	0.0002	0.001
Thiacloprid	Egusi seeds	105.4	0.0006	5.7	0.0018	0.006
Thiacloprid	Coffee	106.9	0.0004	3.4	0.0011	0.004
Thiacloprid	Cocoa	93.3	0.0008	8.8	0.0024	0.008
Thiacloprid	Maize	108.8	0.0004	3.3	0.0011	0.004
Thiacloprid	White pepper	13.1	0.0001	7.8	0.0003	0.001
Thiametoxam	Groundnuts	99.5	0.0004	4.0	0.0012	0.004
Thiametoxam	Soybeans	59.6	0.0002	3.1	0.0005	0.002
Thiametoxam	Beans	69.1	0.0011	16.2	0.0033	0.011
Thiametoxam	Chili pepper	32.0	0.0001	3.9	0.0004	0.001
Thiametoxam	Egusi seeds	92.3	0.0004	3.9	0.0011	0.004
Thiametoxam	Coffee	74.8	0.0003	4.0	0.0009	0.003
Thiametoxam	Cocoa	29.3	0.0006	19.3	0.0017	0.006
Thiametoxam	Maize	60.2	0.0008	12.5	0.0023	0.008
Thiametoxam	White pepper	49.7	0.0003	5.6	0.0008	0.003
Thifensulfuron	Groundnuts					
Thifensulfuron	Soybeans	303.5	0.0008	2.6	0.0024	0.008
Thifensulfuron	Beans	182.5	0.0008	4.4	0.0024	0.008

Compound	Food item	Recovery (%)	Standard Deviation	RSD (%)	LOD (mg/kg)	LOQ (mg/kg)
Thifensulfuron	Chili pepper	109.3	0.0145	132.9	0.0434	0.145
Thifensulfuron	Egusi seeds	149.4	0.0006	4.0	0.0018	0.006
Thifensulfuron	Coffee					
Thifensulfuron	Cocoa					
Thifensulfuron	Maize	159.4	0.0009	5.9	0.0028	0.009
Thifensulfuron	White pepper	389.7	0.0017	4.5	0.0052	0.017
Thiodicarb	Groundnuts					
Thiodicarb	Soybeans					
Thiodicarb	Beans					
Thiodicarb	Chili pepper	49.2	0.0003	5.7	0.0008	0.003
Thiodicarb	Egusi seeds					
Thiodicarb	Coffee	125.8	0.0007	5.3	0.0020	0.007
Thiodicarb	Cocoa	94.3	0.0005	5.6	0.0016	0.005
Thiodicarb	Maize					
Thiodicarb	White pepper	108.7	0.0008	7.6	0.0025	0.008
Thiofanate_methyl	Groundnuts					
Thiofanate_methyl	Soybeans	152.1	0.0012	7.9	0.0036	0.012
Thiofanate_methyl	Beans	161.8	0.0011	6.6	0.0032	0.011
Thiofanate_methyl	Chili pepper	86.7	0.0007	8.1	0.0021	0.007
Thiofanate_methyl	Egusi seeds					
Thiofanate_methyl	Coffee					
Thiofanate_methyl	Cocoa	117.8	0.0010	8.3	0.0029	0.010
Thiofanate_methyl	Maize	61.4	0.0006	10.4	0.0019	0.006
Thiofanate_methyl	White pepper	395.3	0.0019	4.7	0.0056	0.019
Triademinol	Groundnuts					
Triademinol	Soybeans	9.5	0.0000	1.6	0.0000	0.000
Triademinol	Beans	13.3	0.0000	3.4	0.0001	0.000
Triademinol	Chili pepper					
Triademinol	Egusi seeds					
Triademinol	Coffee	17.1	0.0001	8.2	0.0004	0.001
Triademinol	Cocoa					
Triademinol	Maize					
Triademinol	White pepper					
Triazophos	Groundnuts	160.0	0.0009	5.7	0.0027	0.009
Triazophos	Soybeans	137.7	0.0009	6.9	0.0028	0.009
Triazophos	Beans	150.3	0.0010	6.8	0.0031	0.010
Triazophos	Chili pepper	102.1	0.0010	9.3	0.0029	0.010
Triazophos	Egusi seeds	20.5	0.0002	9.7	0.0006	0.002
Triazophos	Coffee	117.1	0.0004	3.8	0.0013	0.004
Triazophos	Cocoa	152.1	0.0005	3.3	0.0015	0.005
Triazophos	Maize	76.4	0.0008	10.3	0.0024	0.008
Triazophos	White pepper	111.8	0.0017	15.4	0.0052	0.017

Compound	Food item	Recovery (%)	Standard Deviation	RSD (%)	LOD (mg/kg)	LOQ (mg/kg)
Trifloxystrobin	Groundnuts	109.2	0.0008	7.2	0.0023	0.008
Trifloxystrobin	Soybeans	115.1	0.0012	10.2	0.0035	0.012
Trifloxystrobin	Beans	72.8	0.0010	13.9	0.0030	0.010
Trifloxystrobin	Chili pepper	73.0	0.0013	18.2	0.0040	0.013
Trifloxystrobin	Egusi seeds	10.5	0.0002	18.5	0.0006	0.002
Trifloxystrobin	Coffee	86.6	0.0016	19.0	0.0049	0.016
Trifloxystrobin	Cocoa	95.0	0.0002	2.5	0.0007	0.002
Trifloxystrobin	Maize					
Trifloxystrobin	White pepper					
**GC-ECD**						
Heptachlor	Groundnuts	99.4	0.0008	8.9	0.0024	0.0080
Heptachlor	Soybeans	126.8	0.0011	6.0	0.0034	0.0114
Heptachlor	Beans	165.9	0.0005	1.9	0.0014	0.0047
Heptachlor	Chili pepper	112.3	0.0060	4.5	0.0181	0.0604
Heptachlor	Egusi seeds	102.2	0.0005	5.4	0.0015	0.0050
Heptachlor	Coffee	121.9	0.0015	8.2	0.0045	0.0151
Heptachlor	Cocoa	184.3	0.0020	7.3	0.0061	0.0202
Heptachlor	Maize	130.3	0.0014	7.1	0.0042	0.0139
Heptachlor	White pepper	104.5	0.0016	13.8	0.0048	0.0159
β-HCH	Groundnuts	129.7	0.0010	5.0	0.0029	0.0097
β-HCH	Soybeans	165.8	0.0015	6.0	0.0044	0.0149
β-HCH	Beans	152.2	0.0003	1.5	0.0010	0.0034
β-HCH	Chili pepper	134.7	0.0095	8.6	0.0284	0.0950
β-HCH	Egusi seeds	276.9	0.0035	8.4	0.0105	0.0350
β-HCH	Coffee	146.1	0.0005	2.4	0.0016	0.0053
β-HCH	Cocoa	160.9	0.0002	0.7	0.0005	0.0016
β-HCH	Maize	114.0	0.0015	8.5	0.0043	0.0145
β-HCH	White pepper	97.3	0.0013	14.7	0.0039	0.0130
Chlorothalonil	Groundnuts	68.2	0.0002	3.1	0.0006	0.0019
Chlorothalonil	Soybeans	104.7	0.0004	2.9	0.0013	0.0045
Chlorothalonil	Beans	58.0	0.0001	2.4	0.0004	0.0012
Chlorothalonil	Chili pepper	126.1	0.0032	4.1	0.0096	0.0320
Chlorothalonil	Egusi seeds	159.8	0.0002	2.3	0.0007	0.0025
Chlorothalonil	Coffee	126.1	0.0031	22.2	0.0092	0.0308
Chlorothalonil	Cocoa	228.8	0.0014	4.1	0.0042	0.0140
Chlorothalonil	Maize	112.2	0.0010	7.9	0.0029	0.0097
Chlorothalonil	White pepper	92.5	0.0004	5.2	0.0013	0.0044
Alachlor	Groundnuts	64.7	0.0058	9.9	0.0174	0.0582
Alachlor	Soybeans	54.0	0.0015	4.2	0.0045	0.0152
Alachlor	Beans	127.8	0.0166	8.7	0.0497	0.1662
Alachlor	Chili pepper	54.8	0.0149	14.9	0.0444	0.1486
Alachlor	Egusi seeds	163.4	0.0117	10.7	0.0349	0.1168

Compound	Food item	Recovery (%)	Standard Deviation	RSD (%)	LOD (mg/kg)	LOQ (mg/kg)
Alachlor	Coffee	104.2	0.0024	2.5	0.0072	0.0242
Alachlor	Cocoa	112.1	0.0046	2.7	0.0137	0.0457
Alachlor	Maize	104.0	0.0060	6.3	0.0179	0.0599
Alachlor	White pepper	70.0	0.0091	14.3	0.0273	0.0912
Aldrin	Groundnuts	53.0	0.0004	7.3	0.0010	0.0035
Aldrin	Soybeans	104.8	0.0006	5.8	0.0017	0.0056
Aldrin	Beans	99.4	0.0003	2.4	0.0008	0.0026
Aldrin	Chili pepper	116.0	0.0009	2.5	0.0026	0.0089
Aldrin	Egusi seeds	52.8	0.0003	14.7	0.0010	0.0033
Aldrin	Coffee	87.8	0.0002	2.1	0.0005	0.0017
Aldrin	Cocoa	186.3	0.0055	6.0	0.0166	0.0555
Aldrin	Maize	84.6	0.0002	2.8	0.0007	0.0022
Aldrin	White pepper	43.7	0.0004	11.1	0.0013	0.0044
Hexachlorobenzene	Groundnuts	107.1	0.0016	13.3	0.0047	0.0157
Hexachlorobenzene	Soybeans	124.9	0.0001	1.2	0.0004	0.0014
Hexachlorobenzene	Beans	190.3	0.0002	0.7	0.0006	0.0021
Hexachlorobenzene	Chili pepper	77.4	0.0012	5.4	0.0037	0.0125
Hexachlorobenzene	Egusi seeds	278.6	0.0030	3.9	0.0091	0.0304
Hexachlorobenzene	Coffee	131.6	0.0005	2.3	0.0014	0.0046
Hexachlorobenzene	Cocoa	141.4	0.0022	10.5	0.0067	0.0224
Hexachlorobenzene	Maize	106.6	0.0012	7.7	0.0037	0.0123
Hexachlorobenzene	White pepper	209.0	0.0042	13.3	0.0125	0.0417
Captan	Groundnuts	135.7	0.0045	3.0	0.0135	0.0450
Captan	Soybeans	181.3	0.0114	5.7	0.0342	0.1145
Captan	Beans	138.8	0.0039	1.9	0.0117	0.0390
Captan	Chili pepper	86.3	0.0017	6.6	0.0051	0.0170
Captan	Egusi seeds	120.5	0.0026	14.3	0.0077	0.0258
Captan	Coffee	134.9	0.0031	15.5	0.0094	0.0314
Captan	Cocoa	198.3	0.0039	13.0	0.0116	0.0387
Captan	Maize	141.2	0.0038	17.9	0.0113	0.0379
Captan	White pepper	201.3	0.0086	10.4	0.0256	0.0858
α-Endosulfan	Groundnuts	114.9	0.0012	6.8	0.0035	0.0117
α-Endosulfan	Soybeans	136.1	0.0007	3.2	0.0020	0.0065
α-Endosulfan	Beans	152.1	0.0006	2.4	0.0017	0.0056
α-Endosulfan	Chili pepper	128.8	0.0007	1.8	0.0021	0.0070
α-Endosulfan	Egusi seeds	111.3	0.0012	9.8	0.0036	0.0120
α-Endosulfan	Coffee	121.5	0.0009	4.8	0.0026	0.0088
α-Endosulfan	Cocoa	127.3	0.0035	25.2	0.0105	0.0353
α-Endosulfan	Maize	161.3	0.0023	3.3	0.0068	0.0227
α-Endosulfan	White pepper	118.5	0.0003	2.2	0.0008	0.0028
p,p'-DDE	Groundnuts	125.2	0.0004	3.8	0.0013	0.0044
p,p'-DDE	Soybeans	132.1	0.0007	3.8	0.0022	0.0075

Compound	Food item	Recovery (%)	Standard Deviation	RSD (%)	LOD (mg/kg)	LOQ (mg/kg)
p,p'-DDE	Beans	160.3	0.0004	1.6	0.0011	0.0037
p,p'-DDE	Chili pepper	176.6	0.0022	4.2	0.0067	0.0225
p,p'-DDE	Egusi seeds	107.9	0.0008	8.0	0.0024	0.0079
p,p'-DDE	Coffee	119.2	0.0006	3.6	0.0019	0.0064
p,p'-DDE	Cocoa	130.6	0.0018	9.3	0.0054	0.0182
p,p'-DDE	Maize	126.4	0.0006	4.0	0.0017	0.0055
p,p'-DDE	White pepper	107.4	0.0008	8.7	0.0025	0.0084
Dieldrin	Groundnuts	88.3	0.0002	2.1	0.0005	0.0016
Dieldrin	Soybeans	116.4	0.0022	17.0	0.0065	0.0217
Dieldrin	Beans	108.0	0.0008	5.2	0.0025	0.0084
Dieldrin	Chili pepper	201.5	0.0006	1.0	0.0018	0.0061
Dieldrin	Egusi seeds	64.4	0.0003	6.0	0.0008	0.0026
Dieldrin	Coffee	180.9	0.0010	3.7	0.0030	0.0101
Dieldrin	Cocoa	116.2	0.0025	14.6	0.0076	0.0255
Dieldrin	Maize	94.0	0.0005	5.8	0.0015	0.0050
Dieldrin	White pepper	220.2	0.0055	16.6	0.0164	0.0548
Endrin	Groundnuts	103.1	0.0003	2.1	0.0010	0.0032
Endrin	Soybeans	150.9	0.0014	6.3	0.0043	0.0142
Endrin	Beans	174.9	0.0008	3.1	0.0024	0.0081
Endrin	Chili pepper	229.8	0.0012	1.7	0.0035	0.0117
Endrin	Egusi seeds	111.6	0.0017	13.6	0.0050	0.0167
Endrin	Coffee	129.6	0.0013	6.5	0.0038	0.0127
Endrin	Cocoa	155.2	0.0015	6.3	0.0044	0.0146
Endrin	Maize	113.7	0.0014	8.2	0.0042	0.0140
Endrin	White pepper	147.6	0.0034	15.2	0.0101	0.0337
p,p'-DDD/β-Endosulfan	Groundnuts	131.6	0.0003	1.9	0.0008	0.0028
p,p'-DDD/β-Endosulfan	Soybeans	132.0	0.0008	4.0	0.0024	0.0080
p,p'-DDD/β-Endosulfan	Beans	140.6	0.0007	3.4	0.0021	0.0071
p,p'-DDD/β-Endosulfan	Chili pepper	58.4	0.0009	4.9	0.0026	0.0085
p,p'-DDD/β-Endosulfan	Egusi seeds	119.5	0.0008	9.7	0.0023	0.0077
p,p'-DDD/β-Endosulfan	Coffee	121.2	0.0007	4.1	0.0022	0.0075
p,p'-DDD/β-Endosulfan	Cocoa	137.9	0.0020	9.7	0.0060	0.0201
p,p'-DDD/β-Endosulfan	Maize	132.7	0.0002	1.6	0.0007	0.0023
p,p'-DDD/β-Endosulfan	White pepper	100.3	0.0024	26.1	0.0071	0.0238
o,p'-DDT	Groundnuts	134.2	0.0011	5.4	0.0032	0.0108
o,p'-DDT	Soybeans	182.6	0.0011	4.1	0.0034	0.0113
o,p'-DDT	Beans	205.4	0.0005	1.6	0.0015	0.0049

Compound	Food item	Recovery (%)	Standard Deviation	RSD (%)	LOD (mg/kg)	LOQ (mg/kg)
o,p'-DDT	Chili pepper	140.9	0.0008	2.0	0.0025	0.0084
o,p'-DDT	Egusi seeds	170.8	0.0008	3.2	0.0025	0.0083
o,p'-DDT	Coffee	195.1	0.0016	5.3	0.0047	0.0156
o,p'-DDT	Cocoa	169.3	0.0006	2.4	0.0018	0.0060
o,p'-DDT	Maize	149.5	0.0012	5.3	0.0036	0.0119
o,p'-DDT	White pepper	213.0	0.0019	5.8	0.0056	0.0186
p,p'-DDT	Groundnuts	171.4	0.0022	4.4	0.0067	0.0224
p,p'-DDT	Soybeans	170.1	0.0042	8.2	0.0125	0.0416
p,p'-DDT	Beans	163.5	0.0008	1.5	0.0023	0.0075
p,p'-DDT	Chili pepper	88.2	0.0005	1.0	0.0015	0.0050
p,p'-DDT	Egusi seeds	280.1	0.0018	1.1	0.0055	0.0184
p,p'-DDT	Coffee	264.8	0.0060	7.5	0.0178	0.0596
p,p'-DDT	Cocoa	125.1	0.0014	5.2	0.0043	0.0143
p,p'-DDT	Maize	137.0	0.0020	4.8	0.0059	0.0197
p,p'-DDT	White pepper	107.8	0.0007	2.8	0.0020	0.0067
Bifenthrin	Groundnuts	135.6	0.0007	4.8	0.0021	0.0071
Bifenthrin	Soybeans	146.1	0.0009	4.2	0.0028	0.0093
Bifenthrin	Beans	160.6	0.0007	2.7	0.0020	0.0065
Bifenthrin	Chili pepper	130.2	0.0076	7.7	0.0228	0.0764
Bifenthrin	Egusi seeds	128.5	0.0010	5.3	0.0030	0.0101
Bifenthrin	Coffee	122.9	0.0015	8.4	0.0046	0.0154
Bifenthrin	Cocoa	131.6	0.0022	11.4	0.0067	0.0224
Bifenthrin	Maize	198.2	0.0062	20.4	0.0186	0.0622
Bifenthrin	White pepper	208.9	0.0033	10.6	0.0100	0.0333
Methoxychlor	Groundnuts	127.1	0.0033	8.7	0.0099	0.0331
Methoxychlor	Soybeans	85.6	0.0036	14.0	0.0107	0.0358
Methoxychlor	Beans	186.9	0.0062	11.1	0.0186	0.0623
Methoxychlor	Chili pepper	153.8	0.0218	9.5	0.0652	0.2180
Methoxychlor	Egusi seeds	134.1	0.0040	13.7	0.0120	0.0403
Methoxychlor	Coffee	127.4	0.0026	9.3	0.0078	0.0262
Methoxychlor	Cocoa	195.2	0.0103	9.2	0.0307	0.1028
Methoxychlor	Maize	198.9	0.0070	11.7	0.0209	0.0700
Methoxychlor	White pepper	212.1	0.0044	3.5	0.0130	0.0436
Cypermethrin	Groundnuts	179.3	0.0015	3.4	0.0046	0.0154
Cypermethrin	Soybeans	138.7	0.0018	8.7	0.0054	0.0180
Cypermethrin	Beans	162.0	0.0011	4.6	0.0033	0.0111
Cypermethrin	Chili pepper	159.0	0.0012	2.4	0.0035	0.0117
Cypermethrin	Egusi seeds	278.3	0.0014	1.3	0.0043	0.0144
Cypermethrin	Coffee	268.6	0.0152	4.6	0.0454	0.1519
Cypermethrin	Cocoa	203.2	0.0037	12.2	0.0111	0.0370
Cypermethrin	Maize	159.2	0.0023	9.7	0.0070	0.0232
Cypermethrin	White pepper	122.4	0.0012	8.9	0.0036	0.0120

Table S2. Analysis of 99 pesticide residues in 72 samples of 12 agricultural products from western highlands of Cameroon by LC-MS/MS and GC-ECD

Pesticide	Location	Food item	Mean (mg/kg)	EU MRL (mg/kg)	Mean > MRL?
Acetamiprid	Dschang	Chili pepper	0.0400	0.05	No
Acetamiprid	Bafoussam	Cowpea	0.0054	0.15	No
Acetamiprid	Bangangté	Cowpea	0.0033	0.15	No
Acetamiprid	Bafang	Cowpea	0.0004	0.15	No
Acetamiprid	Mbouda	Kidney beans	0.0017	0.15	No
Acetamiprid	Mbouda	Soybeans	0.0007	0.01	No
Alachlor	Bafoussam	Bambara nuts	0.2383	NA	No
Alachlor	Foumban	Bambara nuts	0.0264	NA	No
Alachlor	Bafang	Bambara nuts	0.0121	NA	No
Alachlor	Mbouda	Black beans	0.0425	0.01	Yes
Alachlor	Bangangté	Black beans	0.0330	0.01	Yes
Alachlor	Bafoussam	Black beans	0.0243	0.01	Yes
Alachlor	Bafang	Black beans	0.0238	0.01	Yes
Alachlor	Dschang	Chili pepper	0.0971	0.05	Yes
Alachlor	Foumban	Chili pepper	0.0560	0.05	Yes
Alachlor	Bafang	Chili pepper	0.0435	0.05	No
Alachlor	Bangangté	Chili pepper	0.0367	0.05	No
Alachlor	Bafoussam	Chili pepper	0.0272	0.05	No
Alachlor	Bangangté	Cocoa	0.0120	0.05	No
Alachlor	Foumban	Coffee	0.0448	0.05	No
Alachlor	Bafang	Coffee	0.0172	0.05	No
Alachlor	Bangangté	Coffee	0.0156	0.05	No
Alachlor	Foumban	Coffee	0.0123	0.05	No
Alachlor	Foumban	Cowpea	0.0356	0.01	Yes
Alachlor	Mbouda	Cowpea	0.0201	0.01	Yes
Alachlor	Bangangté	Cowpea	0.0142	0.01	Yes
Alachlor	Bafang	Cowpea	0.0132	0.01	Yes
Alachlor	Dschang	Cowpea	0.0099	0.01	No
Alachlor	Bafoussam	Cowpea	0.0092	0.01	No
Alachlor	Bafoussam	Egusi seeds	0.0226	NA	No
Alachlor	Mbouda	Egusi seeds	0.0045	NA	No
Alachlor	Bafoussam	Groundnuts	0.0123	0.02	No
Alachlor	Foumban	Groundnuts	0.0088	0.02	No
Alachlor	Bafoussam	Groundnuts	0.0060	0.02	No
Alachlor	Bafoussam	Kidney beans	0.2490	0.01	Yes
Alachlor	Bafang	Kidney beans	0.1930	0.01	Yes
Alachlor	Mbouda	Kidney beans	0.0271	0.01	Yes
Alachlor	Bangangté	Kidney beans	0.0132	0.01	Yes

Pesticide	Location	Food item	Mean (mg/kg)	EU MRL (mg/kg)	Mean > MRL?
Alachlor	Dschang	Kidney beans	0.0128	0.01	Yes
Alachlor	Foumban	Kidney beans	0.0121	0.01	Yes
Alachlor	Dschang	Kidney beans	0.0082	0.01	No
Alachlor	Dschang	Kidney beans	0.0078	0.01	No
Alachlor	Bangangté	Kidney beans	0.0073	0.01	No
Alachlor	Dschang	Maize	0.0090	0.01	No
Alachlor	Dschang	Maize	0.0062	0.01	No
Alachlor	Bafang	Maize	0.0060	0.01	No
Alachlor	Bangangté	Maize	0.0026	0.01	No
Alachlor	Bafoussam	Soybeans	0.3214	0.02	Yes
Alachlor	Mbouda	Soybeans	0.1057	0.02	Yes
Alachlor	Bafang	Soybeans	0.0348	0.02	Yes
Alachlor	Foumban	Soybeans	0.0307	0.02	Yes
Alachlor	Dschang	Soybeans	0.0157	0.02	No
Alachlor	Bafoussam	White pepper	0.6275	0.05	Yes
Alachlor	Foumban	White pepper	0.3209	0.05	Yes
Aldrin	Foumban	Bambara nuts	0.1295	NA	No
Aldrin	Bafang	Bambara nuts	0.0082	NA	No
Aldrin	Bafang	Black beans	0.3039	0.01	Yes
Aldrin	Mbouda	Black beans	0.2848	0.01	Yes
Aldrin	Bafoussam	Black beans	0.1831	0.01	Yes
Aldrin	Bangangté	Black beans	0.0937	0.01	Yes
Aldrin	Bafoussam	Chili pepper	0.0883	0.1	No
Aldrin	Dschang	Chili pepper	0.0331	0.1	No
Aldrin	Bafang	Chili pepper	0.0267	0.1	No
Aldrin	Foumban	Chili pepper	0.0215	0.1	No
Aldrin	Bafoussam	Chili pepper	0.0126	0.1	No
Aldrin	Bangangté	Chili pepper	0.0022	0.1	No
Aldrin	Bafang	Cocoa	0.0584	0.05	Yes
Aldrin	Bangangté	Cocoa	0.0330	0.05	No
Aldrin	Foumban	Coffee	0.0096	0.02	No
Aldrin	Bangangté	Cowpea	0.0760	0.01	Yes
Aldrin	Bafoussam	Cowpea	0.0430	0.01	Yes
Aldrin	Bafoussam	Cowpea	0.0191	0.01	Yes
Aldrin	Mbouda	Cowpea	0.0117	0.01	Yes
Aldrin	Bafang	Cowpea	0.0077	0.01	No
Aldrin	Mbouda	Egusi seeds	0.2158	NA	No
Aldrin	Bafoussam	Egusi seeds	0.0913	NA	No
Aldrin	Mbouda	Egusi seeds	0.0729	NA	No
Aldrin	Bafoussam	Egusi seeds	0.0520	NA	No
Aldrin	Bafang	Egusi seeds	0.0175	NA	No

Pesticide	Location	Food item	Mean (mg/kg)	EU MRL (mg/kg)	Mean > MRL?
Aldrin	Foumban	Egusi seeds	0.0091	NA	No
Aldrin	Mbouda	Groundnuts	0.0485	0.02	Yes
Aldrin	Bafoussam	Groundnuts	0.0181	0.02	No
Aldrin	Bafang	Groundnuts	0.0164	0.02	No
Aldrin	Bangangté	Groundnuts	0.0103	0.02	No
Aldrin	Bafoussam	Groundnuts	0.0101	0.02	No
Aldrin	Foumban	Groundnuts	0.0054	0.02	No
Aldrin	Mbouda	Groundnuts	0.0042	0.02	No
Aldrin	Bafoussam	Kidney beans	0.4342	0.01	Yes
Aldrin	Mbouda	Kidney beans	0.2241	0.01	Yes
Aldrin	Bangangté	Kidney beans	0.1731	0.01	Yes
Aldrin	Foumban	Kidney beans	0.1145	0.01	Yes
Aldrin	Bafang	Kidney beans	0.1142	0.01	Yes
Aldrin	Dschang	Kidney beans	0.0428	0.01	Yes
Aldrin	Mbouda	Kidney beans	0.0292	0.01	Yes
Aldrin	Bafang	Kidney beans	0.0286	0.01	Yes
Aldrin	Bangangté	Kidney beans	0.0227	0.01	Yes
Aldrin	Mbouda	Kidney beans	0.0116	0.01	Yes
Aldrin	Dschang	Kidney beans	0.0071	0.01	No
Aldrin	Dschang	Kidney beans	0.0012	0.01	No
Aldrin	Foumban	Maize	0.4646	0.01	Yes
Aldrin	Bangangté	Maize	0.1273	0.01	Yes
Aldrin	Mbouda	Maize	0.0812	0.01	Yes
Aldrin	Mbouda	Maize	0.0141	0.01	Yes
Aldrin	Bafang	Maize	0.0128	0.01	Yes
Aldrin	Bafoussam	Maize	0.0119	0.01	Yes
Aldrin	Bafoussam	Maize	0.0106	0.01	Yes
Aldrin	Bangangté	Maize	0.0076	0.01	No
Aldrin	Bafang	Maize	0.0047	0.01	No
Aldrin	Dschang	Maize	0.0026	0.01	No
Aldrin	Dschang	Maize	0.0019	0.01	No
Aldrin	Bafoussam	Soybeans	0.0321	0.02	Yes
Aldrin	Dschang	Soybeans	0.0220	0.02	Yes
Aldrin	Mbouda	Soybeans	0.0189	0.02	No
Aldrin	Bafoussam	Soybeans	0.0155	0.02	No
Aldrin	Bafang	Soybeans	0.0088	0.02	No
Aldrin	Foumban	Soybeans	0.0043	0.02	No
Aldrin	Bafoussam	White pepper	0.1050	0.1	Yes
Aldrin	Foumban	White pepper	0.0737	0.1	No
Atrazine	Bafang	Bambara nuts	0.0008	NA	No
Atrazine	Bafoussam	Bambara nuts	0.0006	NA	No
Atrazine	Bafang	Chili pepper	0.0032	0.1	No

Pesticide	Location	Food item	Mean (mg/kg)	EU MRL (mg/kg)	Mean > MRL?
Atrazine	Dschang	Chili pepper	0.0031	0.1	No
Atrazine	Mbouda	Chili pepper	0.0028	0.1	No
Atrazine	Bafoussam	Chili pepper	0.0021	0.1	No
Atrazine	Foumban	Chili pepper	0.0015	0.1	No
Atrazine	Bafoussam	Chili pepper	0.0010	0.1	No
Atrazine	Bafang	Cocoa	0.0011	0.1	No
Atrazine	Bafang	Coffee	0.0028	0.1	No
Atrazine	Bafoussam	Cowpea	0.0032	0.05	No
Atrazine	Bafang	Cowpea	0.0028	0.05	No
Atrazine	Bafoussam	Cowpea	0.0010	0.05	No
Atrazine	Bangangté	Cowpea	0.0010	0.05	No
Atrazine	Dschang	Cowpea	0.0009	0.05	No
Atrazine	Mbouda	Egusi seeds	0.0014	NA	No
Atrazine	Bafang	Egusi seeds	0.0012	NA	No
Atrazine	Foumban	Egusi seeds	0.0009	NA	No
Atrazine	Mbouda	Egusi seeds	0.0009	NA	No
Atrazine	Bafoussam	Egusi seeds	0.0007	NA	No
Atrazine	Bangangté	Groundnuts	0.0020	0.05	No
Atrazine	Bafoussam	Kidney beans	0.0017	0.05	No
Atrazine	Mbouda	Kidney beans	0.0013	0.05	No
Atrazine	Foumban	Kidney beans	0.0010	0.05	No
Atrazine	Bafoussam	Maize	0.0040	0.05	No
Atrazine	Mbouda	Soybeans	0.0030	0.05	No
Atrazine	Bangangté	Soybeans	0.0012	0.05	No
Atrazine	Bafoussam	White pepper	0.0028	0.1	No
Atrazine	Foumban	White pepper	0.0012	0.1	No
Azoxystrobin	Mbouda	Chili pepper	0.0011	0.3	No
Benalaxyl	Bafoussam	White pepper	0.0222	0.1	No
Benalaxyl	Foumban	White pepper	0.0046	0.1	No
Bentazon	Bafoussam	Bambara nuts	0.0128	NA	No
Bentazon	Bafoussam	Chili pepper	0.0020	0.1	No
Bentazon	Bafoussam	Egusi seeds	0.0068	NA	No
Bentazon	Bafoussam	Egusi seeds	0.0062	NA	No
Bentazon	Mbouda	Egusi seeds	0.0052	NA	No
Bifenthrin	Mbouda	Kidney beans	0.0086	0.3	No
Bifenthrin	Bafoussam	Maize	0.0310	0.05	No
Bifenthrin	Bafoussam	Maize	0.0106	0.05	No
Bifenthrin	Bafoussam	Soybeans	0.0056	0.3	No
Bitertanol	Foumban	Bambara nuts	0.0093	NA	No
Bitertanol	Bafang	Maize	0.0018	0.01	No
Bitertanol	Bafoussam	Soybeans	0.0015	0.02	No
Bitertanol	Mbouda	Soybeans	0.0008	0.02	No

Pesticide	Location	Food item	Mean (mg/kg)	EU MRL (mg/kg)	Mean > MRL?
Cadusafos	Mbouda	Chili pepper	0.6285	0.01	Yes
Cadusafos	Foumban	Chili pepper	0.0235	0.01	Yes
Cadusafos	Bafang	Chili pepper	0.0128	0.01	Yes
Cadusafos	Bafoussam	Chili pepper	0.0089	0.01	No
Captan	Foumban	Bambara nuts	0.0577	NA	No
Captan	Bafoussam	Bambara nuts	0.0204	NA	No
Captan	Bafoussam	Black beans	0.0653	0.07	No
Captan	Bangangté	Black beans	0.0275	0.07	No
Captan	Bafoussam	Chili pepper	0.8557	0.1	Yes
Captan	Bafang	Chili pepper	0.3795	0.1	Yes
Captan	Bangangté	Chili pepper	0.3658	0.1	Yes
Captan	Bafoussam	Chili pepper	0.0902	0.1	No
Captan	Dschang	Chili pepper	0.0313	0.1	No
Captan	Foumban	Chili pepper	0.0280	0.1	No
Captan	Bafang	Cocoa	0.0143	0.1	No
Captan	Bangangté	Cocoa	0.0097	0.1	No
Captan	Foumban	Coffee	0.4898	0.1	Yes
Captan	Bangangté	Coffee	0.2740	0.1	Yes
Captan	Foumban	Coffee	0.0697	0.1	No
Captan	Bafang	Coffee	0.0227	0.1	No
Captan	Bafoussam	Cowpea	0.0346	0.07	No
Captan	Foumban	Cowpea	0.0187	0.07	No
Captan	Bafang	Cowpea	0.0173	0.07	No
Captan	Bafoussam	Cowpea	0.0161	0.07	No
Captan	Bangangté	Cowpea	0.0138	0.07	No
Captan	Bafoussam	Egusi seeds	0.1363	NA	No
Captan	Mbouda	Egusi seeds	0.1299	NA	No
Captan	Bangangté	Egusi seeds	0.0676	NA	No
Captan	Bafoussam	Egusi seeds	0.0414	NA	No
Captan	Bafang	Groundnuts	0.8065	0.07	Yes
Captan	Dschang	Groundnuts	0.7616	0.07	Yes
Captan	Mbouda	Groundnuts	0.7069	0.07	Yes
Captan	Mbouda	Groundnuts	0.5996	0.07	Yes
Captan	Bangangté	Groundnuts	0.2506	0.07	Yes
Captan	Foumban	Groundnuts	0.1637	0.07	Yes
Captan	Bafoussam	Groundnuts	0.0183	0.07	No
Captan	Mbouda	Kidney beans	0.8366	0.07	Yes
Captan	Dschang	Kidney beans	0.2149	0.07	Yes
Captan	Bafoussam	Kidney beans	0.1634	0.07	Yes
Captan	Mbouda	Kidney beans	0.0815	0.07	Yes
Captan	Foumban	Kidney beans	0.0375	0.07	No
Captan	Bangangté	Kidney beans	0.0263	0.07	No

Pesticide	Location	Food item	Mean (mg/kg)	EU MRL (mg/kg)	Mean > MRL?
Captan	Bafang	Kidney beans	0.0256	0.07	No
Captan	Dschang	Kidney beans	0.0232	0.07	No
Captan	Bafang	Kidney beans	0.0178	0.07	No
Captan	Foumban	Maize	0.1163	0.07	Yes
Captan	Bangangté	Maize	0.0862	0.07	Yes
Captan	Bafoussam	Maize	0.0715	0.07	Yes
Captan	Mbouda	Maize	0.0335	0.07	No
Captan	Bafang	Maize	0.0209	0.07	No
Captan	Bangangté	Maize	0.0190	0.07	No
Captan	Bafoussam	Maize	0.0167	0.07	No
Captan	Dschang	Maize	0.0149	0.07	No
Captan	Mbouda	Maize	0.0142	0.07	No
Captan	Bafang	Maize	0.0139	0.07	No
Captan	Dschang	Maize	0.0134	0.07	No
Captan	Dschang	Soybeans	0.0524	0.07	No
Captan	Bafoussam	Soybeans	0.0406	0.07	No
Captan	Bafoussam	Soybeans	0.0327	0.07	No
Captan	Mbouda	Soybeans	0.0160	0.07	No
Captan	Foumban	Soybeans	0.0140	0.07	No
Captan	Bafang	Soybeans	0.0113	0.07	No
Captan	Foumban	White pepper	0.1550	0.1	Yes
Captan	Bafoussam	White pepper	0.0474	0.1	No
Carbaryl	Mbouda	Kidney beans	0.0297	0.05	No
Carbaryl	Bafoussam	White pepper	0.0758	0.8	No
Carbendazim	Mbouda	Kidney beans	0.0014	0.1	No
Carbofuran	Dschang	Chili pepper	0.0027	0.05	No
Carbofuran	Foumban	Chili pepper	0.0011	0.05	No
Carbofuran	Bangangté	Cowpea	0.0006	0.005	No
Chlorothalonil	Bafoussam	Bambara nuts	0.0150	NA	No
Chlorothalonil	Foumban	Bambara nuts	0.0112	NA	No
Chlorothalonil	Bafang	Bambara nuts	0.0093	NA	No
Chlorothalonil	Bangangté	Black beans	0.0108	3	No
Chlorothalonil	Bafoussam	Black beans	0.0090	3	No
Chlorothalonil	Bafang	Chili pepper	0.0683	0.05	Yes
Chlorothalonil	Foumban	Chili pepper	0.0681	0.05	Yes
Chlorothalonil	Dschang	Chili pepper	0.0128	0.05	No
Chlorothalonil	Bafoussam	Chili pepper	0.0060	0.05	No
Chlorothalonil	Bangangté	Chili pepper	0.0045	0.05	No
Chlorothalonil	Bafoussam	Chili pepper	0.0042	0.05	No
Chlorothalonil	Bafang	Cocoa	0.0081	0.05	No
Chlorothalonil	Bangangté	Cocoa	0.0069	0.05	No
Chlorothalonil	Foumban	Coffee	0.0130	0.05	No

Pesticide	Location	Food item	Mean (mg/kg)	EU MRL (mg/kg)	Mean > MRL?
Chlorothalonil	Foumban	Coffee	0.0119	0.05	No
Chlorothalonil	Bangangté	Coffee	0.0112	0.05	No
Chlorothalonil	Bafang	Coffee	0.0105	0.05	No
Chlorothalonil	Bafoussam	Cowpea	0.0172	3	No
Chlorothalonil	Bafang	Cowpea	0.0097	3	No
Chlorothalonil	Bafoussam	Cowpea	0.0095	3	No
Chlorothalonil	Foumban	Egusi seeds	0.0129	NA	No
Chlorothalonil	Bangangté	Egusi seeds	0.0052	NA	No
Chlorothalonil	Bafoussam	Egusi seeds	0.0048	NA	No
Chlorothalonil	Mbouda	Egusi seeds	0.0039	NA	No
Chlorothalonil	Bafoussam	Groundnuts	0.0092	0.1	No
Chlorothalonil	Bafoussam	Groundnuts	0.0090	0.1	No
Chlorothalonil	Foumban	Groundnuts	0.0077	0.1	No
Chlorothalonil	Bangangté	Groundnuts	0.0074	0.1	No
Chlorothalonil	Bafoussam	Kidney beans	0.0371	3	No
Chlorothalonil	Bafang	Kidney beans	0.0145	3	No
Chlorothalonil	Dschang	Kidney beans	0.0115	3	No
Chlorothalonil	Bangangté	Kidney beans	0.0108	3	No
Chlorothalonil	Dschang	Kidney beans	0.0100	3	No
Chlorothalonil	Bangangté	Kidney beans	0.0099	3	No
Chlorothalonil	Dschang	Kidney beans	0.0089	3	No
Chlorothalonil	Mbouda	Kidney beans	0.0056	3	No
Chlorothalonil	Mbouda	Maize	0.0074	0.01	No
Chlorothalonil	Bafoussam	Maize	0.0048	0.01	No
Chlorothalonil	Bangangté	Maize	0.0047	0.01	No
Chlorothalonil	Dschang	Maize	0.0045	0.01	No
Chlorothalonil	Bafang	Maize	0.0045	0.01	No
Chlorothalonil	Bafoussam	Soybeans	0.0091	0.01	No
Chlorothalonil	Mbouda	Soybeans	0.0091	0.01	No
Chlorothalonil	Bafang	Soybeans	0.0073	0.01	No
Chlorothalonil	Dschang	Soybeans	0.0049	0.01	No
Chlorothalonil	Foumban	Soybeans	0.0049	0.01	No
Chlorothalonil	Foumban	White pepper	0.0301	0.05	No
Chlorothalonil	Bafoussam	White pepper	0.0158	0.05	No
Chlorpyrifos	Foumban	Bambara nuts	0.0532	NA	No
Chlorpyrifos	Dschang	Chili pepper	0.0071	1	No
Chlorpyrifos	Bangangté	Cowpea	0.0907	0.05	Yes
Chlorpyrifos	Foumban	Kidney beans	0.0460	0.05	No
Chlorpyrifos	Foumban	Maize	0.3667	0.05	Yes
Chlorpyrifos	Bangangté	Maize	0.1292	0.05	Yes
Chlorotoluron	Bafang	Chili pepper	0.2318	0.05	Yes
Chlorotoluron	Mbouda	Chili pepper	0.1083	0.05	Yes

Pesticide	Location	Food item	Mean (mg/kg)	EU MRL (mg/kg)	Mean > MRL?
Chlorotoluron	Foumban	Chili pepper	0.0759	0.05	Yes
Chlorotoluron	Bafang	Coffee	0.1756	0.05	Yes
Chlorotoluron	Bafoussam	Cowpea	0.2752	0.01	Yes
Chlorotoluron	Bangangté	Egusi seeds	0.1466	NA	No
Chlorotoluron	Bafang	Maize	0.0846	0.01	Yes
Chlorotoluron	Dschang	Maize	0.0715	0.01	Yes
Chlorotoluron	Foumban	Maize	0.0709	0.01	Yes
Chlorotoluron	Bafoussam	Soybeans	1.5508	0.02	Yes
Chlorotoluron	Bafoussam	Soybeans	0.0788	0.02	Yes
Cypermethrin	Foumban	Bambara nuts	0.0103	NA	No
Cypermethrin	Bafoussam	Bambara nuts	0.0071	NA	No
Cypermethrin	Bafang	Black beans	0.0527	0.05	Yes
Cypermethrin	Bangangté	Black beans	0.0133	0.05	No
Cypermethrin	Bafoussam	Chili pepper	0.5502	0.1	Yes
Cypermethrin	Bafoussam	Chili pepper	0.2758	0.1	Yes
Cypermethrin	Bafang	Chili pepper	0.1143	0.1	Yes
Cypermethrin	Foumban	Chili pepper	0.0541	0.1	No
Cypermethrin	Dschang	Chili pepper	0.0280	0.1	No
Cypermethrin	Bangangté	Chili pepper	0.0142	0.1	No
Cypermethrin	Bangangté	Cocoa	0.0231	0.1	No
Cypermethrin	Bafang	Cocoa	0.0036	0.1	No
Cypermethrin	Foumban	Coffee	0.1286	0.1	Yes
Cypermethrin	Foumban	Coffee	0.0655	0.1	No
Cypermethrin	Bangangté	Coffee	0.0188	0.1	No
Cypermethrin	Bafang	Coffee	0.0082	0.1	No
Cypermethrin	Foumban	Cowpea	0.0039	0.05	No
Cypermethrin	Bangangté	Cowpea	0.0027	0.05	No
Cypermethrin	Bangangté	Egusi seeds	0.0315	NA	No
Cypermethrin	Foumban	Egusi seeds	0.0176	NA	No
Cypermethrin	Bafoussam	Egusi seeds	0.0057	NA	No
Cypermethrin	Mbouda	Groundnuts	0.1370	0.1	Yes
Cypermethrin	Dschang	Groundnuts	0.1099	0.1	Yes
Cypermethrin	Bangangté	Groundnuts	0.0635	0.1	No
Cypermethrin	Mbouda	Groundnuts	0.0586	0.1	No
Cypermethrin	Bafang	Groundnuts	0.0445	0.1	No
Cypermethrin	Foumban	Groundnuts	0.0388	0.1	No
Cypermethrin	Bafoussam	Groundnuts	0.0346	0.1	No
Cypermethrin	Bafang	Kidney beans	0.9449	0.05	Yes
Cypermethrin	Bafoussam	Kidney beans	0.1562	0.05	Yes
Cypermethrin	Mbouda	Kidney beans	0.1055	0.05	Yes
Cypermethrin	Bangangté	Kidney beans	0.0547	0.05	Yes
Cypermethrin	Bangangté	Kidney beans	0.0310	0.05	No

Pesticide	Location	Food item	Mean (mg/kg)	EU MRL (mg/kg)	Mean > MRL?
Cypermethrin	Dschang	Kidney beans	0.0196	0.05	No
Cypermethrin	Bafang	Kidney beans	0.0109	0.05	No
Cypermethrin	Foumban	Kidney beans	0.0094	0.05	No
Cypermethrin	Mbouda	Kidney beans	0.0069	0.05	No
Cypermethrin	Dschang	Kidney beans	0.0029	0.05	No
Cypermethrin	Dschang	Kidney beans	0.0025	0.05	No
Cypermethrin	Foumban	Maize	0.1541	0.3	No
Cypermethrin	Mbouda	Maize	0.0332	0.3	No
Cypermethrin	Bangangté	Maize	0.0100	0.3	No
Cypermethrin	Bafang	Maize	0.0083	0.3	No
Cypermethrin	Bafoussam	Maize	0.0069	0.3	No
Cypermethrin	Dschang	Maize	0.0057	0.3	No
Cypermethrin	Bafang	Maize	0.0046	0.3	No
Cypermethrin	Bafoussam	Maize	0.0014	0.3	No
Cypermethrin	Bafoussam	Soybeans	0.0241	0.05	No
Cypermethrin	Dschang	Soybeans	0.0180	0.05	No
Cypermethrin	Foumban	Soybeans	0.0134	0.05	No
Cypermethrin	Bafoussam	White pepper	0.0573	0.1	No
Cypermethrin	Foumban	White pepper	0.0094	0.1	No
Diazinon	Bafoussam	White pepper	0.0020	0.1	No
Dieldrin	Bafoussam	Bambara nuts	0.0095	NA	No
Dieldrin	Foumban	Bambara nuts	0.0033	NA	No
Dieldrin	Bafang	Bambara nuts	0.0017	NA	No
Dieldrin	Bafang	Black beans	0.0049	0.01	No
Dieldrin	Bangangté	Black beans	0.0019	0.01	No
Dieldrin	Mbouda	Black beans	0.0012	0.01	No
Dieldrin	Bafoussam	Chili pepper	0.0604	0.1	No
Dieldrin	Bafang	Chili pepper	0.0182	0.1	No
Dieldrin	Foumban	Chili pepper	0.0021	0.1	No
Dieldrin	Bafoussam	Cowpea	0.0118	0.01	Yes
Dieldrin	Bafoussam	Cowpea	0.0044	0.01	No
Dieldrin	Bangangté	Cowpea	0.0025	0.01	No
Dieldrin	Dschang	Cowpea	0.0017	0.01	No
Dieldrin	Bafoussam	Egusi seeds	0.0052	NA	No
Dieldrin	Bafoussam	Egusi seeds	0.0045	NA	No
Dieldrin	Mbouda	Egusi seeds	0.0027	NA	No
Dieldrin	Mbouda	Egusi seeds	0.0021	NA	No
Dieldrin	Bangangté	Egusi seeds	0.0020	NA	No
Dieldrin	Bafang	Egusi seeds	0.0016	NA	No
Dieldrin	Mbouda	Groundnuts	0.0021	0.02	No
Dieldrin	Bafoussam	Groundnuts	0.0019	0.02	No
Dieldrin	Foumban	Groundnuts	0.0013	0.02	No

Pesticide	Location	Food item	Mean (mg/kg)	EU MRL (mg/kg)	Mean > MRL?
Dieldrin	Bangangté	Kidney beans	0.0061	0.01	No
Dieldrin	Bangangté	Kidney beans	0.0037	0.01	No
Dieldrin	Foumban	Kidney beans	0.0037	0.01	No
Dieldrin	Dschang	Kidney beans	0.0022	0.01	No
Dieldrin	Mbouda	Kidney beans	0.0021	0.01	No
Dieldrin	Bafang	Kidney beans	0.0019	0.01	No
Dieldrin	Bafang	Kidney beans	0.0019	0.01	No
Dieldrin	Foumban	Maize	0.0399	0.01	Yes
Dieldrin	Mbouda	Maize	0.0190	0.01	Yes
Dieldrin	Dschang	Maize	0.0146	0.01	Yes
Dieldrin	Bafoussam	Maize	0.0127	0.01	Yes
Dieldrin	Bangangté	Maize	0.0091	0.01	No
Dieldrin	Bafang	Maize	0.0048	0.01	No
Dieldrin	Bafoussam	Maize	0.0045	0.01	No
Dieldrin	Dschang	Maize	0.0039	0.01	No
Dieldrin	Bangangté	Maize	0.0031	0.01	No
Dieldrin	Mbouda	Maize	0.0018	0.01	No
Dieldrin	Bafang	Maize	0.0018	0.01	No
Dieldrin	Mbouda	Soybeans	0.0162	0.02	No
Dieldrin	Bafoussam	Soybeans	0.0018	0.02	No
Dieldrin	Bafang	Soybeans	0.0016	0.02	No
Dieldrin	Bafoussam	White pepper	0.0019	0.1	No
Difenconazole	Bafoussam	Chili pepper	0.0021	0.3	No
Difenconazole	Mbouda	Chili pepper	0.0021	0.3	No
Difenconazole	Bafang	Chili pepper	0.0017	0.3	No
Difenconazole	Bangangté	Chili pepper	0.0017	0.3	No
Difenconazole	Dschang	Chili pepper	0.0017	0.3	No
Difenconazole	Bafoussam	Chili pepper	0.0013	0.3	No
Difenconazole	Foumban	Chili pepper	0.0013	0.3	No
Difenconazole	Bafoussam	Cowpea	0.0009	0.06	No
Difenconazole	Bafoussam	Cowpea	0.0009	0.06	No
Difenconazole	Bangangté	Cowpea	0.0009	0.06	No
Difenconazole	Bangangté	Kidney beans	0.0009	0.06	No
Dimethomorph	Foumban	Chili pepper	0.0007	0.05	No
Endrin	Bangangté	Black beans	0.0069	0.01	No
Endrin	Bafang	Black beans	0.0039	0.01	No
Endrin	Mbouda	Black beans	0.0020	0.01	No
Endrin	Bafoussam	Black beans	0.0012	0.01	No
Endrin	Bafoussam	Chili pepper	0.0337	0.1	No
Endrin	Dschang	Chili pepper	0.0137	0.1	No
Endrin	Bangangté	Chili pepper	0.0054	0.1	No
Endrin	Bafang	Chili pepper	0.0020	0.1	No

Pesticide	Location	Food item	Mean (mg/kg)	EU MRL (mg/kg)	Mean > MRL?
Endrin	Bangangté	Coffee	0.0014	0.01	No
Endrin	Foumban	Cowpea	0.0105	0.01	Yes
Endrin	Dschang	Cowpea	0.0070	0.01	No
Endrin	Mbouda	Cowpea	0.0038	0.01	No
Endrin	Bafoussam	Cowpea	0.0028	0.01	No
Endrin	Bafoussam	Cowpea	0.0027	0.01	No
Endrin	Bangangté	Cowpea	0.0020	0.01	No
Endrin	Foumban	Egusi seeds	0.0025	NA	No
Endrin	Bafoussam	Egusi seeds	0.0021	NA	No
Endrin	Bafang	Egusi seeds	0.0020	NA	No
Endrin	Mbouda	Egusi seeds	0.0017	NA	No
Endrin	Bafoussam	Groundnuts	0.0022	0.01	No
Endrin	Bangangté	Kidney beans	0.0116	0.01	Yes
Endrin	Dschang	Kidney beans	0.0043	0.01	No
Endrin	Bafang	Kidney beans	0.0034	0.01	No
Endrin	Foumban	Kidney beans	0.0025	0.01	No
Endrin	Bafang	Kidney beans	0.0020	0.01	No
Endrin	Bangangté	Kidney beans	0.0016	0.01	No
Endrin	Mbouda	Kidney beans	0.0014	0.01	No
Endrin	Foumban	Maize	0.0159	0.01	Yes
Endrin	Dschang	Maize	0.0065	0.01	No
Endrin	Bangangté	Maize	0.0028	0.01	No
Endrin	Dschang	Maize	0.0016	0.01	No
Endrin	Mbouda	Maize	0.0016	0.01	No
Endrin	Bafang	Maize	0.0013	0.01	No
Endrin	Bafoussam	Soybeans	0.0057	0.01	No
Endrin	Mbouda	Soybeans	0.0029	0.01	No
Endrin	Foumban	White pepper	0.0061	0.1	No
Epoxiconazole	Bafoussam	Bambara nuts	0.0033	NA	No
Epoxiconazole	Bafoussam	Cowpea	0.0021	0.05	No
Epoxiconazole	Foumban	Cowpea	0.0012	0.05	No
Epoxiconazole	Bangangté	Cowpea	0.0004	0.05	No
Epoxiconazole	Bafoussam	Egusi seeds	0.0049	NA	No
Epoxiconazole	Mbouda	Egusi seeds	0.0024	NA	No
Epoxiconazole	Foumban	Egusi seeds	0.0021	NA	No
Epoxiconazole	Bafoussam	Egusi seeds	0.0005	NA	No
Epoxiconazole	Mbouda	Kidney beans	0.0008	0.05	No
Epoxiconazole	Foumban	Kidney beans	0.0008	0.05	No
Epoxiconazole	Bafang	Kidney beans	0.0004	0.05	No
Epoxiconazole	Bafoussam	Maize	0.0176	0.1	No
Epoxiconazole	Bafoussam	Maize	0.0139	0.1	No
Epoxiconazole	Foumban	Maize	0.0118	0.1	No

Pesticide	Location	Food item	Mean (mg/kg)	EU MRL (mg/kg)	Mean > MRL?
Epoxiconazole	Bangangté	Maize	0.0032	0.1	No
Epoxiconazole	Mbouda	Maize	0.0031	0.1	No
Epoxiconazole	Bafang	Maize	0.0024	0.1	No
Epoxiconazole	Dschang	Maize	0.0018	0.1	No
Epoxiconazole	Bafang	Maize	0.0014	0.1	No
Epoxiconazole	Mbouda	Maize	0.0014	0.1	No
Epoxiconazole	Dschang	Maize	0.0011	0.1	No
Epoxiconazole	Bangangté	Maize	0.0010	0.1	No
Epoxiconazole	Bafoussam	Soybeans	0.0045	0.05	No
Epoxiconazole	Bafang	Soybeans	0.0010	0.05	No
Epoxiconazole	Mbouda	Soybeans	0.0004	0.05	No
Epoxiconazole	Dschang	Soybeans	0.0004	0.05	No
Fenamiphos	Foumban	Bambara nuts	0.0014	NA	No
Fenbuconazole	Bafang	Black beans	0.0009	0.5	No
Fenbuconazole	Bafoussam	Kidney beans	0.0015	0.5	No
Fenbuconazole	Bangangté	Kidney beans	0.0009	0.5	No
Fenoxycarb	Bafoussam	Egusi seeds	0.0013	NA	No
Fenpropimorf	Foumban	Maize	0.0013	0.01	No
Fenpropimorf	Mbouda	Maize	0.0010	0.01	No
Fenpropimorf	Bafoussam	Maize	0.0005	0.01	No
Fenpropimorf	Dschang	Maize	0.0005	0.01	No
Fenpropimorf	Dschang	Soybeans	0.0005	0.01	No
Fenpropimorf	Foumban	Soybeans	0.0004	0.01	No
Fenpropimorf	Bafoussam	White pepper	0.0012	0.05	No
Heptaclor	Bafang	Chili pepper	0.1236	0.1	Yes
Heptaclor	Foumban	Chili pepper	0.1196	0.1	Yes
Heptaclor	Dschang	Chili pepper	0.0110	0.1	No
Heptaclor	Bafoussam	Chili pepper	0.0081	0.1	No
Heptaclor	Bangangté	Chili pepper	0.0052	0.1	No
Heptaclor	Bangangté	Cocoa	0.0020	0.02	No
Heptaclor	Foumban	Coffee	0.0022	0.02	No
Heptaclor	Bafang	Coffee	0.0018	0.02	No
Heptaclor	Foumban	Coffee	0.0017	0.02	No
Heptaclor	Bafang	Egusi seeds	0.0020	NA	No
Heptaclor	Bangangté	Egusi seeds	0.0014	NA	No
Heptaclor	Mbouda	Groundnuts	0.0012	0.01	No
Heptaclor	Bafoussam	Groundnuts	0.0012	0.01	No
Heptaclor	Mbouda	Kidney beans	0.0062	0.01	No
Heptaclor	Bafang	Soybeans	0.0021	0.01	No
Heptaclor	Foumban	Soybeans	0.0012	0.01	No
Hexachlorobenzene	Bafoussam	Bambara nuts	0.0175	NA	No
Hexachlorobenzene	Foumban	Bambara nuts	0.0098	NA	No

Pesticide	Location	Food item	Mean (mg/kg)	EU MRL (mg/kg)	Mean > MRL?
Hexachlorobenzene	Bafang	Black beans	0.0102	0.01	Yes
Hexachlorobenzene	Mbouda	Black beans	0.0076	0.01	No
Hexachlorobenzene	Bafoussam	Chili pepper	0.2660	0.02	Yes
Hexachlorobenzene	Bafang	Chili pepper	0.0822	0.02	Yes
Hexachlorobenzene	Dschang	Chili pepper	0.0607	0.02	Yes
Hexachlorobenzene	Bafoussam	Chili pepper	0.0577	0.02	Yes
Hexachlorobenzene	Bangangté	Chili pepper	0.0421	0.02	Yes
Hexachlorobenzene	Foumban	Chili pepper	0.0418	0.02	Yes
Hexachlorobenzene	Bangangté	Cocoa	0.0365	0.02	Yes
Hexachlorobenzene	Bafang	Cocoa	0.0285	0.02	Yes
Hexachlorobenzene	Foumban	Coffee	0.1109	0.02	Yes
Hexachlorobenzene	Foumban	Coffee	0.0948	0.02	Yes
Hexachlorobenzene	Bangangté	Coffee	0.0527	0.02	Yes
Hexachlorobenzene	Bafang	Coffee	0.0308	0.02	Yes
Hexachlorobenzene	Foumban	Cowpea	0.0355	0.01	Yes
Hexachlorobenzene	Bangangté	Cowpea	0.0080	0.01	No
Hexachlorobenzene	Bafoussam	Cowpea	0.0038	0.01	No
Hexachlorobenzene	Bafoussam	Cowpea	0.0014	0.01	No
Hexachlorobenzene	Bangangté	Egusi seeds	0.0219	NA	No
Hexachlorobenzene	Bafoussam	Egusi seeds	0.0088	NA	No
Hexachlorobenzene	Bafoussam	Egusi seeds	0.0032	NA	No
Hexachlorobenzene	Mbouda	Groundnuts	0.1674	0.2	No
Hexachlorobenzene	Dschang	Groundnuts	0.1488	0.2	No
Hexachlorobenzene	Bangangté	Groundnuts	0.1443	0.2	No
Hexachlorobenzene	Mbouda	Groundnuts	0.0820	0.2	No
Hexachlorobenzene	Foumban	Groundnuts	0.0813	0.2	No
Hexachlorobenzene	Bafang	Groundnuts	0.0737	0.2	No
Hexachlorobenzene	Bafoussam	Groundnuts	0.0533	0.2	No
Hexachlorobenzene	Bafoussam	Kidney beans	3.0895	0.01	Yes
Hexachlorobenzene	Mbouda	Kidney beans	0.3032	0.01	Yes
Hexachlorobenzene	Bafang	Kidney beans	0.1985	0.01	Yes
Hexachlorobenzene	Dschang	Kidney beans	0.0464	0.01	Yes
Hexachlorobenzene	Foumban	Kidney beans	0.0052	0.01	No
Hexachlorobenzene	Dschang	Kidney beans	0.0036	0.01	No
Hexachlorobenzene	Bangangté	Kidney beans	0.0020	0.01	No
Hexachlorobenzene	Mbouda	Kidney beans	0.0019	0.01	No
Hexachlorobenzene	Bangangté	Kidney beans	0.0016	0.01	No
Hexachlorobenzene	Foumban	Maize	1.9048	0.01	Yes
Hexachlorobenzene	Bangangté	Maize	0.2135	0.01	Yes
Hexachlorobenzene	Bafoussam	Maize	0.0208	0.01	Yes
Hexachlorobenzene	Bafoussam	Maize	0.0120	0.01	Yes
Hexachlorobenzene	Dschang	Maize	0.0110	0.01	Yes

Pesticide	Location	Food item	Mean (mg/kg)	EU MRL (mg/kg)	Mean > MRL?
Hexachlorobenzene	Bangangté	Maize	0.0091	0.01	No
Hexachlorobenzene	Mbouda	Maize	0.0082	0.01	No
Hexachlorobenzene	Mbouda	Maize	0.0068	0.01	No
Hexachlorobenzene	Bafang	Maize	0.0064	0.01	No
Hexachlorobenzene	Dschang	Maize	0.0047	0.01	No
Hexachlorobenzene	Bafang	Maize	0.0045	0.01	No
Hexachlorobenzene	Dschang	Soybeans	0.0267	0.02	Yes
Hexachlorobenzene	Bafoussam	Soybeans	0.0154	0.02	No
Hexachlorobenzene	Foumban	Soybeans	0.0111	0.02	No
Hexachlorobenzene	Mbouda	Soybeans	0.0060	0.02	No
Hexachlorobenzene	Bafoussam	Soybeans	0.0051	0.02	No
Hexachlorobenzene	Foumban	White pepper	0.0487	0.02	Yes
Hexachlorobenzene	Bafoussam	White pepper	0.0151	0.02	No
Hexaconazole	Bafang	Coffee	0.0121	0.05	No
Hexaconazole	Bafoussam	Groundnuts	0.0025	0.02	No
Imazalil	Bafoussam	Cowpea	0.0032	0.05	No
Imidacloprid	Bafoussam	Black beans	0.0020	2	No
Imidacloprid	Bafoussam	Cowpea	0.0020	2	No
Imidacloprid	Bafoussam	Cowpea	0.0008	2	No
Imidacloprid	Bafoussam	Maize	0.0050	0.1	No
Imidacloprid	Mbouda	Soybeans	0.0120	0.05	No
Imidacloprid	Bafoussam	White pepper	0.0051	0.05	No
Isoproturon	Bafang	Black beans	0.0004	0.01	No
Linuron	Bafoussam	Cowpea	0.0036	0.05	No
Linuron	Foumban	Maize	0.1041	0.05	Yes
Linuron	Foumban	Soybeans	0.0024	0.01	No
Malathion	Bafoussam	Bambara nuts	0.5183	NA	No
Malathion	Bafang	Bambara nuts	0.2525	NA	No
Malathion	Bafoussam	Black beans	4.2119	0.02	Yes
Malathion	Bafang	Black beans	3.6157	0.02	Yes
Malathion	Mbouda	Black beans	3.2892	0.02	Yes
Malathion	Bangangté	Black beans	1.8794	0.02	Yes
Malathion	Bafoussam	Chili pepper	1.6661	0.02	Yes
Malathion	Bafoussam	Chili pepper	1.5791	0.02	Yes
Malathion	Foumban	Chili pepper	1.0121	0.02	Yes
Malathion	Bafang	Chili pepper	0.7615	0.02	Yes
Malathion	Mbouda	Chili pepper	0.4084	0.02	Yes
Malathion	Bangangté	Chili pepper	0.1607	0.02	Yes
Malathion	Dschang	Chili pepper	0.0481	0.02	Yes
Malathion	Bangangté	Cocoa	0.1088	0.02	Yes
Malathion	Bafang	Cocoa	0.0189	0.02	No
Malathion	Foumban	Coffee	0.5845	0.02	Yes

Pesticide	Location	Food item	Mean (mg/kg)	EU MRL (mg/kg)	Mean > MRL?
Malathion	Foumban	Coffee	0.1926	0.02	Yes
Malathion	Bafang	Coffee	0.1208	0.02	Yes
Malathion	Bangangté	Coffee	0.0789	0.02	Yes
Malathion	Bafoussam	Cowpea	0.9206	0.02	Yes
Malathion	Bafoussam	Cowpea	0.4761	0.02	Yes
Malathion	Mbouda	Cowpea	0.2658	0.02	Yes
Malathion	Bafang	Cowpea	0.2300	0.02	Yes
Malathion	Bangangté	Cowpea	0.1726	0.02	Yes
Malathion	Foumban	Cowpea	0.0557	0.02	Yes
Malathion	Dschang	Cowpea	0.0415	0.02	Yes
Malathion	Mbouda	Egusi seeds	4.8153	NA	No
Malathion	Bafoussam	Egusi seeds	3.8179	NA	No
Malathion	Bafoussam	Egusi seeds	2.8729	NA	No
Malathion	Mbouda	Egusi seeds	2.0124	NA	No
Malathion	Foumban	Egusi seeds	1.5177	NA	No
Malathion	Bafang	Egusi seeds	0.5792	NA	No
Malathion	Bangangté	Egusi seeds	0.0073	NA	No
Malathion	Mbouda	Groundnuts	0.5295	0.02	Yes
Malathion	Bangangté	Groundnuts	0.3305	0.02	Yes
Malathion	Bafang	Groundnuts	0.2968	0.02	Yes
Malathion	Dschang	Groundnuts	0.2796	0.02	Yes
Malathion	Mbouda	Groundnuts	0.1644	0.02	Yes
Malathion	Bafoussam	Groundnuts	0.1601	0.02	Yes
Malathion	Bafoussam	Groundnuts	0.1585	0.02	Yes
Malathion	Dschang	Groundnuts	0.0412	0.02	Yes
Malathion	Bafoussam	Kidney beans	5.5269	0.02	Yes
Malathion	Bangangté	Kidney beans	3.1351	0.02	Yes
Malathion	Mbouda	Kidney beans	2.9499	0.02	Yes
Malathion	Foumban	Kidney beans	2.2370	0.02	Yes
Malathion	Foumban	Kidney beans	2.0049	0.02	Yes
Malathion	Bafang	Kidney beans	1.7801	0.02	Yes
Malathion	Bangangté	Kidney beans	0.6728	0.02	Yes
Malathion	Bafang	Kidney beans	0.5518	0.02	Yes
Malathion	Dschang	Kidney beans	0.3496	0.02	Yes
Malathion	Mbouda	Kidney beans	0.2646	0.02	Yes
Malathion	Dschang	Kidney beans	0.0827	0.02	Yes
Malathion	Mbouda	Maize	0.6789	8	No
Malathion	Bangangté	Maize	0.1693	8	No
Malathion	Bafoussam	Maize	0.1560	8	No
Malathion	Bangangté	Maize	0.1363	8	No
Malathion	Mbouda	Maize	0.1359	8	No
Malathion	Bafoussam	Maize	0.1240	8	No

Pesticide	Location	Food item	Mean (mg/kg)	EU MRL (mg/kg)	Mean > MRL?
Malathion	Bafang	Maize	0.1034	8	No
Malathion	Foumban	Maize	0.0802	8	No
Malathion	Bafang	Maize	0.0739	8	No
Malathion	Dschang	Maize	0.0277	8	No
Malathion	Dschang	Maize	0.0235	8	No
Malathion	Bafoussam	Soybeans	0.7638	0.02	Yes
Malathion	Bafoussam	Soybeans	0.3984	0.02	Yes
Malathion	Mbouda	Soybeans	0.3365	0.02	Yes
Malathion	Bafang	Soybeans	0.1850	0.02	Yes
Malathion	Dschang	Soybeans	0.1746	0.02	Yes
Malathion	Foumban	Soybeans	0.1499	0.02	Yes
Malathion	Bafoussam	White pepper	3.2985	0.02	Yes
Metalaxyl	Bafoussam	Bambara nuts	0.0006	NA	No
Metalaxyl	Bafoussam	Black beans	0.0053	0.02	No
Metalaxyl	Bafang	Black beans	0.0004	0.02	No
Metalaxyl	Mbouda	Chili pepper	0.0018	0.1	No
Metalaxyl	Bafoussam	Chili pepper	0.0008	0.1	No
Metalaxyl	Bafang	Coffee	0.0007	0.05	No
Metalaxyl	Foumban	Maize	0.0006	0.02	No
Metalaxyl	Dschang	Soybeans	0.0005	0.1	No
Metalaxyl	Bafoussam	White pepper	0.1736	0.1	Yes
Methiocarb	Bafang	Chili pepper	0.0047	0.1	No
Methiocarb	Foumban	Maize	0.0061	0.1	No
Methiocarb	Bafoussam	Maize	0.0049	0.1	No
Methiocarb	Bafoussam	Maize	0.0015	0.1	No
Methoxychlor	Foumban	Chili pepper	0.1312	0.1	Yes
Methoxychlor	Bafang	Chili pepper	0.1144	0.1	Yes
Methoxychlor	Bangangté	Chili pepper	0.0486	0.1	No
Methoxychlor	Bafoussam	Chili pepper	0.0419	0.1	No
Methoxychlor	Bafang	Coffee	0.0160	0.1	No
Methoxychlor	Bafoussam	Cowpea	0.0255	0.1	No
Methoxychlor	Bafoussam	Maize	0.8165	0.01	Yes
Methoxychlor	Bafoussam	Maize	0.5488	0.01	Yes
Methoxychlor	Mbouda	Maize	0.0464	0.01	Yes
Methoxychlor	Bafoussam	Soybeans	0.0323	0.01	Yes
Methribuzin	Dschang	Soybeans	0.0037	0.1	No
Monocrotophos	Foumban	Chili pepper	0.0012	0.05	No
Monocrotophos	Bangangté	Cocoa	0.0079	0.05	No
Monocrotophos	Bafang	Coffee	0.0012	0.05	No
o,p'-DDT	Bafoussam	Bambara nuts	0.0059	NA	No
o,p'-DDT	Bafang	Bambara nuts	0.0038	NA	No
o,p'-DDT	Foumban	Bambara nuts	0.0016	NA	No

Pesticide	Location	Food item	Mean (mg/kg)	EU MRL (mg/kg)	Mean > MRL?
o,p'-DDT	Bafang	Black beans	0.0028	0.05	No
o,p'-DDT	Bafoussam	Black beans	0.0017	0.05	No
o,p'-DDT	Mbouda	Black beans	0.0015	0.05	No
o,p'-DDT	Bangangté	Black beans	0.0013	0.05	No
o,p'-DDT	Bafang	Chili pepper	0.0156	1	No
o,p'-DDT	Dschang	Chili pepper	0.0068	1	No
o,p'-DDT	Foumban	Chili pepper	0.0041	1	No
o,p'-DDT	Bangangté	Chili pepper	0.0027	1	No
o,p'-DDT	Bafoussam	Chili pepper	0.0027	1	No
o,p'-DDT	Bafoussam	Chili pepper	0.0026	1	No
o,p'-DDT	Dschang	Cowpea	0.0034	0.05	No
o,p'-DDT	Mbouda	Cowpea	0.0024	0.05	No
o,p'-DDT	Bafang	Cowpea	0.0020	0.05	No
o,p'-DDT	Foumban	Cowpea	0.0018	0.05	No
o,p'-DDT	Bafoussam	Cowpea	0.0018	0.05	No
o,p'-DDT	Bafoussam	Cowpea	0.0017	0.05	No
o,p'-DDT	Bangangté	Cowpea	0.0015	0.05	No
o,p'-DDT	Bafoussam	Egusi seeds	0.0131	NA	No
o,p'-DDT	Bafoussam	Egusi seeds	0.0099	NA	No
o,p'-DDT	Mbouda	Egusi seeds	0.0085	NA	No
o,p'-DDT	Foumban	Egusi seeds	0.0052	NA	No
o,p'-DDT	Bafang	Egusi seeds	0.0022	NA	No
o,p'-DDT	Bafoussam	Groundnuts	0.0043	0.05	No
o,p'-DDT	Foumban	Groundnuts	0.0028	0.05	No
o,p'-DDT	Bangangté	Kidney beans	0.0050	0.05	No
o,p'-DDT	Bafang	Kidney beans	0.0041	0.05	No
o,p'-DDT	Dschang	Kidney beans	0.0037	0.05	No
o,p'-DDT	Mbouda	Kidney beans	0.0021	0.05	No
o,p'-DDT	Mbouda	Kidney beans	0.0017	0.05	No
o,p'-DDT	Dschang	Kidney beans	0.0017	0.05	No
o,p'-DDT	Foumban	Kidney beans	0.0016	0.05	No
o,p'-DDT	Bafang	Kidney beans	0.0014	0.05	No
o,p'-DDT	Bangangté	Kidney beans	0.0013	0.05	No
o,p'-DDT	Bafoussam	Maize	0.0116	0.05	No
o,p'-DDT	Mbouda	Maize	0.0087	0.05	No
o,p'-DDT	Bafoussam	Maize	0.0084	0.05	No
o,p'-DDT	Bafang	Maize	0.0067	0.05	No
o,p'-DDT	Foumban	Maize	0.0051	0.05	No
o,p'-DDT	Dschang	Maize	0.0029	0.05	No
o,p'-DDT	Bangangté	Maize	0.0023	0.05	No
o,p'-DDT	Bafang	Maize	0.0019	0.05	No
o,p'-DDT	Bangangté	Maize	0.0017	0.05	No

Pesticide	Location	Food item	Mean (mg/kg)	EU MRL (mg/kg)	Mean > MRL?
o,p'-DDT	Dschang	Maize	0.0016	0.05	No
o,p'-DDT	Bafoussam	Soybeans	0.0035	0.05	No
o,p'-DDT	Bafang	Soybeans	0.0034	0.05	No
o,p'-DDT	Mbouda	Soybeans	0.0026	0.05	No
o,p'-DDT	Bafoussam	Soybeans	0.0015	0.05	No
o,p'-DDT	Foumban	White pepper	0.0121	1	No
p,p'-DDD	Bafoussam	Bambara nuts	0.0017	NA	No
p,p'-DDD	Bafang	Bambara nuts	0.0017	NA	No
p,p'-DDD	Bangangté	Black beans	0.0025	0.05	No
p,p'-DDD	Mbouda	Black beans	0.0016	0.05	No
p,p'-DDD	Bafoussam	Black beans	0.0014	0.05	No
p,p'-DDD	Bafoussam	Chili pepper	0.0103	1	No
p,p'-DDD	Bafang	Chili pepper	0.0021	1	No
p,p'-DDD	Bafoussam	Chili pepper	0.0018	1	No
p,p'-DDD	Bangangté	Chili pepper	0.0012	1	No
p,p'-DDD	Bangangté	Cocoa	0.0015	0.5	No
p,p'-DDD	Bangangté	Coffee	0.0026	1	No
p,p'-DDD	Bangangté	Cowpea	0.0026	0.05	No
p,p'-DDD	Mbouda	Cowpea	0.0020	0.05	No
p,p'-DDD	Bafang	Cowpea	0.0014	0.05	No
p,p'-DDD	Bafoussam	Cowpea	0.0013	0.05	No
p,p'-DDD	Bafoussam	Cowpea	0.0012	0.05	No
p,p'-DDD	Mbouda	Egusi seeds	0.0052	NA	No
p,p'-DDD	Bafang	Egusi seeds	0.0034	NA	No
p,p'-DDD	Foumban	Egusi seeds	0.0033	NA	No
p,p'-DDD	Bafoussam	Egusi seeds	0.0027	NA	No
p,p'-DDD	Bangangté	Egusi seeds	0.0022	NA	No
p,p'-DDD	Bafoussam	Egusi seeds	0.0016	NA	No
p,p'-DDD	Bafang	Groundnuts	0.0173	0.05	No
p,p'-DDD	Bafoussam	Groundnuts	0.0022	0.05	No
p,p'-DDD	Bangangté	Kidney beans	0.0027	0.05	No
p,p'-DDD	Bangangté	Kidney beans	0.0026	0.05	No
p,p'-DDD	Mbouda	Kidney beans	0.0022	0.05	No
p,p'-DDD	Bafang	Kidney beans	0.0022	0.05	No
p,p'-DDD	Dschang	Kidney beans	0.0022	0.05	No
p,p'-DDD	Mbouda	Kidney beans	0.0019	0.05	No
p,p'-DDD	Foumban	Kidney beans	0.0014	0.05	No
p,p'-DDD	Bafang	Kidney beans	0.0013	0.05	No
p,p'-DDD	Foumban	Maize	0.0103	0.05	No
p,p'-DDD	Mbouda	Maize	0.0051	0.05	No
p,p'-DDD	Dschang	Maize	0.0044	0.05	No
p,p'-DDD	Bangangté	Maize	0.0039	0.05	No

Pesticide	Location	Food item	Mean (mg/kg)	EU MRL (mg/kg)	Mean > MRL?
p,p'-DDD	Dschang	Maize	0.0037	0.05	No
p,p'-DDD	Bafang	Maize	0.0033	0.05	No
p,p'-DDD	Mbouda	Soybeans	0.0036	0.05	No
p,p'-DDD	Bafoussam	Soybeans	0.0022	0.05	No
p,p'-DDD	Bafoussam	Soybeans	0.0013	0.05	No
p,p'-DDD	Foumban	White pepper	0.0241	1	No
p,p'-DDE	Foumban	Bambara nuts	0.0276	NA	No
p,p'-DDE	Dschang	Black beans	0.0197	0.05	No
p,p'-DDE	Bangangté	Black beans	0.0193	0.05	No
p,p'-DDE	Mbouda	Chili pepper	0.0179	1	No
p,p'-DDE	Foumban	Chili pepper	0.0129	1	No
p,p'-DDE	Bangangté	Chili pepper	0.0084	1	No
p,p'-DDE	Bafang	Chili pepper	0.0080	1	No
p,p'-DDE	Foumban	Cocoa	0.0077	0.5	No
p,p'-DDE	Bafang	Cocoa	0.0071	0.5	No
p,p'-DDE	Bafang	Coffee	0.0066	1	No
p,p'-DDE	Bangangté	Coffee	0.0064	1	No
p,p'-DDE	Bafoussam	Coffee	0.0061	1	No
p,p'-DDE	Foumban	Coffee	0.0054	1	No
p,p'-DDE	Dschang	Egusi seeds	0.0042	NA	No
p,p'-DDE	Bangangté	Egusi seeds	0.0041	NA	No
p,p'-DDE	Bafang	Egusi seeds	0.0040	NA	No
p,p'-DDE	Bafoussam	Egusi seeds	0.0039	NA	No
p,p'-DDE	Foumban	Egusi seeds	0.0038	NA	No
p,p'-DDE	Bafoussam	Groundnuts	0.0036	0.05	No
p,p'-DDE	Bafoussam	Groundnuts	0.0036	0.05	No
p,p'-DDE	Bangangté	Groundnuts	0.0027	0.05	No
p,p'-DDE	Bafoussam	Groundnuts	0.0024	0.05	No
p,p'-DDE	Dschang	Groundnuts	0.0024	0.05	No
p,p'-DDE	Mbouda	Groundnuts	0.0023	0.05	No
p,p'-DDE	Bafoussam	Kidney beans	0.0022	0.05	No
p,p'-DDE	Mbouda	Kidney beans	0.0022	0.05	No
p,p'-DDE	Bafoussam	Kidney beans	0.0022	0.05	No
p,p'-DDE	Dschang	Kidney beans	0.0020	0.05	No
p,p'-DDE	Mbouda	Kidney beans	0.0019	0.05	No
p,p'-DDE	Foumban	Maize	0.0019	0.05	No
p,p'-DDE	Bafang	Maize	0.0019	0.05	No
p,p'-DDE	Bafoussam	Maize	0.0018	0.05	No
p,p'-DDE	Foumban	Maize	0.0018	0.05	No
p,p'-DDE	Bangangté	Maize	0.0018	0.05	No
p,p'-DDE	Bafang	Maize	0.0017	0.05	No
p,p'-DDE	Bangangté	Maize	0.0016	0.05	No

Pesticide	Location	Food item	Mean (mg/kg)	EU MRL (mg/kg)	Mean > MRL?
p,p'-DDE	Mbouda	Maize	0.0015	0.05	No
p,p'-DDE	Mbouda	Maize	0.0015	0.05	No
p,p'-DDE	Bafoussam	Maize	0.0015	0.05	No
p,p'-DDE	Bafang	Soybeans	0.0015	0.05	No
p,p'-DDE	Mbouda	Soybeans	0.0015	0.05	No
p,p'-DDE	Dschang	Soybeans	0.0014	0.05	No
p,p'-DDE	Dschang	Soybeans	0.0013	0.05	No
p,p'-DDE	Foumban	White pepper	0.0013	1	No
p,p'-DDT	Foumban	Bambara nuts	0.0550	NA	No
p,p'-DDT	Bafoussam	Bambara nuts	0.0221	NA	No
p,p'-DDT	Bafang	Bambara nuts	0.0156	NA	No
p,p'-DDT	Bafang	Black beans	0.1466	0.05	Yes
p,p'-DDT	Mbouda	Black beans	0.0931	0.05	Yes
p,p'-DDT	Bafoussam	Black beans	0.0597	0.05	Yes
p,p'-DDT	Bangangté	Black beans	0.0381	0.05	No
p,p'-DDT	Bafoussam	Chili pepper	0.0989	1	No
p,p'-DDT	Bafang	Chili pepper	0.0146	1	No
p,p'-DDT	Foumban	Chili pepper	0.0137	1	No
p,p'-DDT	Bafoussam	Chili pepper	0.0134	1	No
p,p'-DDT	Dschang	Chili pepper	0.0125	1	No
p,p'-DDT	Bangangté	Chili pepper	0.0099	1	No
p,p'-DDT	Bafang	Cocoa	0.0308	0.5	No
p,p'-DDT	Bangangté	Cocoa	0.0067	0.5	No
p,p'-DDT	Bangangté	Coffee	0.0043	1	No
p,p'-DDT	Bafang	Coffee	0.0036	1	No
p,p'-DDT	Foumban	Coffee	0.0033	1	No
p,p'-DDT	Dschang	Cowpea	0.0152	0.05	No
p,p'-DDT	Bangangté	Cowpea	0.0151	0.05	No
p,p'-DDT	Mbouda	Cowpea	0.0125	0.05	No
p,p'-DDT	Bafoussam	Cowpea	0.0114	0.05	No
p,p'-DDT	Bafoussam	Cowpea	0.0112	0.05	No
p,p'-DDT	Bafang	Cowpea	0.0103	0.05	No
p,p'-DDT	Foumban	Egusi seeds	0.0472	NA	No
p,p'-DDT	Bafoussam	Egusi seeds	0.0378	NA	No
p,p'-DDT	Bafoussam	Egusi seeds	0.0177	NA	No
p,p'-DDT	Mbouda	Egusi seeds	0.0124	NA	No
p,p'-DDT	Mbouda	Egusi seeds	0.0099	NA	No
p,p'-DDT	Bafang	Egusi seeds	0.0083	NA	No
p,p'-DDT	Bangangté	Egusi seeds	0.0079	NA	No
p,p'-DDT	Bafoussam	Groundnuts	0.0086	0.05	No
p,p'-DDT	Bafoussam	Groundnuts	0.0081	0.05	No
p,p'-DDT	Mbouda	Groundnuts	0.0078	0.05	No

Pesticide	Location	Food item	Mean (mg/kg)	EU MRL (mg/kg)	Mean > MRL?
p,p'-DDT	Foumban	Groundnuts	0.0069	0.05	No
p,p'-DDT	Bafang	Groundnuts	0.0068	0.05	No
p,p'-DDT	Mbouda	Groundnuts	0.0066	0.05	No
p,p'-DDT	Dschang	Groundnuts	0.0063	0.05	No
p,p'-DDT	Bangangté	Groundnuts	0.0058	0.05	No
p,p'-DDT	Bafoussam	Kidney beans	0.0856	0.05	Yes
p,p'-DDT	Mbouda	Kidney beans	0.0755	0.05	Yes
p,p'-DDT	Bafang	Kidney beans	0.0688	0.05	Yes
p,p'-DDT	Bangangté	Kidney beans	0.0637	0.05	Yes
p,p'-DDT	Foumban	Kidney beans	0.0369	0.05	No
p,p'-DDT	Dschang	Kidney beans	0.0211	0.05	No
p,p'-DDT	Bangangté	Kidney beans	0.0180	0.05	No
p,p'-DDT	Mbouda	Kidney beans	0.0175	0.05	No
p,p'-DDT	Dschang	Kidney beans	0.0159	0.05	No
p,p'-DDT	Mbouda	Kidney beans	0.0142	0.05	No
p,p'-DDT	Bafang	Kidney beans	0.0126	0.05	No
p,p'-DDT	Dschang	Kidney beans	0.0072	0.05	No
p,p'-DDT	Mbouda	Maize	0.0322	0.05	No
p,p'-DDT	Bafoussam	Maize	0.0287	0.05	No
p,p'-DDT	Mbouda	Maize	0.0240	0.05	No
p,p'-DDT	Bafoussam	Maize	0.0232	0.05	No
p,p'-DDT	Dschang	Maize	0.0213	0.05	No
p,p'-DDT	Dschang	Maize	0.0202	0.05	No
p,p'-DDT	Bangangté	Maize	0.0202	0.05	No
p,p'-DDT	Bangangté	Maize	0.0199	0.05	No
p,p'-DDT	Bafang	Maize	0.0185	0.05	No
p,p'-DDT	Bafang	Maize	0.0141	0.05	No
p,p'-DDT	Foumban	Maize	0.0132	0.05	No
p,p'-DDT	Bafoussam	Soybeans	0.0148	0.05	No
p,p'-DDT	Mbouda	Soybeans	0.0146	0.05	No
p,p'-DDT	Bafoussam	Soybeans	0.0142	0.05	No
p,p'-DDT	Bafang	Soybeans	0.0136	0.05	No
p,p'-DDT	Foumban	Soybeans	0.0066	0.05	No
p,p'-DDT	Dschang	Soybeans	0.0045	0.05	No
p,p'-DDT	Foumban	White pepper	0.0397	1	No
Penconazole	Bangangté	Chili pepper	0.0214	0.1	No
Penconazole	Foumban	Chili pepper	0.0135	0.1	No
Penconazole	Bafoussam	Chili pepper	0.0098	0.1	No
Penconazole	Mbouda	Chili pepper	0.0081	0.1	No
Penconazole	Bafoussam	Chili pepper	0.0062	0.1	No
Penconazole	Bafoussam	White pepper	0.0155	0.1	No
Penconazole	Foumban	White pepper	0.0068	0.1	No

Pesticide	Location	Food item	Mean (mg/kg)	EU MRL (mg/kg)	Mean > MRL?
Pirimiphos-methyl	Bafoussam	Bambara nuts	0.0005	NA	No
Pirimiphos-methyl	Bangangté	Black beans	0.0260	0.01	Yes
Pirimiphos-methyl	Mbouda	Black beans	0.0006	0.01	No
Pirimiphos-methyl	Mbouda	Chili pepper	0.0062	0.5	No
Pirimiphos-methyl	Foumban	Chili pepper	0.0058	0.5	No
Pirimiphos-methyl	Bangangté	Chili pepper	0.0046	0.5	No
Pirimiphos-methyl	Bafoussam	Chili pepper	0.0026	0.5	No
Pirimiphos-methyl	Bafoussam	Chili pepper	0.0024	0.5	No
Pirimiphos-methyl	Bangangté	Coffee	0.0074	0.05	No
Pirimiphos-methyl	Bafang	Coffee	0.0066	0.05	No
Pirimiphos-methyl	Bafang	Coffee	0.0028	0.05	No
Pirimiphos-methyl	Bafang	Coffee	0.0004	0.05	No
Pirimiphos-methyl	Bangangté	Cowpea	0.2735	0.01	Yes
Pirimiphos-methyl	Dschang	Cowpea	0.0797	0.01	Yes
Pirimiphos-methyl	Bafoussam	Cowpea	0.0032	0.01	No
Pirimiphos-methyl	Mbouda	Cowpea	0.0032	0.01	No
Pirimiphos-methyl	Bafoussam	Cowpea	0.0006	0.01	No
Pirimiphos-methyl	Bafoussam	Egusi seeds	0.0339	NA	No
Pirimiphos-methyl	Foumban	Egusi seeds	0.0206	NA	No
Pirimiphos-methyl	Mbouda	Egusi seeds	0.0109	NA	No
Pirimiphos-methyl	Mbouda	Egusi seeds	0.0101	NA	No
Pirimiphos-methyl	Bangangté	Egusi seeds	0.0085	NA	No
Pirimiphos-methyl	Bafoussam	Egusi seeds	0.0044	NA	No
Pirimiphos-methyl	Bafang	Egusi seeds	0.0016	NA	No
Pirimiphos-methyl	Bangangté	Groundnuts	0.0040	0.5	No
Pirimiphos-methyl	Dschang	Groundnuts	0.0031	0.5	No
Pirimiphos-methyl	Mbouda	Groundnuts	0.0018	0.5	No
Pirimiphos-methyl	Foumban	Groundnuts	0.0008	0.5	No
Pirimiphos-methyl	Bafoussam	Groundnuts	0.0006	0.5	No
Pirimiphos-methyl	Mbouda	Groundnuts	0.0005	0.5	No
Pirimiphos-methyl	Foumban	Kidney beans	0.0443	0.01	Yes
Pirimiphos-methyl	Mbouda	Kidney beans	0.0353	0.01	Yes
Pirimiphos-methyl	Mbouda	Kidney beans	0.0226	0.01	Yes
Pirimiphos-methyl	Dschang	Kidney beans	0.0031	0.01	No
Pirimiphos-methyl	Foumban	Kidney beans	0.0023	0.01	No
Pirimiphos-methyl	Bangangté	Kidney beans	0.0020	0.01	No
Pirimiphos-methyl	Dschang	Kidney beans	0.0009	0.01	No
Pirimiphos-methyl	Bafoussam	Kidney beans	0.0005	0.01	No
Pirimiphos-methyl	Bangangté	Maize	0.0028	0.5	No
Pirimiphos-methyl	Dschang	Maize	0.0005	0.5	No
Pirimiphos-methyl	Dschang	Maize	0.0005	0.5	No
Pirimiphos-methyl	Bangangté	Maize	0.0004	0.5	No

Pesticide	Location	Food item	Mean (mg/kg)	EU MRL (mg/kg)	Mean > MRL?
Pirimiphos-methyl	Dschang	Soybeans	0.0011	0.5	No
Pirimiphos-methyl	Bangangté	Soybeans	0.0008	0.5	No
Pirimiphos-methyl	Mbouda	Soybeans	0.0006	0.5	No
Pirimiphos-methyl	Bafoussam	Soybeans	0.0006	0.5	No
Pirimiphos-methyl	Bafoussam	White pepper	0.0200	0.5	No
Pirimiphos-methyl	Foumban	White pepper	0.0035	0.5	No
Propazine	Mbouda	Chili pepper	0.0032	NA	No
Propiconazole	Bafang	Kidney beans	0.0004	0.01	No
Propiconazole	Mbouda	Maize	0.0032	0.05	No
Propiconazole	Bafang	Maize	0.0016	0.05	No
Propoxur	Bangangté	Chili pepper	0.0006	0.1	No
Propoxur	Bafoussam	White pepper	0.0017	0.1	No
Pyrimethanil	Bafang	Black beans	0.0062	0.5	No
Pyrimethanil	Foumban	Cowpea	0.0152	0.5	No
Pyrimethanil	Foumban	Kidney beans	0.0929	0.5	No
Pyrimethanil	Foumban	Maize	0.0344	0.01	Yes
Simazine	Bafoussam	White pepper	0.0005	0.05	No
Tebuconazole	Bafoussam	White pepper	0.0016	0.05	No
Tebufenozide	Bafoussam	Bambara nuts	0.0006	NA	No
Tebufenozide	Foumban	Chili pepper	0.0004	1	No
Tebufenozide	Bafoussam	Egusi seeds	0.0023	NA	No
Tebufenozide	Mbouda	Egusi seeds	0.0012	NA	No
Tebufenozide	Mbouda	Egusi seeds	0.0010	NA	No
Tebufenozide	Bafoussam	Egusi seeds	0.0007	NA	No
Tebufenozide	Bafang	Kidney beans	0.0007	0.5	No
Tebufenozide	Bangangté	Kidney beans	0.0006	0.5	No
Tebufenozide	Bafoussam	Soybeans	0.0048	0.5	No
Terbuthryn	Bafoussam	White pepper	0.0041	NA	No
Terbuthylazine	Bafang	Bambara nuts	0.0260	NA	No
Terbuthylazine	Bafoussam	Bambara nuts	0.0102	NA	No
Terbuthylazine	Bafang	Black beans	0.0260	0.05	No
Terbuthylazine	Bangangté	Black beans	0.0226	0.05	No
Terbuthylazine	Bafoussam	Black beans	0.0174	0.05	No
Terbuthylazine	Dschang	Chili pepper	0.0224	0.05	No
Terbuthylazine	Mbouda	Chili pepper	0.0183	0.05	No
Terbuthylazine	Bafoussam	Chili pepper	0.0111	0.05	No
Terbuthylazine	Foumban	Chili pepper	0.0065	0.05	No
Terbuthylazine	Bafoussam	Chili pepper	0.0053	0.05	No
Terbuthylazine	Bangangté	Cocoa	0.0153	0.05	No
Terbuthylazine	Bafang	Cocoa	0.0098	0.05	No
Terbuthylazine	Bafang	Coffee	0.0451	0.05	No
Terbuthylazine	Bafoussam	Cowpea	0.1806	0.05	Yes

Pesticide	Location	Food item	Mean (mg/kg)	EU MRL (mg/kg)	Mean > MRL?
Terbuthylazine	Bafang	Cowpea	0.1417	0.05	Yes
Terbuthylazine	Foumban	Cowpea	0.0556	0.05	Yes
Terbuthylazine	Bafoussam	Cowpea	0.0368	0.05	No
Terbuthylazine	Bangangté	Cowpea	0.0366	0.05	No
Terbuthylazine	Mbouda	Cowpea	0.0335	0.05	No
Terbuthylazine	Dschang	Cowpea	0.0295	0.05	No
Terbuthylazine	Mbouda	Egusi seeds	0.0207	NA	No
Terbuthylazine	Bafoussam	Egusi seeds	0.0079	NA	No
Terbuthylazine	Bafoussam	Groundnuts	0.0104	0.1	No
Terbuthylazine	Bafang	Kidney beans	0.1878	0.05	Yes
Terbuthylazine	Bangangté	Kidney beans	0.1794	0.05	Yes
Terbuthylazine	Foumban	Kidney beans	0.0996	0.05	Yes
Terbuthylazine	Bafang	Kidney beans	0.0827	0.05	Yes
Terbuthylazine	Dschang	Kidney beans	0.0700	0.05	Yes
Terbuthylazine	Bafoussam	Kidney beans	0.0442	0.05	No
Terbuthylazine	Mbouda	Kidney beans	0.0396	0.05	No
Terbuthylazine	Foumban	Kidney beans	0.0365	0.05	No
Terbuthylazine	Dschang	Maize	0.0854	0.1	No
Terbuthylazine	Foumban	Maize	0.0749	0.1	No
Terbuthylazine	Dschang	Maize	0.0319	0.1	No
Terbuthylazine	Mbouda	Maize	0.0275	0.1	No
Terbuthylazine	Mbouda	Soybeans	0.0281	0.1	No
Terbuthylazine	Bafang	Soybeans	0.0252	0.1	No
Terbuthylazine	Bafoussam	Soybeans	0.0238	0.1	No
Terbuthylazine	Dschang	Soybeans	0.0172	0.1	No
Terbuthylazine	Bangangté	Soybeans	0.0117	0.1	No
Terbuthylazine	Foumban	Soybeans	0.0079	0.1	No
Thiofanate-methyl	Foumban	Kidney beans	0.0017	0.1	No
Thiofanate-methyl	Bafang	Kidney beans	0.0013	0.1	No
Thiofanate-methyl	Bafoussam	White pepper	0.0180	0.1	No
Triazophos	Foumban	Maize	0.0020	0.02	No
Triazophos	Mbouda	Soybeans	0.0013	0.01	No
Trifloxystrobin	Foumban	Kidney beans	0.0012	0.01	No
α-Endosulfan	Bafoussam	Bambara nuts	0.0104	NA	No
α-Endosulfan	Bafang	Bambara nuts	0.0053	NA	No
α-Endosulfan	Foumban	Bambara nuts	0.0025	NA	No
α-Endosulfan	Bangangté	Black beans	0.0229	0.05	No
α-Endosulfan	Bafang	Black beans	0.0068	0.05	No
α-Endosulfan	Bafoussam	Black beans	0.0034	0.05	No
α-Endosulfan	Mbouda	Black beans	0.0030	0.05	No
α-Endosulfan	Foumban	Chili pepper	0.0415	5	No
α-Endosulfan	Bafoussam	Chili pepper	0.0053	5	No

Pesticide	Location	Food item	Mean (mg/kg)	EU MRL (mg/kg)	Mean > MRL?
α-Endosulfan	Bafoussam	Chili pepper	0.0051	5	No
α-Endosulfan	Bangangté	Chili pepper	0.0038	5	No
α-Endosulfan	Bafang	Chili pepper	0.0030	5	No
α-Endosulfan	Bangangté	Cocoa	0.0022	0.1	No
α-Endosulfan	Bafang	Cocoa	0.0012	0.1	No
α-Endosulfan	Foumban	Coffee	0.0049	0.1	No
α-Endosulfan	Foumban	Coffee	0.0047	0.1	No
α-Endosulfan	Bangangté	Coffee	0.0025	0.1	No
α-Endosulfan	Bafang	Coffee	0.0022	0.1	No
α-Endosulfan	Bafoussam	Cowpea	0.0085	0.05	No
α-Endosulfan	Mbouda	Cowpea	0.0077	0.05	No
α-Endosulfan	Bangangté	Cowpea	0.0060	0.05	No
α-Endosulfan	Bafoussam	Cowpea	0.0044	0.05	No
α-Endosulfan	Dschang	Cowpea	0.0034	0.05	No
α-Endosulfan	Bafoussam	Egusi seeds	0.0136	NA	No
α-Endosulfan	Bafoussam	Egusi seeds	0.0099	NA	No
α-Endosulfan	Bafang	Egusi seeds	0.0041	NA	No
α-Endosulfan	Mbouda	Egusi seeds	0.0026	NA	No
α-Endosulfan	Bangangté	Egusi seeds	0.0013	NA	No
α-Endosulfan	Mbouda	Egusi seeds	0.0012	NA	No
α-Endosulfan	Mbouda	Groundnuts	0.0218	0.1	No
α-Endosulfan	Bafoussam	Groundnuts	0.0163	0.1	No
α-Endosulfan	Mbouda	Groundnuts	0.0108	0.1	No
α-Endosulfan	Foumban	Groundnuts	0.0096	0.1	No
α-Endosulfan	Bafoussam	Groundnuts	0.0038	0.1	No
α-Endosulfan	Bafang	Groundnuts	0.0030	0.1	No
α-Endosulfan	Mbouda	Kidney beans	0.0385	0.05	No
α-Endosulfan	Bangangté	Kidney beans	0.0158	0.05	No
α-Endosulfan	Dschang	Kidney beans	0.0156	0.05	No
α-Endosulfan	Dschang	Kidney beans	0.0076	0.05	No
α-Endosulfan	Bangangté	Kidney beans	0.0058	0.05	No
α-Endosulfan	Foumban	Kidney beans	0.0035	0.05	No
α-Endosulfan	Bafang	Kidney beans	0.0035	0.05	No
α-Endosulfan	Mbouda	Kidney beans	0.0030	0.05	No
α-Endosulfan	Dschang	Kidney beans	0.0022	0.05	No
α-Endosulfan	Bafang	Kidney beans	0.0019	0.05	No
α-Endosulfan	Mbouda	Kidney beans	0.0014	0.05	No
α-Endosulfan	Dschang	Maize	0.0148	0.05	No
α-Endosulfan	Bafoussam	Maize	0.0143	0.05	No
α-Endosulfan	Foumban	Maize	0.0099	0.05	No
α-Endosulfan	Bafoussam	Maize	0.0086	0.05	No
α-Endosulfan	Bangangté	Maize	0.0067	0.05	No

Pesticide	Location	Food item	Mean (mg/kg)	EU MRL (mg/kg)	Mean > MRL?
α-Endosulfan	Bangangté	Maize	0.0060	0.05	No
α-Endosulfan	Bafang	Maize	0.0033	0.05	No
α-Endosulfan	Dschang	Maize	0.0026	0.05	No
α-Endosulfan	Mbouda	Maize	0.0019	0.05	No
α-Endosulfan	Bafang	Maize	0.0016	0.05	No
α-Endosulfan	Bafoussam	Soybeans	0.0209	0.5	No
α-Endosulfan	Dschang	Soybeans	0.0056	0.5	No
α-Endosulfan	Bafoussam	Soybeans	0.0053	0.5	No
α-Endosulfan	Mbouda	Soybeans	0.0040	0.5	No
α-Endosulfan	Bafang	Soybeans	0.0031	0.5	No
α-Endosulfan	Foumban	Soybeans	0.0013	0.5	No
α-Endosulfan	Bafoussam	White pepper	0.0135	5	No
β-Endosulfan	Foumban	Coffee	0.0017	0.1	No
β-HCH	Foumban	Bambara nuts	0.0136	NA	No
β-HCH	Bangangté	Black beans	0.0040	0.01	No
β-HCH	Foumban	Chili pepper	0.1371	0.01	Yes
β-HCH	Bafang	Chili pepper	0.1305	0.01	Yes
β-HCH	Bangangté	Chili pepper	0.0809	0.01	Yes
β-HCH	Bafoussam	Chili pepper	0.0232	0.01	Yes
β-HCH	Dschang	Chili pepper	0.0184	0.01	Yes
β-HCH	Bafoussam	Chili pepper	0.0036	0.01	No
β-HCH	Bangangté	Cocoa	0.0212	0.01	Yes
β-HCH	Bafang	Cocoa	0.0077	0.01	No
β-HCH	Foumban	Coffee	0.0098	0.01	No
β-HCH	Foumban	Coffee	0.0081	0.01	No
β-HCH	Bafang	Coffee	0.0074	0.01	No
β-HCH	Bangangté	Coffee	0.0055	0.01	No
β-HCH	Foumban	Cowpea	0.1231	0.01	Yes
β-HCH	Bafoussam	Cowpea	0.0669	0.01	Yes
β-HCH	Bafoussam	Cowpea	0.0371	0.01	Yes
β-HCH	Dschang	Cowpea	0.0232	0.01	Yes
β-HCH	Bafang	Cowpea	0.0159	0.01	Yes
β-HCH	Bangangté	Cowpea	0.0129	0.01	Yes
β-HCH	Mbouda	Cowpea	0.0119	0.01	Yes
β-HCH	Foumban	Egusi seeds	0.0788	NA	No
β-HCH	Bafoussam	Egusi seeds	0.0123	NA	No
β-HCH	Bafoussam	Egusi seeds	0.0075	NA	No
β-HCH	Bafang	Egusi seeds	0.0060	NA	No
β-HCH	Bangangté	Egusi seeds	0.0060	NA	No
β-HCH	Mbouda	Egusi seeds	0.0042	NA	No
β-HCH	Mbouda	Groundnuts	0.0066	0.01	No
β-HCH	Bafoussam	Groundnuts	0.0057	0.01	No

Pesticide	Location	Food item	Mean (mg/kg)	EU MRL (mg/kg)	Mean > MRL?
β-HCH	Mbouda	Kidney beans	0.0570	0.01	Yes
β-HCH	Mbouda	Kidney beans	0.0122	0.01	Yes
β-HCH	Bangangté	Kidney beans	0.0050	0.01	No
β-HCH	Bangangté	Kidney beans	0.0027	0.01	No
β-HCH	Dschang	Kidney beans	0.0019	0.01	No
β-HCH	Foumban	Maize	0.0136	0.01	Yes
β-HCH	Bafoussam	Maize	0.0082	0.01	No
β-HCH	Bangangté	Maize	0.0065	0.01	No
β-HCH	Bafang	Maize	0.0063	0.01	No
β-HCH	Mbouda	Maize	0.0048	0.01	No
β-HCH	Bafoussam	Maize	0.0047	0.01	No
β-HCH	Mbouda	Soybeans	0.0498	0.01	Yes
β-HCH	Bafoussam	Soybeans	0.0445	0.01	Yes
β-HCH	Bafang	Soybeans	0.0335	0.01	Yes
β-HCH	Dschang	Soybeans	0.0068	0.01	No
β-HCH	Foumban	Soybeans	0.0033	0.01	No
β-HCH	Foumban	White pepper	0.1076	0.01	Yes
β-HCH	Bafoussam	White pepper	0.0307	0.01	Yes
